# Improving protein complex prediction by reconstructing a high-confidence protein-protein interaction network of Escherichia coli from different physical interaction data sources

**DOI:** 10.1186/s12859-016-1422-x

**Published:** 2017-01-03

**Authors:** Shirin Taghipour, Peyman Zarrineh, Mohammad Ganjtabesh, Abbas Nowzari-Dalini

**Affiliations:** Department of Computer Science, School of Mathematics, Statistics, and Computer Science, University of Tehran, P.O.Box: 14155-6455, Tehran, Iran

**Keywords:** Protein-protein interaction networks, Protein complexes, Biological networks, Network clustering, Functional networks

## Abstract

**Background:**

Although different protein-protein physical interaction (PPI) datasets exist for *Escherichia coli*, no common methodology exists to integrate these datasets and extract reliable modules reflecting the existing biological process and protein complexes. Naïve Bayesian formula is the highly accepted method to integrate different PPI datasets into a single weighted PPI network, but detecting proper weights in such network is still a major problem.

**Results:**

In this paper, we proposed a new methodology to integrate various physical PPI datasets into a single weighted PPI network in a way that the detected modules in PPI network exhibit the highest similarity to available functional modules. We used the co-expression modules as functional modules, and we shown that direct functional modules detected from Gene Ontology terms could be used as an alternative dataset. After running this integrating methodology over six different physical PPI datasets, orthologous high-confidence interactions from a related organism and two AP-MS PPI datasets gained high weights in the integrated networks, while the weights for one AP-MS PPI dataset and two other datasets derived from public databases have converged to zero. The majority of detected modules shaped around one or few hub protein(s). Still, a large number of highly interacting protein modules were detected which are functionally relevant and are likely to construct protein complexes.

**Conclusions:**

We provided a new high confidence protein complex prediction method supported by functional studies and literature mining.

**Electronic supplementary material:**

The online version of this article (doi:10.1186/s12859-016-1422-x) contains supplementary material, which is available to authorized users.

## Background

With the advent of new technologies and computational methods, large set of pair-wise physical protein-protein interactions and protein complexes have became available. The pair-wise interactions can be obtained through high throughput interactions [1–4], public databases such as DIP [3, 5], 3D structure based interactions including the prediction based on domain-domain interactions and docking [2, 3, 5–8], and homologous pairs of known interacting proteins [3, 9]. For example, for a well-studied organism like *E. coli* three large datasets of pair-wise protein-protein interactions are available which are measured by high-throughput tandem affinity purification followed by mass spectrometry (AP-MS) [1, 10, 11]. In addition, a large set of protein-protein interactions derived by yeast two-hybrid (Y2H) method has also recently became available for *E. coli* [12]. Furthermore, a large set of experimentally validated protein complexes has been registered for this organism in EcoCyc database [13]. Availability of large set of pair-wise physical protein-protein interactions, protein complexes, and protein functions for different organisms have facilitated the study of relations among them. It is generally accepted that interacting proteins are more likely to be functionally related, and a set of highly interacting proteins are more likely to constitute protein complexes. A natural way to represent protein-protein interactions relations passes through a network: each node corresponds to a protein and each undirected connection corresponds to a protein-protein interaction. This protein-protein interaction (PPI) network can be unweighted in the case that all the interactions come from a reliable data source, or it can be weighted if a certain value of confidence would be assigned to each connection. Different clustering and classification methods have been applied over PPI networks and the results have been compared to known protein complexes and functional categories [3]. MCL [14], clique percolation method [15, 16], and ClusterONE aglorithm [17] are among the most famous clustering methods. Naïve Bayesian is the most common classifier so far that has been applied over PPI network [1, 4–6].

In naïve Bayesian classifier, a confidence score is usually assigned to each protein pair through the naïve Bayesian formula. Through the naïve Bayesian procedure, a confidence score is assigned to each of the used datasets. This procedure needs to preset gold standard positive and negative sets. Positive sets consist of functionally related and probably experimentally validated interacting proteins. Determining negative set is less straight-forward. To build negative set protein pairs with unrelated function [1, 4, 5], locating in different part of the cell (different localization) [18] and non-interacting proteins from negatome database [3] have been used. Although, proteins in negative set are less likely to interact, interaction among protein pairs in negative set is still plausible. Small number of experimentally validated interacting proteins is another shortcoming of this procedure because positive set may include proteins for which physical interactions are highly understudied.

Due to the mentioned problems, some studies just focused on high-confidence protein-protein interactions data which are small set of all possible interactions and this data was further validated with functional data sources such as co-expression and literature mining [19]. PPI network study in Mycoplasma Pneumonia along with CompPASS [20] and ComPLEAT [21] methods are three examples of successful PPI studies.

In this study, we introduce a new methodology to integrate different protein-protein interaction datasets into a reliable weighted PPI network. Here, the weight of each protein pair is calculated through the highly used naïve Bayesian formula and frequently used MCL clustering method is employed afterward to determine protein modules from this weighted network. The similarity between the detected modules and provided functional modules are used as the criteria for optimization. Modules deleted from PPI network can be compared with the other functional module sets using the Normalized Mutual Information (NMI) measure. The main idea is that characterizing the best confidence score for protein-protein interaction datasets will maximize the similarity of detected modules from the PPI network with the other functional modules. This obviates the usage of problematic gold standard. We characterized the best confidence score of dataset using a global optimization method referred to as Harmony search. Using this methodology we integrated a large set of physical protein-protein interaction datasets of *E. coli* into a single weighted PPI network and we detected functionally relevant PPI modules which lead to new predictions in protein complexes.

## Methods

### PPI datasets

Three AP-MS high-throughput experiments of protein-protein interactions datasets were downloaded from the original papers [1, 10, 11]. Experimentally validated protein-protein interactions were downloaded from DIP database [22]. Protein-protein interactions from two databases, namely BIND [23] and IntAct [24], were also downloaded directly from the mentioned databases. High confidence protein-protein interactions from the evolutionary related organism *Mycoplasma Pneumonia* were retrieved from the original paper [19] and their orthologous proteins in *E. coli*, derived from co-complex database [13], were used as another data source. Finally protein complexes in *E. coli* were downloaded from EcoCyc database [13] and all the protein pairs in these complexes were considered as co-complex proteins. The mentioned data sources were used to build a weighted PPI network. All these datasets were downloaded on 18 January 2013. More recently released yeast two-hybrid (Y2H) PPI dataset experiments in *E. coli* [12] was downloaded from the original paper, but this dataset was just used for the validation part.

### Co-expression, Co-function, and Co-regulation modules

Co-expression, Co-function, and Co-regulation modules are derived as explained in Additional file 1.

### Reconstructing weighted PPI network

To integrate PPI data sources into a single weighted PPI network, we used the confidence score for each pair of proteins through the confidence score of each data source and the naïve Bayesian formula as follows:

1 $$ Similarity \left(p_{i}, p_{j}\right)=1- \prod_{p}\left(1-S_{p}\left(p_{i},p_{j}\right)\right),  $$

where *p*_*i*_ stands for the *i*th protein and *S*_*p*_ shows the confidence score (weight) of the *p*th dataset. This score is between 0 and 1. Therefore, *S*_*p*_(*p*_*i*_,*p*_*j*_) is zero if the interaction was not predicted in the *p*th dataset, and it is equal to *S*_*p*_ otherwise. Consequently, the similarity between two proteins is zero if their interaction is not predicted by any dataset. This similarity increases proportional to the confidence of each dataset for which the interaction was predicted. In addition, as more datasets predict a certain interaction, the corresponding similarity value increases. We just optimized the confidence scores *S*_*p*_ for each PPI dataset by a global optimization method in such a way that the detected modules in the integrated PPI network show the highest similarity with the co-expression, co-function, or co-regulation modules (see the next part). For this aim, we considered the weights between co-complex proteins or interacting proteins in DIP dataset as 1, and the remaining weights were optimized.

### Detecting optimized modules in the weighted PPI network

MCL method [14] was used to detect PPI modules in each iteration of optimization. Normalized Mutual Information (NMI) measure [25, 26] was used to compare the detected modules from PPI network with external functional module sets (co-expression, co-function, or co-regulation modules). A Harmony search method was then used to find the optimized weight scores of data sources in the confidence score formula in such a way that largest NMI value would be retrieved.

The Harmony search is a metaheuristic optimization algorithm, inspired by the underlying principles of the musicians’ improvisation of the harmony. There are usually three possible choices for a musician to compose a harmony: 1) play any famous piece of music exactly from their memory; 2) play something similar to a known piece (thus adjusting the pitch slightly); or 3) compose new or random notes. The usage of harmony ensures that the best harmonies will be carried over to the new harmony memory, which corresponds to the choice of the best-fit individuals. Pitch adjustment means to change the frequencies, which corresponds to generate a slightly different solution. The randomization is then used to increase the diversity of the solutions. The used parameters in our Harmony search method were *memory size* =100, *number of variables* =6, *memory considering rate* =0.8, *pitch adjusting rate* =0.3 and *bandwidth range* =0.5. The optimization was performed for 10000 iterations to increase the chance of reaching to the global optimality.

### Expression data source

The microarray compendium of *E. coli* was obtained from [27] and the Pearson correlation over all conditions in the compendium was used to calculate the gene pair co-expression.

### Visualizing detected modules

PPI networks inside each module were visualized by Cytoscape software [28]. To show the gene pairs co-expression, the value of 0.2132 was used as cut-off because 70% of co-complex protein pairs exhibited co-expression over this cut-off value. On the other hand, less than 5% of all gene pairs were above this cut-off. Gene pairs inside each module that exhibit higher co-expression than the mentioned threshold were linked by a solid line (see Fig. 4 and Additional file 1: Figures S1-S7) and visualized by Cytoscape software.

### Detecting central proteins in PPI modules

Central proteins interact with a large set of proteins inside a PPI module. We considered proteins with node degree larger than two times of average node degree of all constituting proteins of the modules as central proteins.

### Functional and gene essentiality analysis

EcoCyc database [13] was used for functional and gene essentiality analysis of central genes/proteins. Further literature mining was also performed using EcoCyc database. For the essentiality analysis, the data presented in [1] has considered as the main data source to detect essential genes in the main *E.coli* K-12 strain (substrain MG1655) in normal condition. In addition to this dataset, EcoCyc database provides data for other conditions and other strains for each gene from other studies.

## Results and discussion

### Constructing an integrated weighted PPI network and detecting modules

**Fig. 1:**
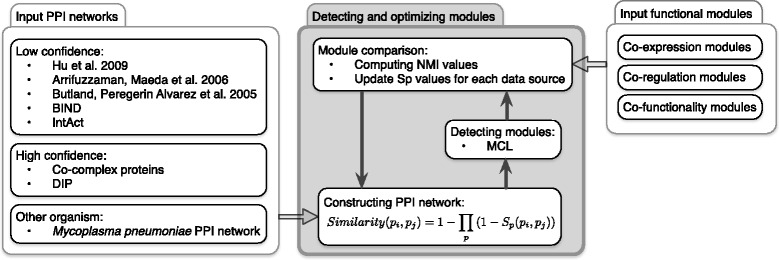
General protein complex prediction framework

In this section, we explain how we integrate different PPI datasets to construct a single weighted PPI network. First, we studied the average gene pairs co-expression of interacting proteins in each PPI dataset. Then we performed our proposed method to detect the weights of each dataset and integrate them through naïve Bayesian formula (Fig. 1). Finally, we checked the relations between gene pairs co-expression of interacting proteins in each dataset and their final weights.

**Fig. 2:**
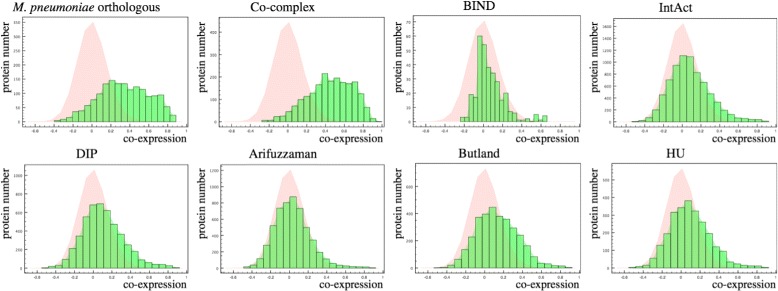
Co-expression of gene pairs in each data source. Co-expression of interacting pairs in each data source is shown as *green* histogram, and co-expression of all pairs is highlighted as a *pink* background

The average gene pair co-expression in each dataset is shown in Fig. 2. The known co-complex gene pairs exhibit the highest gene pairs co-expression, followed by orthologous proteins of *M. Pneumonia* high confidence interacting protein dataset. As it can be seen in Fig. 2, the average gene pairs co-expression in some of the PPI dataset (green bars) exhibit similar distribution pattern to the existing gene pairs co-expression shown as pink background. This means that these distributions are similar to a randomly chosen gene set. This is in contrast to the known co-complex proteins which exhibit much higher gene pair co-expression in comparison to a random set. Therefore, from the first step, we could expect lower weights in the naïve Bayesian formula for these PPI datasets with lower gene pairs co-expression.

**Fig. 3:**
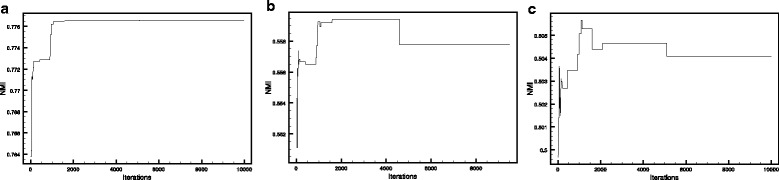
Optimization by harmonic search. **a** The PPI network integration parameters were optimized in a way that detected modules show the highest similarity to the co-expressed modules. Normalized Mutual information (NMI) values were used as a measure to compare similarities at the detected module level and optimization were performed for 10000 iterations. **b** The NMI values of the best detected module set at each iteration was compared with co-functionality modules using Gene Ontology terms. Although the co-expression module set as functional modules to optimize the NMI values, still the NMI values would increase in most of the interactions when the PPI modules was compared with the co-functionality modules. **c** The NMI values of the best detected module set at each iteration was also compared with co-regulation modules. Likewise, NMI values would fairly increase in the majority of iterations

As mentioned above, the protein datasets were integrated through naïve Bayesian formula. The weights were optimized by a global optimization algorithm referred to as Harmony search algorithm [29]. This algorithm was run for 10000 iterations. In each iteration, MCL clustering method was applied over this network and the result was compared to co-expression modules. Based on the similarity between the PPI and co-expression modules, measured by NMI, the weights were updated by Harmony search algorithm. The best NMI value in each iteration is shown in Fig. 3 a. Although the optimization was performed using the co-expression network, as the major functional relation network, the other functional networks such as co-functionality network derived from Gene Ontology terms and co-regulation network derived from the regulatory interactions could be used as alternative options (Fig. 3).

**Fig. 4:**
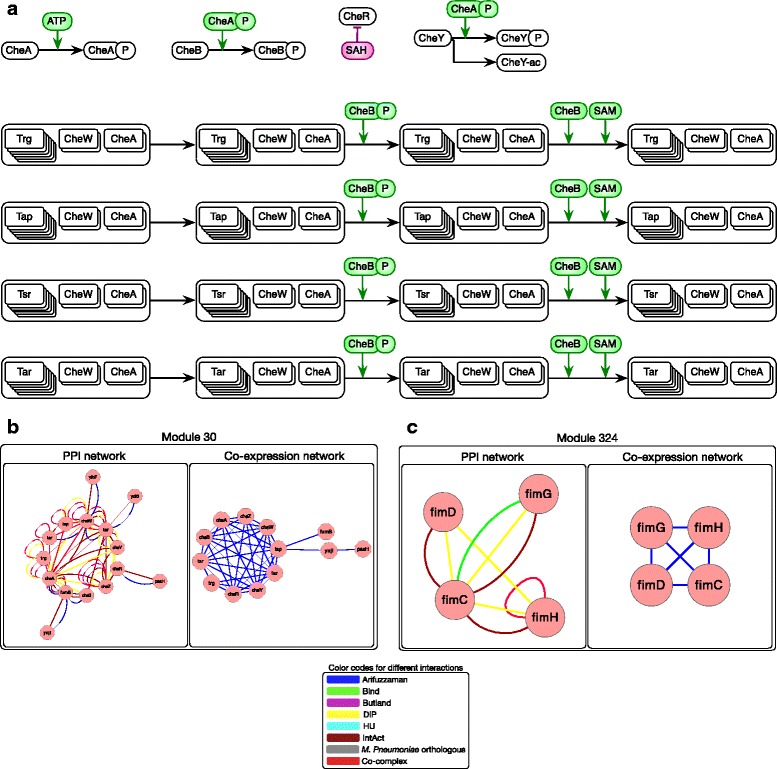
Predictions based on highly interacting modules. **a**, **b** Flagella signaling example. Predictions based on highly interacting modules. Module 30 consist of four chemotaxis signaling complexes: ribose/galactose/glucose sensing, dipeptide sensing, serine sensing, aspartate sensing. CheY, CheZ, CheR, CheB also interacts with either these complexes or CheA which is in the core of these complexes. **c** Pilus assembly example. In Module 324: FimC, FimD, FimG, and FimH are part of recently characterized Pilus assembly complex [30]

The final weights of different PPI datasets for naïve Bayesian formula are listed in Table 1. These weights for three PPI datasets (BIND, IntAct, and Arifuzzaman) have been clearly converged to zero. In contrast, three other PPI datasets (Hu, Butland, and *M. Pneumonia* orthologous) clearly show higher confidence level.

**Table 1:** Average co-expression and detected weight for each data source

Dataset	Number of	Average	Detected
	interactions	coexpression	weight
HU	5993	0.092	0.686
Ariffuzaman	11447	0.033	0.015
Butland	6227	0.133	0.653
IntAct	14437	0.081	0.005
BIND	487	0.068	0.036
DIP	10758	0.105	1
M. pneumoniae orthologous	3303	0.339	0.418
Co-complex	5541 ^a^	0.448	1

^a^All co-complex protein pairs have been considered

### Analyzing detected modules

Our proposed methodology not only determines the confidence level of the PPI datasets, but also generates high confidence modules similar to a given set of functional modules, which is co-expression modules here. To analyze modules, we studied a large number of PPI modules and complexes as well as their central/hub proteins. We also inspected how our result can accommodate recently published yeast two-hybrid PPI predictions [12].

Most of the detected PPI modules contain a highly connected central protein. We picked a measure (introduced in Materials and Methods) to detect these central/hub proteins. Additional file 1: Table S1 summarizes these proteins and their functions. Functional studies revealed that the majority of these hub proteins have certain functions. They are chaperone, ribosomal, and membrane hub proteins, or they are involved in kinase activity and RNA synthesis. The essentiality of the hub proteins were also more related to the function than the number of connections. For example, chaperone and ribosomal hub proteins which are involved in RNA synthesis are more likely to be essential genes at genome level. This is not the case for membrane hub proteins as well as hub proteins with kinase activity.

We also studied several major large complexes such as Ribosome, RNA polymerase, DNA polymerase III, and primosomal complexes, as well as some interlaying membrane complexes. As the co-complex proteins have been connected by higher weights in the integrated PPI network, our prior expectation was to find all the large complexes or interlaying complexes in a single module, but it was not the case. Although most of the proteins constituting a certain protein complex were detected in the same module (Additional file 1: Table S2), proteins of large complexes were detected in more than one module. Ribosomal proteins were detected in four different modules along with elongation factors, degradosome proteins, some RNA modifications and synthesis proteins, and some cell division/cytoskeleton proteins (Additional file 1: Figure S1). Co-complex literature mining revealed that the majority of these proteins and protein complexes interact with both ribosome and each other. This is the reason why the ribosome complex was not detected in a single module. All the constituting proteins in other complexes such as RNA polymerase (Additional file 1: Figure S2), DNA polymerase III (Additional file 1: Figure S3), and primosome complex (Additional file 1: Figure S4) were not detected in the same PPI module because of the same reason. Using gene pair co-expression, as an external data source, can lead to more accurate prediction in RNA polymerase, DNA polymerase III, and primosome complex because the proteins which constitute these complexes exhibit high pair-wise gene co-expression. In the case of ribosomal proteins, even non-ribosomal proteins show high gene pair co-expression, and in this case even expression data source cannot help to predict the exact set of proteins constituting the complex. Similar to large protein complexes, we also expected that overlapping protein complexes had to be found in the same module. Therefore, we inspected two examples of highly interacting outer membrane proteins TolC and BtuB (Additional file 1: Figure S5). These proteins were connected to several protein complexes and these complexes were found in one large module in these two examples.

Although detecting meaningful modules in PPI network is challenging, we could highlight some new predictions which could be further validated by functional study and literature mining. Flagellum complex was found in the large module (Module 6 in Additional file 1: Figure S6) but this is not the only complex related to Flagella synthesis and motility. Four chemotaxis signaling complexes: ribose/galactose/glucose sensing, dipeptide sensing, serine sensing, and aspartate sensing were detected in module 30 (Fig. 4). Two proteins CheA and CheW are in the core of these four complexes. Co-complex literature mining revealed that CheY, CheZ, CheR, and CheB also interact with either these complexes or CheA. Additional file 1: Figure S6 includes other detected modules with one or more protein complex(es) as their core(s). The interacting genes with these protein complexes are potential predictions for complex expansion. We could also detect modules which are potentially new protein complexes (Additional file 1: Figure S7). Recently published Pilus assembly complex [30] is among these predictions. In addition to the mentioned functionally relevant PPI modules, some protein sets, exhibiting co-expression, that may have related biological functions are summarized in Additional file 1: Table S4.

Most recently published Y2H PPI predictions [12] were also compared with the detected modules as well as other available datasets. We could annotate 2048 PPI pairs in these datasets. Out of 2048 pairs, only 467 interactions have been identified in the used datasets of this study. In total, 575 interactions (28%) in Y2H dataset were either in other employed datasets or co-exists in detected PPI modules. By including the Y2H interactions which contain one of the detected central/hub proteins of this study, this number reaches 707 interactions (34%). Additional file 1: Table S3 summarizes the obtained results for Y2H interactions. Therefore, most of the interactions of Y2H data are not included in previously publish datasets or they could not be predicted from them. The average co-expression among gene pairs constituting these interactions is just 0.0465 which means that these proteins are not co-expressed and probably this dataset would not be able to gain higher weight in the integration methodology that was introduced here. It is also mentioned in the original paper that the majority of the interacting proteins in this network do not constitute protein complexes [12].

## Conclusions

In this study, we introduced a new methodology to integrate the available PPI datasets into a weighted PPI network in such a way that the detected modules exhibit the highest similarity to predefined sets of modules. We used co-expressed module to show that direct functional modules detected from Gene Ontology terms could lead to similar results. New possible complexes were highlighted among the results.

The global optimization used in this study highlighted the more proper PPI datasets that can be used for predicting interacting proteins involved in the same biological process and protein complex. BIND and IntAct databases do not provide relevant information for this aim, while highly stable high confidence PPI from a relevant organism can provide highly relevant information as we have highlighted this fact using *M. pneumoniae* orthologous proteins in *E.coli*. The results for high-throughput datasets were varied since three datasets, namely AP-MS datasets in *E. coli*, Hu [1], and Butland [10], provide high confidence PPI interactions while this was not the case for Arifuzzaman [11] dataset. This has been recently reported that Hu and Butland PPI datasets contain much more interactions among components of the same complex in comparison to Arifuzzaman [11]. The recently published Y2H dataset [12] may not be a high confidence dataset for module detection based analysis as the interacting proteins in this dataset do not exhibit high co-expression and the original authors have reported that Y2H experiments include less stable and more transient interactions in comparison to AP-MS [12].

Clusterability of PPI networks is still a major problem. Identifying protein complexes and relevant functional modules from PPI networks is still a challenge and a wide range of algorithms have been developed for this aim (see the recent review [31]). Still the precisions of the predictions seem to be more related to the input data than the method itself. In a recent study, [32] several methods have been applied to three PPI networks of yeast, and it was shown that the precision of the prediction is highly sensitive to the input dataset. The lowest precision of prediction was derived using Y2H input dataset [32]. The same phenomenon has been reported in *E. coli* that the majority of Y2H predictions in this organism do not constitute protein complexes [12]. Integrating expression data with PPI networks seems to be a standard procedure to gain more accurate modules as co-complex proteins are highly co-expressed. In this study, we have highlighted some protein complexes such as ribosome, degradosome, and elongation factor that are highly interacting and co-expressed with several other proteins. In this case, even integrating expression data cannot solve the clusterability problem. The best way to gain reliable module seems to be using more stable interactions as the input dataset. A recent study has proposed a new methodology to gain more accurate interactions dataset for protein complex retrieval [33].

Large set of AP-MS and Y2H data sources will become available in near future. Based on this study, we propose that more stable interactions from AP-MS experiments provide a reliable dataset for module detection and protein complex prediction studies like this. On the other hand, less stable AP-MS and Y2H interactions provide a better dataset for studying hub proteins, post-translational modifications, protein chaperoning, and connections between different protein complexes. Integrating expression and functional dataset as well as PPI networks from related organisms is more useful for stable protein complex detection studies than more transient interactions studies such as post-translation modification studies. The reason is that the transient interactions may not remain conserved in the evolution and genes involved in these kinds of interactions may not exhibit high co-expression. On the other hand, more stable interactions and protein complexes are highly conserved in the evolution [34, 35] and the interacting proteins are highly co-expressed [35].

## Additional file

Additional file 1 The details of module detection procedure are presented. **Figure S1.** Ribosomal modules. **Figure S2.** RNA polymerase. **Figure S3.** DNA polymerase III. **Figure S4.** Primosome complex. **Figure S5.** Highly interacting outer membrane examples TolC and BtuB. **Figure S6.** Detected modules with a single complex as their cores. **Figure S7.** Detected modules as possible new complexes. **Table S1.** Central proteins in detected modules. **Table S2.** Protein complexes constituting from more than one kind of protein (hetero-oligomer) and the detected PPI module(s) entailing their proteins. **Table S3.** Locus tag and gene id of detected protein interaction in Y2H data. **Table S4.** Co-expressed genes/proteins which are detected in the same PPI module and their biological functions. (PDF 1443 kb)

## Abbreviations

AP-MS: Affinity Purification followed by Mass Spectrometry
*E. coli*: *Escherichia coli*
*M. Pneumonia*: *Mycoplasma Pneumonia*
MCL: Markov Cluster
GO: Gene Ontology
NMI: Normalized Mutual Information
PPI: Protein-Protein Interaction
*Y2H*: *yeast two-hybrid*
