# Supplementary material for: Improving protein complex prediction by reconstructing a high-confidence protein-protein interaction network of Escherichia coli from different physical interaction data sources
Source: BMC Bioinformatics. 2017 Jan 3;18:10. doi: 10.1186/s12859-016-1422-x (PMC5209909; doi:10.1186/s12859-016-1422-x)
Supplement: Additional file 1 — The details of module detection procedure are presented. Figure S1. Ribosomal modules. Figure S2. RNA polymerase. Figure S3. DNA polymerase III. Figure S4. Primosome complex. Figure S5. Highly interacting outer membrane examples TolC and BtuB. Figure S6. Detected modules with a single complex as their cores. Figure S7. Detected modules as possible new complexes. Table S1. Central proteins in detected modules. Table S2. Protein complexes constituting from more than one kind of protein (hetero-oligomer) and the detected PPI module(s) entailing their proteins. Table S3. Locus tag and gene id of detected protein interaction in Y2H data. Table S4. Co-expressed genes/proteins which are detected in the same PPI module and their biological functions. (PDF 1443 kb) [file 12859_2016_1422_MOESM1_ESM.pdf]

SUPPLEMENTARY MATERIALS

Reconstructing a high-confidence protein-protein interaction network of Escherichia coli from different physical interaction data sources improves protein complex predictions

Shirin Taghipour, Peyman Zarrineh, Mohammad Ganjtabesh * and Abbas Nowzari-Dalini

Module detection

1 Detecting co-expression modules

Expression data was obtained from the microarray compendium of *E. coli* by Lemmens et al. [1]. For this aim, a weighted co-expression network was constructed. Then, Pearson correlations measured in the expression patterns of microarray experiments are taken as the weights of undirected connections between every pair of genes. By using 0.6 as a cut-off, an unweighted co-expression network was derived. OSLOM clustering method [2] was used to detect co-expression modules from this network.

2 Detecting co-functionality modules

Functional information as Gene Ontology (GO) terms for *E. coli* was downloaded from the AmiGO database [3]. Only biological process domain was used to define gene pairs similarity and built co-functionality network. To extract a functional similarity network, the GO terms associated to each gene are treated as binary variables: 1 if the gene participates in a given function and 0 otherwise. By considering all the functional terms and keeping an established order, a vector v_i per gene can be constructed detailing the functional information of gene i. The cosine similarity of two of these vectors v_i and v_j is used as a metric, accounting for genes i and j overlap in functionality:

$$Sim(g_i, g_j) = \frac{v_i.v_j}{||v_i||.||v_j||}$$

Here the size of vectors is equal to nonempty GO terms in *E. coli* (GO terms that at least one gene has assigned to it). Many genes are not well annotated or just annotated for very general function (GO term at top of the GO hierarchy), and their corresponding vectors contain many zeros. In this case, their similarity becomes 1 if another gene is also annotated for such general function, while the general functional classes are not very informative. For this reason, we used the dot product over the vectors without any normalization. In this way, those genes that are annotated for GO terms at lower GO hierarchy (more informative functional classes) are more likely to have higher similarity in co-functionality network.

A weighted co-functionality network was then built by assembling all the gene-gene dyadic similarities. OSLOM clustering method [2] was used to detect co-functionality modules from this network.

3 Detecting co-regulation modules

The *E. coli* regulatory network was downloaded from RegulonDB database [4]. First the PageRank value [5] of each regulator in the regulatory network was calculated as a measure to assess the importance of the regulator. Then, the co-regulation of all gene pairs were calculated by using the PageRank values of their common regulators. This co-regulation similarity for genes i and j is defined as follows:

$$Sim(g_i, g_j) = \sum_{t=1}^{n} \frac{regulation\_type(R_t)}{PageRank(R_t)},$$

where n is the number of common regulators between genes and $regulation\_type$ is equal to 1 if the common regulator R_t of genes g_i and g_j activates or suppresses both genes, and -1 otherwise. After constructing weighted co-regulation network, OSLOM clustering method [2] was used to detect co-regulation modules from this network. The full description of these networks construction can be found in: *https://sites.google.com/site/peymanzarrinehresearch/cross-net.*

*Correspondence: mgtabesh@ut.ac.ir
Full list of author information is available at the end of the article

References

1. Lemmens K, De Bie T, Dhollander T, De Keersmaecker SC, Thijs IM, Schoofs G and Marchal K. (2009). DISTILLER: a data integration framework to reveal condition dependency of complex regulons in Escherichia coli. *Genome Biol*, **10**, R27.

2. Lancichinetti A, Fortunato S and Kertész J. (2009). Detecting the overlapping and hierarchical community structure in complex networks. *New Journal of Physics*, **11**, 033015.
3. Carbon S, Ireland A, Mungall CJ, Shu S, Marshall B, Lewis S and *et al.* (2009) AmiGO: online access to ontology and annotation data, *Bioinformatics*, **25**,288-289.
4. Gama-Castro S, Salgado H, Santos-Zavaleta A, Ledezma-Tejeida D, Muñiz-Rascado L, García-Sotelo JS, Alquicira-Hernández K, Martínez-Flores I, Pannier L, Castro-Mondragón JA, Medina-Rivera A, Solano-Lira H, Bonavides-Martínez C, Pérez-Rueda E, Alquicira-Hernández S, Porrón-Sotelo L, López-Fuentes A, Hernández-Koutoucheva A, Moral-Chávez VD, Rinaldi F and Collado-Vides J. (2016). RegulonDB version 9.0: high-level integration of gene regulation, coexpression, motif clustering and beyond. *Nucleic Acids Res*, **44**, (D1): D133-43, doi: 10.1093/nar/gkv1156.
5. Brin S and Page L. (1998). The Anatomy of a Large-Scale Hypertextual Web Search Engine. *Seventh International World-Wide Web Conference (WWW 1998)*, April 14-18, Brisbane, Australia.

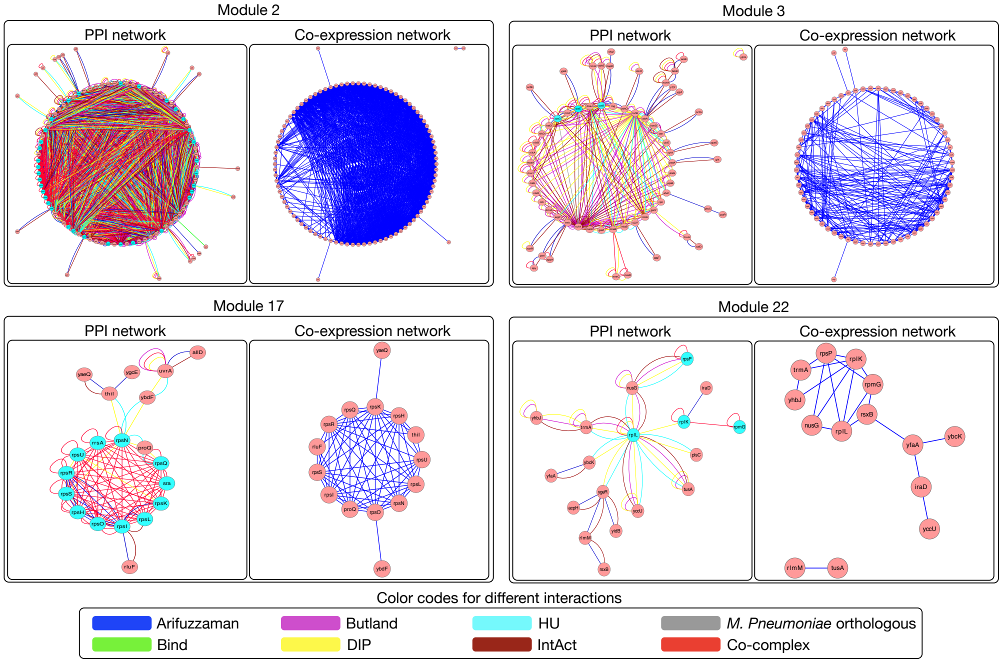

Figure S1 Ribosomal modules. Ribosomal complex is essential for RNA translation. Ribosomal proteins has been detected in four large PPI modules 2, 3, 17, and 22. Module 2 also include RNase P complex and Degradosome complex. Module 3 contains TU complex elongation faction. As it can be seen in this figure, many of the proteins and protein complexes which have found in these four modules are co-expressed with ribosomal proteins and our literature mining through EcoCyc database reveals that many of these proteins interacts with ribosome. Ribosomal proteins are marked with different color.

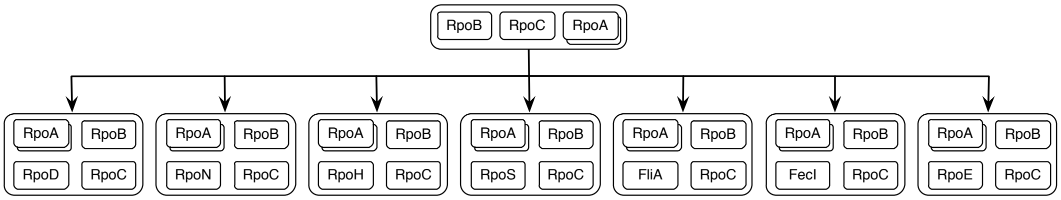

(a) RNA polymerase complexes derived from EcoCyc

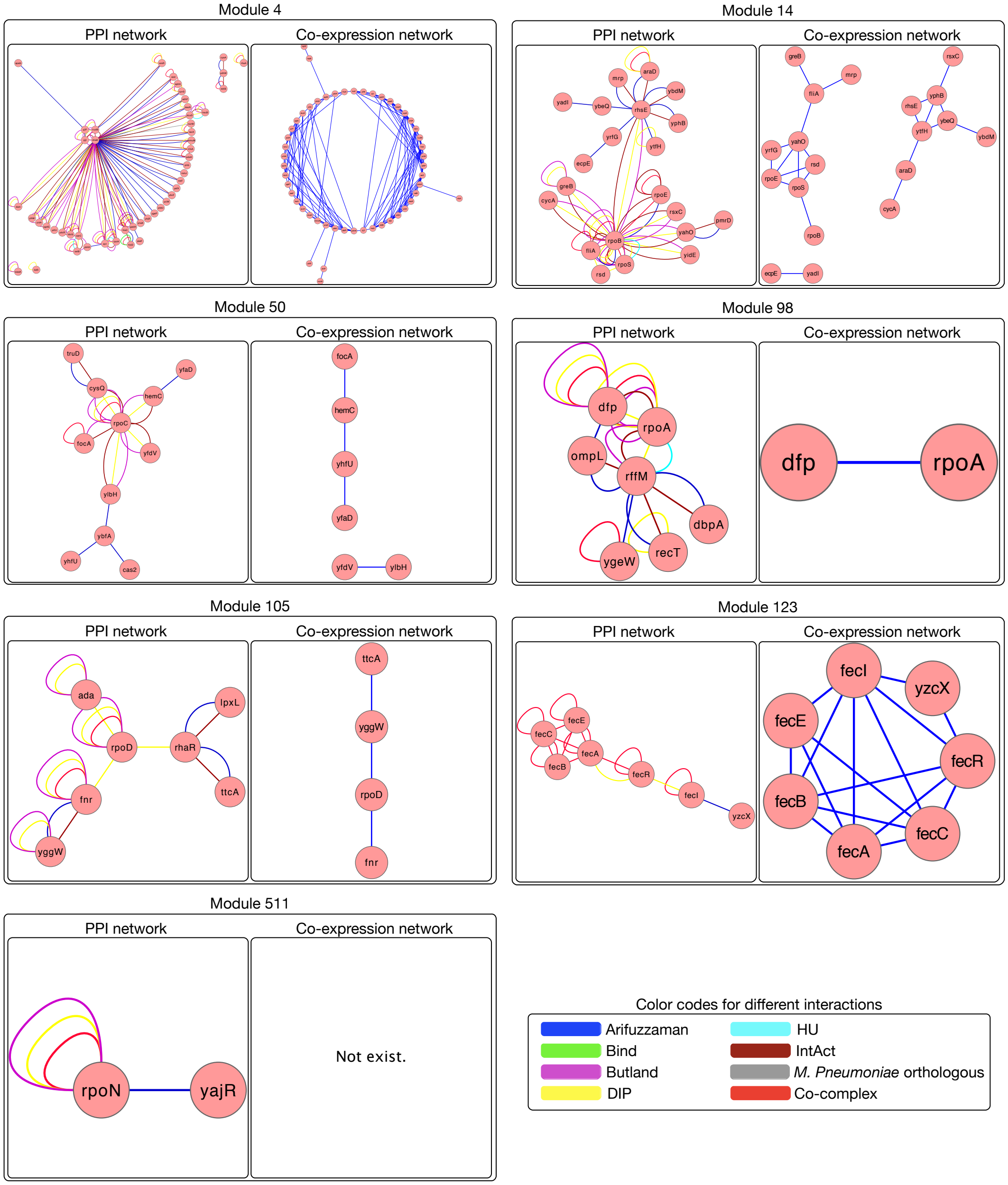

(b) Detected PPI modules containing RNA polymerase proteins

Figure S2 RNA polymerase. RNA polymerase is essential for initiation of gene transcription in Bacteria. **(a)** Seven different kinds of RNA polymerase complexes exist in *E. coli*. For the constitution all these seven complexes first [(RpoB)(RpoC)(RpoA)2] complex must be built. Each one of the seven RNA polymerase complexes contain a specific protein which interact with [(RpoB)(RpoC)(RpoA)2] complex. **(b)** RNA polymerase related proteins have been scattered in seven different modules (module 4, 14, 50, 98, 105, 123, 511).

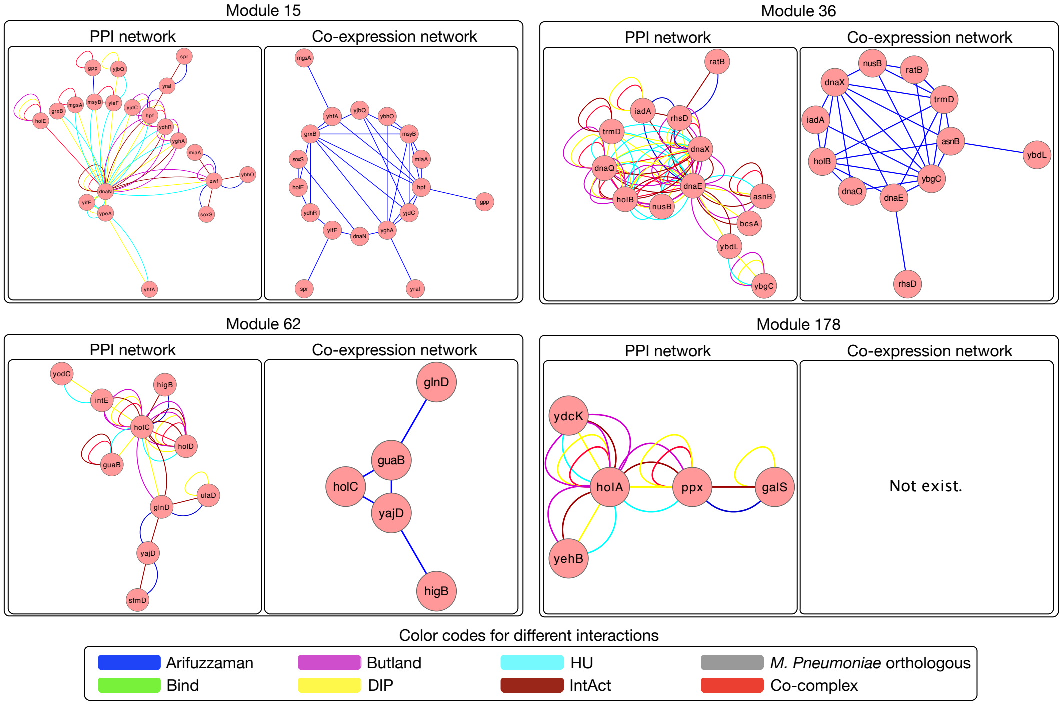

Figure S3 DNA polymerase III. DNA polymerse III complex is essential for DNA replication in bacteria. This large protein consist of ten different proteins: [(DnaE)(DnaQ)(HolE)]3[(DnaX)3(HolB)(HolA)][(DnaN)2]2[(DnaX)2][(HolC)(HolD)]4 DNA polymerase III related proteins have been scattered in four different modules (module 15, 36, 62, 178).

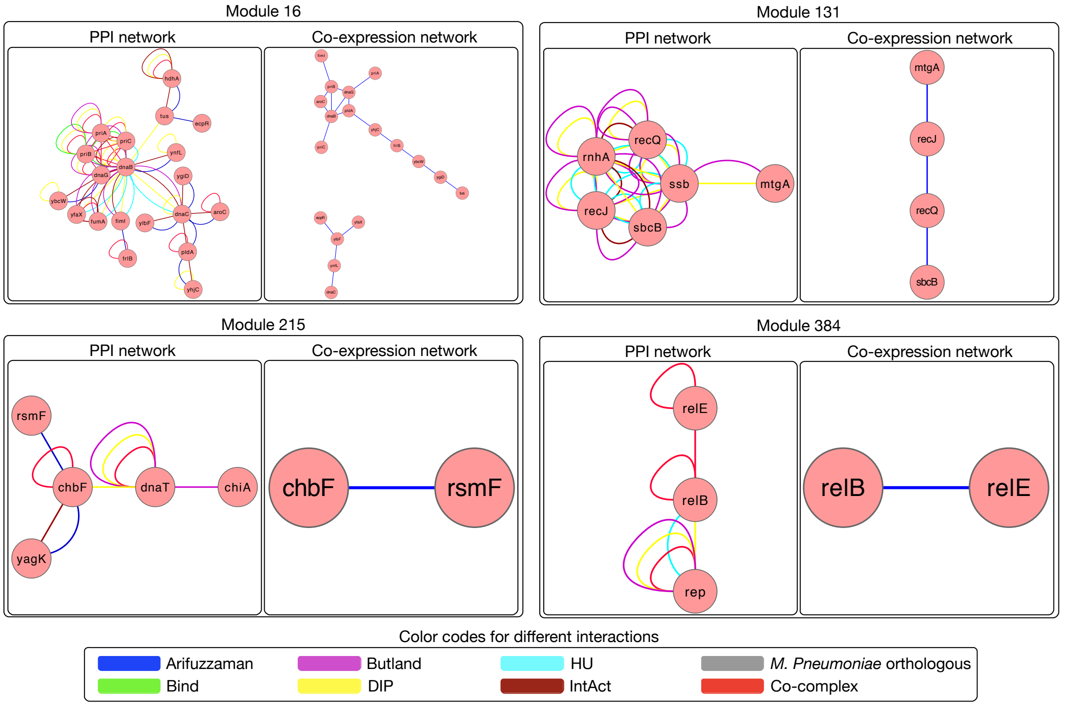

Figure S4 Primosome complex. Primosome complex is essential for DNA replication in bacteria. This complex consist of six proteins in *E. coli*: [(DnaB)6][(DnaT)3][(PriB)2][PriA][PriC][DnaG]. This complex has been detected in module 16 (Just DnaT is not in module 16, and has been found in module 215.) Interestingly DnaC has been also detected in module 16. Probably DnaC interacts with DnaB. DnaC is an accessory protein that loads the DnaB replicative helicase onto duplex DNA to initiate replication and onto single-stranded DNA to assist in primer formation by primase. DnaB is a component of another complex [(DnaB)6][(Rep)2][RecQ]. RecQ and Rep has been found in different modules 131 and 384.

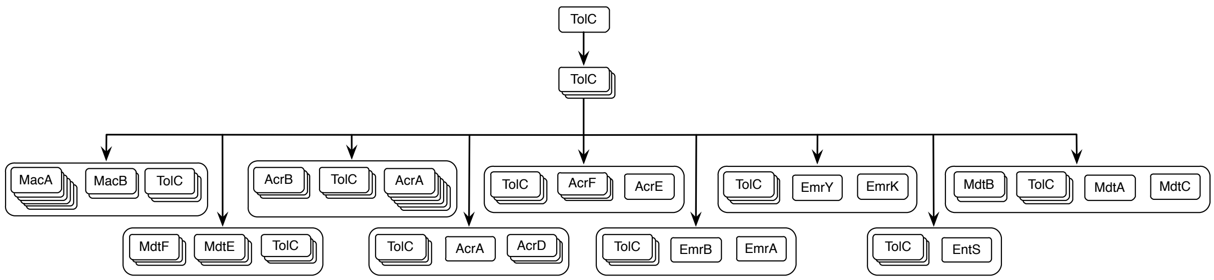

(a) TolC containing complexes derived from EcoCyc

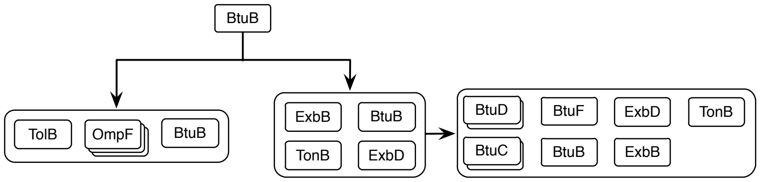

(b) BtuB containing complexes derived from EcoCyc

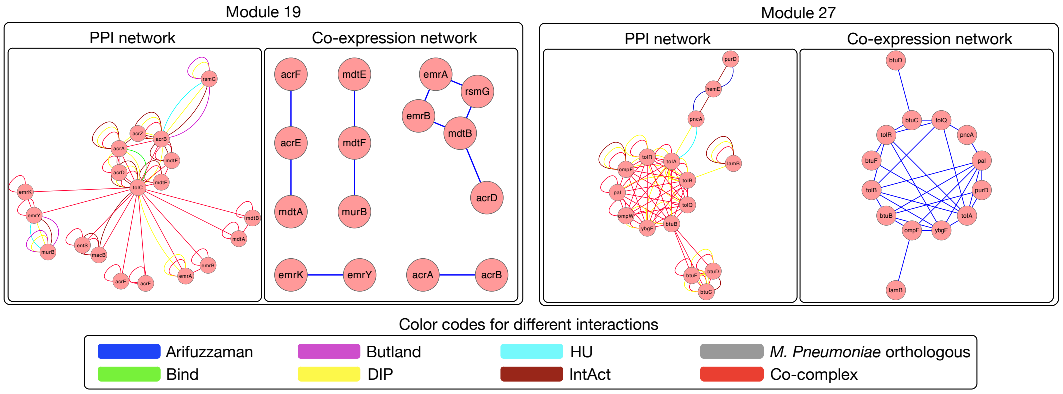

(c) Detected PPI modules containing TolC and BtuB

Figure S5 Highly interacting outer membrane examples TolC and BtuB. (a)(b) protein complexes include TolC and BtuB.**(c)** The detected modules containing TolC (module 19) and BtuB (module 27). In module 19, AcrZ is a small protein that interacts with the AcrAB-TolC multidrug efflux pump. The structure of this interaction can be found in Du et al 2014 (Du14).

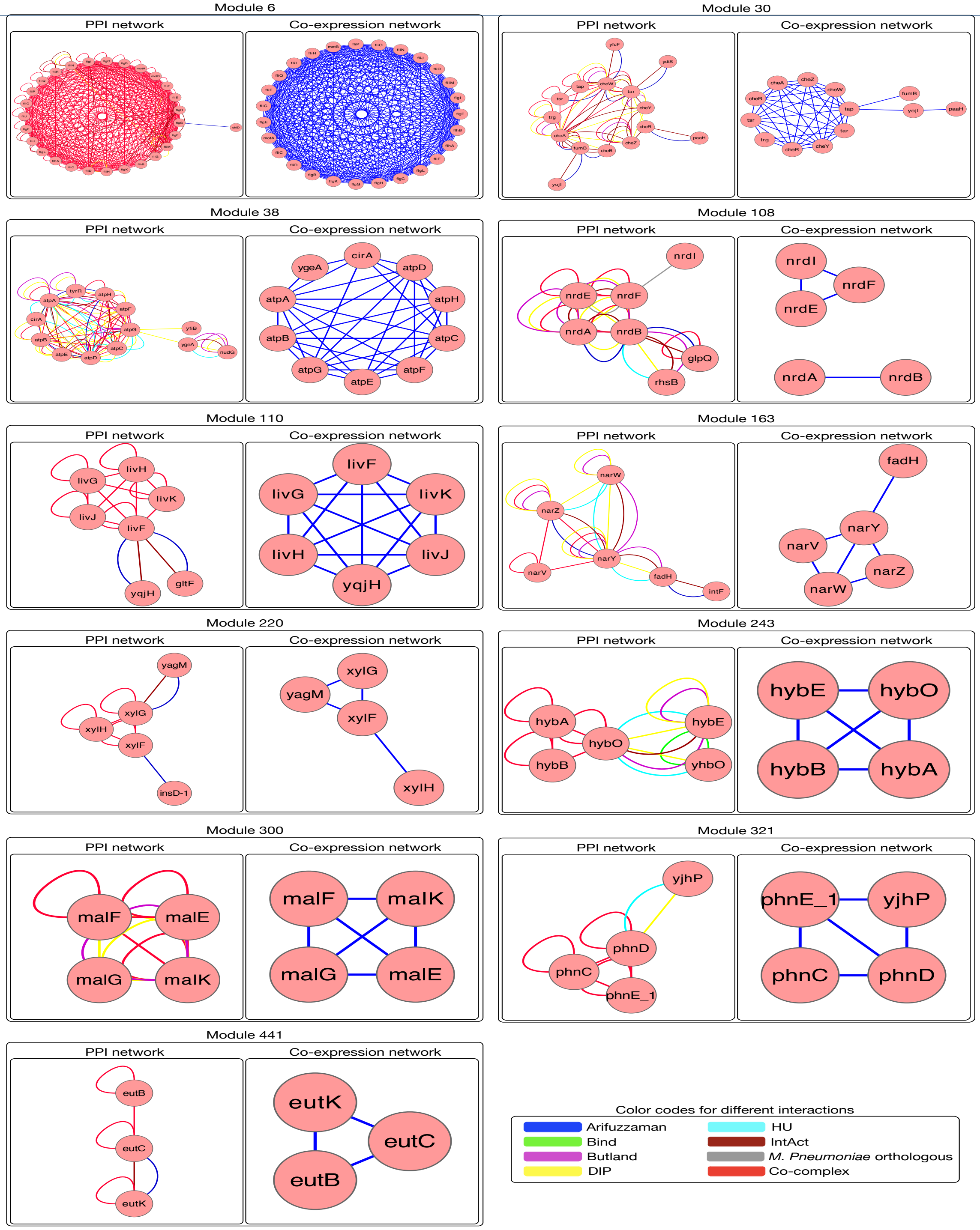

Figure S6 Detected modules with a single complex as their cores. Module 6 and 30 Flagella example: In module 6 yhiD does not co-expressed with other Flagellar proteins and not interact. In module 30, CheY, CheZ, CheR, CheB has flagella related function and are highly co-expressed with other flagella genes. They will probably interact and form complex. **Module 38 Transporter ATP synthase / thiamin triphosphate synthase complex:** Outer membrane protein CirA highly co-expressed with this complex and probably interacts with as predicted by Hu et al 2009. YgeA is another outer membrane protein which shows some co-expression with the complex and has potential to interact. **Module 108 ribonucleotide complexes:** This module contains two protein complexes [(NrdA)2(nrdB)2] and [(NrdE)2(nrdF)2]. These two homologous complexes are not co-expressed and probably never interact. nrdI gene is in the same orperon with nrdE and nrdeF genes (nrdHIEF operon). NrdI probably interacts [(NrdE) 2(nrdF)2] complex. **D. amino acid transport example:** LivFGHMJ and LivFGHMK are two ATP-dependent high-affinity branched-chain amino acid transport systems including two different complexes: [LivF][LivG][LivH][LivM][LivJ] and [LivK][LivM][LivH][LivG][LivF]. YqjH has potential to interact with these complexes as it also co-expressed with their genes. **Module 163 Transporter nitrate reductase Z complex:** Protein NarW probably interacts with [NarY][NarZ][NarV] complex as narW gene is in the same operon (NarZYWV operon) with other genes, constituting this protein complex. FadH has also potential to interact with the complex as previously predicted by Hu et al 2009. **Module 220 xylose ABC transporter complex:** YagM protein has the potential to interact with The complex [XylG]2[XylH]2[XylF] and also co-expressed with it. **Module 243 hydrogenase 2 complex.** hybE and hybO are in the same operon (hybOABCEFG operon) with genes constituting hydrogenase 2 complex [HybA][HybB][HybO][HybC], and probably interact with the complex. **Module 300 maltose ABC complex:** MalG is part of the complex [MalK]2[MalF][MalG][MalE]. **Module 321 phosphonate ABC transporter complex:** membrane protein YjhP has the potential to interact with the phosphonate ABC transporter complex [PhnC]2[$PhnE_1$]2[PhnD] as it also co-expressed with the complex constituting genes. **Module 441 ethanolamine ammonia-lyase complex:** EutK may interact with ethanolamine ammonia-lyase complex [EutB]6[EutC]6 as all genes are located in a single operon (eutBCLKR operon).

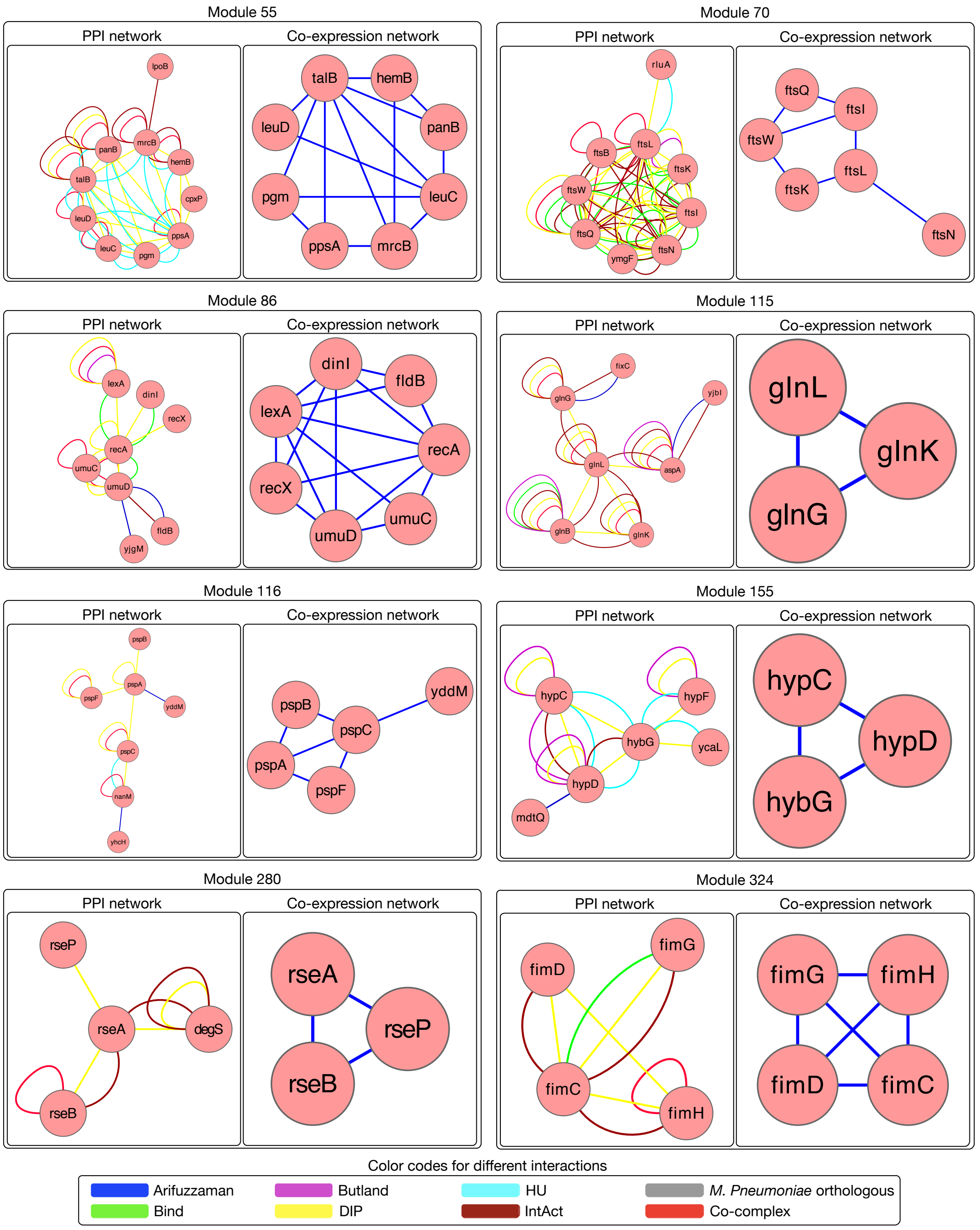

Figure S7 Detected modules as possible new complexes. Module 55: This module was previously predicted by Hu et al 2009. They predicted that highly co-expressed and interacting proteins PanB, MrcB, LeuD, TalB, PpsA, Pgm, LeuC can make a complex. We predict that HemB is part of this complex as it highly interact with other proteins and co-expressed with them. cpxP may also interacts although it does not show co-expression with other genes. **Module 70:** This Module consists of highly interacting and co-expressed proteins with the function related to cell division. **Module 86:** SOS response related proteins that are highly interacting, co-expressed, and regulated by LexA transcription factor. **Module 115:** Nitrogen fixation related proteins including transcriptions factor and kinase proteins. **Module 116:** Highly interacting phage shock proteins from psp operon. They probably form a complex. **Module 155:** Recently HypCD complex involved in hydrogenase maturation = [HypD][HypC] have been purified which is required for biosynthesis of Fe(CN)(2)CO cofactor. It is possible that more proteins interact in this module. A similar module has been predicted by Hu et al 2009. G. **Module 280:** From EcoCyc literature: RseB interacts RseA (the anti-sigma factor that inhibits sigma E). DegS and RseP catalyze proteolytic cleavage of RseA while RseB binds to the RseA and prevents DegS from cleaving RseA. **Module 324 Pilus assembly:** The complete description of the complex available in Literature Geibel et al 2013.

Table S1: **Central proteins in detected modules**. Module number of modules with central protein(s) are listed in the first column. The total number of protein in the module and the central proteins are written in the second and third columns, respectively. Number of connections of each central protein, function of the central protein and essentiality of the central protein are appeared in the fourth, fifth, and sixth column respectively. In the last column Y and N means essentiality and non-essentiality of the central proteins, respectively. The data derived from Baba et al 2006. Some central proteins marked as P in the last column, these proteins are either conditionally essential or essential some strains of *E. coli*. The partially essential proteins are deduced through literature mining from EcoCyc database.

**Module number**	**Total number of coding genes inside the module**	**Central protein(s)**	**Number of connection for central protein(s)**	**Function of the central protein(s)**	**Essentiality**
1	180	GroL	137	Chaperone protein	Y
1	180	Dld	5	Membrane Protein	N
2	99	RplA	58	Ribosomal protein	N
2	99	RplB	60	Ribosomal protein	Y
2	99	RplC	56	Ribosomal protein	Y
2	99	RplD	58	Ribosomal protein	Y
2	99	RplI	45	Ribosomal protein	N
2	99	RplM	54	Ribosomal protein	Y
2	99	RplO	43	Ribosomal protein	Y
2	99	rplP	42	Ribosomal protein	Y
2	99	RplQ	51	Ribosomal protein	Y
2	99	RplT	44	Ribosomal protein	Y
2	99	RplU	49	Ribosomal protein	Y
2	99	RplV	53	Ribosomal protein	Y
2	99	RplX	47	Ribosomal protein	Y
2	99	RpmB	56	Ribosomal protein	Y
2	99	RluB	44	RNA modification / 23S rRNA pseudouridine 2605 synthase	N
3	69	DnaJ	9	Chaperone protein	N
3	69	MreB	10	Cell division / Cytoskeleton protein	Y
3	69	RpsB	13	Ribosomal protein	Y
3	69	RpsE	10	Ribosomal protein	Y
3	69	RpsG	9	Ribosomal protein	Y
3	69	TufA	33	elongation factor Tu	-
3	69	TufB	32	elongation factor Tu	-
4	53	DnaK	34	Chaperone protein	N
5	49	NadE	33	de novo biosynthesis and salvage of NAD+ enzyme	Y
5	49	FusA	9	Elongation factor G	Y
7	27	SecA	18	Secretion protein	Y
7	27	SecB	10	Chaperone/secretion protein	N
8	25	CspC	10	RNA or single stranded DNA binding protein	N
8	25	RhlE	5	Ribosome-associated factor	N
9	25	RfaD	11	Membrane protein	N

Continued on next page

Table S1: **Central proteins in detected modules**. Module number of modules with central protein(s) are listed in the first column. The total number of protein in the module and the central proteins are written in the second and third columns, respectively. Number of connections of each central protein, function of the central protein and essentiality of the central protein are appeared in the fourth, fifth, and sixth column respectively. In the last column Y and N means essentiality and non-essentiality of the central proteins, respectively. The data derived from Baba et al 2006. Some central proteins marked as P in the last column, these proteins are either conditionally essential or essential some strains of *E. coli*. The partially essential proteins are deduced through literature mining from EcoCyc database. (Continued from previous page)

Module number	Total number of coding genes inside the module	Central protein(s)	Number of connection for central protein(s)	Function of the central protein(s)	Essentiality
9	25	RimJ	6	ribosomal-protein-S5-alanine N-acetyltransferase enzyme	N
10	25	GroS	16	Chaperone protein	Y
11	23	WbbK	10	Membrane protein	N
12	23	RcsB	12	Kinase activity	N
12	23	GfcD	6	Membrane protein	N
13	23	YbgI	6	Metal-binding protein	N
13	23	PdxB	4	Erythronate-4-phosphate dehydrogenase enzyme	P
13	23	YcgG	9	Membrane protein	N
14	21	RpoB	10	RNA polymerase	Y
14	21	RhsE	8	Self proteolysis	N
15	21	DnaN	12	DNA polymerase III	Y
16	20	DnaB	10	Primosome protein	Y
18	19	MetN	7	Membrane protein	N
18	19	YhjB	6	Kinase activity	N
19	19	TolC	15	Membrane protein	N
20	18	AcpP	10	interact with 12 different E. coli enzymes	Y
21	18	SlyD	10	Chaperone protein	P
21	18	UlaF	6	L-ribulose 5-phosphate 4-epimerase enzyme	N
22	18	RplL	8	Ribosomal protein	Y
22	18	YgeR	4	Membrane protein	N
23	18	FrdA	9	Membrane protein	N
23	18	HemG	6	Membrane protein	Y
24	17	GcvP	10	Glycine decarboxylase enzyme	N
25	17	MrcA	4	Cell division/ Membrane protein	N
25	17	OsmC	4	osmotically inducible peroxiredoxin enzyme	N
26	16	PurR	5	Transcription factor	N
26	16	KdgR	8	Transcription factor	N
28	16	YjtD	6	rRNA methyltransferase	N
28	16	ClpB	8	Chaperone protein	N
29	16	IbpA	8	Chaperone protein	N
30	15	CheA	9	Kinase activity	N

Continued on next page

Table S1: **Central proteins in detected modules**. Module number of modules with central protein(s) are listed in the first column. The total number of protein in the module and the central proteins are written in the second and third columns, respectively. Number of connections of each central protein, function of the central protein and essentiality of the central protein are appeared in the fourth, fifth, and sixth column respectively. In the last column Y and N means essentiality and non-essentiality of the central proteins, respectively. The data derived from Baba et al 2006. Some central proteins marked as P in the last column, these proteins are either conditionally essential or essential some strains of *E. coli*. The partially essential proteins are deduced through literature mining from EcoCyc database. (Continued from previous page)

**Module number**	**Total number of coding genes inside the module**	**Central protein(s)**	**Number of connection for central protein(s)**	**Function of the central protein(s)**	**Essentiality**
30	15	Tar	7	Kinase activity/Membrane protein	N
31	15	ProA	5	Kinase activity	P
31	15	SfsA	4	Transcription factor	N
31	15	YehT	5	Kinase activity	N
32	15	Tig	11	Chaperone protein	N
33	15	LysS	8	tRNA synthetase	N
33	15	RhaS	4	Transcription factor	N
34	14	NapC	12	Membrane protein	N
36	13	DnaE	10	DNA polymerase protein	Y
37	13	FkpB	8	Chaperone protein	N
37	13	IntA	9	CP4-57 prophage; integrase	N
40	13	YbhK	6	Predicted transferase with NAD(P)-binding Rossmann-fold domain	N
41	13	ParE	8	Topoisomerase protein	Y
42	12	PheA	6	Chorismate mutase / prephenate dehydratase enzyme	P
42	12	PrfA	5	Translation/ Release of the growing polypeptide chain at stop codons	Y
43	12	DsbA	9	Chaperone protein	N
44	12	SucC	5	Succinyl-CoA synthetase protein	N
45	12	YgaM	5	Membrane protein	N
46	11	SdhA	10	Membrane protein	N
47	11	YdhX	4	Membrane protein	N
48	11	DusC	6	tRNA synthetase	N
49	11	EutQ	4	Uncharacterized protein	N
49	11	IspA	5	Geranyl diphosphate synthase / farnesyl diphosphate synthase enzyme	Y
50	11	RpoC	5	RNA polymerase protein	Y
51	11	Crr	7	Kinase activity/ Membrane protein	P
53	10	SelD	4	Kinase activity	N
53	10	Rpe	4	Ribulose-5-phosphate 3-epimerase enzyme	N
54	10	PmbA	5	Chaperone protein	N
55	10	PpsA	8	Kinase activity	N
56	10	AceE	7	Membrane protein	N
57	10	TatE	7	Membrane protein	N

Continued on next page

Table S1: **Central proteins in detected modules**. Module number of modules with central protein(s) are listed in the first column. The total number of protein in the module and the central proteins are written in the second and third columns, respectively. Number of connections of each central protein, function of the central protein and essentiality of the central protein are appeared in the fourth, fifth, and sixth column respectively. In the last column Y and N means essentiality and non-essentiality of the central proteins, respectively. The data derived from Baba et al 2006. Some central proteins marked as P in the last column, these proteins are either conditionally essential or essential some strains of *E. coli*. The partially essential proteins are deduced through literature mining from EcoCyc database. (Continued from previous page)

Module number	Total number of coding genes inside the module	Central protein(s)	Number of connection for central protein(s)	Function of the central protein(s)	Essentiality
58	10	IscU	6	Chaperone protein	N
59	10	YdfG	7	3-hydroxy acid dehydrogenase enzyme	N
61	10	ClpP	8	proteases, cleavage of compounds/ Membrane protein	N
62	10	HolC	5	DNA polymerase III	N
63	10	ChbA	8	Phosphotransferase Systems (PEP-dependent PTS)	N
65	9	HslU	6	Chaperone protein	N
66	9	DeaD	4	Ribosomal protein	N
67	9	RibD	5	Fused diaminohydroxyphosphoribosylaminopyrimidine deaminase / 5-amino-6-(5-phosphoribosylamino)uracil reductase enzyme	Y
68	9	YdfZ	4	Uncharacterized protein	N
71	9	YgfK	5	Uncharacterized protein	N
72	9	HsdM	5	Methylation	N
73	9	Atl	4	DNA repair	N
74	9	GabT	5	4-aminobutyrate aminotransferase enzyme	N
75	9	LepA	4	elongation factor 4	N
75	9	PsuG	4	predicted pseudouridine 5'-phosphate glycosidase	N
76	8	LysU	5	tRNA synthetase	N
77	8	PepD	7	proteolysis	N
79	8	YghW	6	Uncharacterized protein	N
81	8	BluR	4	Transcription factor	N
82	8	CusR	4	Kinase activity	N
82	8	CusS	4	Kinase activity	N
83	8	LpxD	5	UDP-3-O-(R-3-hydroxymyristoyl)-glucosamine N-acyltransferase enzyme	Y
85	8	YkfJ	4	Uncharacterized protein	N
86	8	RecA	5	SOS response	N
88	8	FolA	5	dihydrofolate reductase enzyme	Y
90	8	TypA	6	Ribosomal protein	N
91	8	MazE	5	toxin-antitoxin system	N

Continued on next page

Table S1: **Central proteins in detected modules**. Module number of modules with central protein(s) are listed in the first column. The total number of protein in the module and the central proteins are written in the second and third columns, respectively. Number of connections of each central protein, function of the central protein and essentiality of the central protein are appeared in the fourth, fifth, and sixth column respectively. In the last column Y and N means essentiality and non-essentiality of the central proteins, respectively. The data derived from Baba et al 2006. Some central proteins marked as P in the last column, these proteins are either conditionally essential or essential some strains of *E. coli*. The partially essential proteins are deduced through literature mining from EcoCyc database. (Continued from previous page)

**Module number**	**Total number of coding genes inside the module**	**Central protein(s)**	**Number of connection for central protein(s)**	**Function of the central protein(s)**	**Essentiality**
93	8	RsfS	4	ribosomal silencing factor	N
94	7	GalF	4	redicted uridylyltransferase subunit with GalU	N
95	7	TopB	4	DNA topoisomerase III	N
96	7	HtpG	5	Chaperone protein	N
98	7	RffM	5	surface antigens	N
99	7	RlmN	6	rRNA methyltransferase/tRNA methyltransferase	N
100	7	UlaR	4	Transcription factor	N
103	7	ThrS	4	tRNA synthetase	Y
104	7	GltA	4	citrate synthase enzyme	P
106	7	YfbN	4	Uncharacterized protein	N
109	7	AllE	4	S-ureidoglycine aminohydrolase enzyme	N
111	7	DhaM	4	Kinase activity	N
112	7	MtlD	5	mannitol-1-phosphate 5-dehydrogenase enzyme	N
113	7	YebC	5	Uncharacterized protein	N
116	7	PspA	4	Prophage and phage related	N
118	7	TrpA	4	tryptophan synthase enzyme	P
119	7	OmpR	4	Transcription factor	N
120	7	TalA	5	transaldolase A enzyme	N
121	7	DapD	6	tetrahydrodipicolinate succinylase enzyme	Y
122	7	fau	5	Ligase activity	N
124	6	YhcC	4	Uncharacterized protein	N
128	6	YibA	4	predicted lyase containing HEAT-repeat	N
132	6	SerS	4	tRNA synthetase	Y
136	6	GlpD	5	Membrane protein	P
137	6	IscS	4	tRNa synthetase	P
141	6	YmfD	4	Uncharacterized protein	N
144	6	YggD	5	Uncharacterized protein	N
149	6	YaiA	4	Uncharacterized protein	N
151	6	TrpE	5	Lyase activity	P
154	6	RfbD	4	surface antigens	N

Continued on next page

Table S1: **Central proteins in detected modules**. Module number of modules with central protein(s) are listed in the first column. The total number of protein in the module and the central proteins are written in the second and third columns, respectively. Number of connections of each central protein, function of the central protein and essentiality of the central protein are appeared in the fourth, fifth, and sixth column respectively. In the last column Y and N means essentiality and non-essentiality of the central proteins, respectively. The data derived from Baba et al 2006. Some central proteins marked as P in the last column, these proteins are either conditionally essential or essential some strains of *E. coli*. The partially essential proteins are deduced through literature mining from EcoCyc database. (Continued from previous page)

**Module number**	**Total number of coding genes inside the module**	**Central protein(s)**	**Number of connection for central protein(s)**	**Function of the central protein(s)**	**Essentiality**
156	6	PrmA	5	Methylation	N
157	6	BglG	4	Kinase activity	N
159	6	FtsE	5	Membrane protein	Y
160	6	YciA	5	acyl-CoA thioesterase enzyme	N
161	6	MqsR	5	toxin-antitoxin system	N
166	6	YqjG	4	S-glutathionyl-(chloro)hydroquinone reductase enzyme	N
167	6	RsgA	5	Ribosomal protein	N
169	6	NrdD	5	ribonucleoside-triphosphate reductase enzyme	N
176	5	RplW	4	Ribosomal protein	Y
179	5	Blc	4	Membrane protein	N
181	5	YkgC	4	hypochlorite stress response	N
182	5	YciH	4	Ribosomal protein	N
186	5	MetL	3	Kinase activity	P
190	5	YbaB	4	DNA-binding protein	N
191	5	YccS	4	Membrane protein	N
192	5	KdsB	4	3-deoxy-D-manno-octulosonate cytidylyltransferase enzyme	Y
202	5	GlmS	4	L-glutamine:D-fructose-6-phosphate aminotransferase enzyme	Y
205	5	HyaE	4	Chaperone protein	N
219	5	RcbA	4	Prophage and phage related	N
224	5	RarA	4	recombination factor	N
226	5	TtdR	4	Transcription factor	N
233	5	FklB	4	Chaperone protein	N
234	5	RluD	4	Ribosomal protein	N
235	5	YoaH	4	Uncharacterized protein	N
240	5	EutD	4	phosphate acetyltransferase enzyme	N

Table S2: **Protein complexes constituting from more than one kind of protein (hetero-oligomer) and the detected PPI module(s) entailing their proteins.**

Protein Complex	Detected PPI Module
γ-glutamyl kinase-GP-reductase multienzyme complex	31
2,3-diketo-L-gulonate:Na<*sup*>+</*sup*> symporter	604
2-dehydro-3-deoxyphosphoheptonate aldolase	1
2-oxoglutarate dehydrogenase complex	269, 78
3-phenylpropionate dioxygenase system	327
4-amino-4-deoxychorismate synthase	143
50S ribosomal protein complex L8	2, 22
<I>Eco</I>KI restriction-modification system	72
<I>N</I>-acetyl-D-galactosamine PTS transporter	496
<i>p</i>-aminobenzoyl-glutamate hydrolase	472
<i>para</i>-aminobenzoate synthase multi-enzyme complex	143
acetoacetyl-CoA transferase	399
acetohydroxybutanoate synthase / acetolactate synthase	170
acetolactate synthase / acetohydroxybutanoate synthase	326, 492
acetyl-CoA carboxylase	2, 343, 65
acetyl-CoA carboxyltransferase	2, 343
AcrAB-TolC multidrug efflux transport system	19
AcrAD-TolC multidrug efflux transport system	19
AcrEF-TolC multidrug efflux transport system	19
aerobic fatty acid oxidation complex	262
aldehyde dehydrogenase	325
aliphatic sulfonate ABC transporter	467
alkylhydroperoxide reductase	259, 200
anaerobic fatty acid oxidation complex	180
anaerobic nucleoside-triphosphate reductase activating system	169
anthranilate synthase	151
arabinose ABC transporter	385
arginine ABC transporter	87
aspartate kinase / homoserine dehydrogenase	186
aspartate transcarbamylase	578
ATP synthase	38
ATP synthase F₀ complex	38
ATP synthase F₁ complex	38
autoinducer-2 ABC transporter	125
bifunctional CysEK cysteine biosynthesis complex	538
branched chain amino acid ABC transporter	110
capsular polysaccharide export apparatus	583
carbamoyl phosphate synthetase	51, 225
CcmCDE protoheme IX reservoir complex	60
CcmEFGH holocytochrome <I>c</I> synthetase	60
chemotaxis signaling complex - aspartate sensing	30
chemotaxis signaling complex - dipeptide sensing	30
chemotaxis signaling complex - ribose/galactose/glucose sensing	30
chemotaxis signaling complex - serine sensing	30
chitobiose / cellobiose PTS permease	63
ChpB-ChpS complex	524

Continued on next page

Table S2: **Protein complexes contituting from one kind of protein, and the detected PPI module(s) entailing their proteins (Continued from previous page)**

Protein Complex	Detected PPI Module
citrate lyase, inactive	211
ClpAP	61, 296
ClpAXP	61, 296, 25
ClpXP	61, 25
Colicin S4 Transport System	27
complex involved in modification of tRNA	316, 139
connecting fragment of NADH dehydrogenase I	35
copper / silver efflux transport system	318
CRISPR-associated complex for antiviral defense	221
curli secretion and assembly complex	423
cytochrome <i>bd</i>-I terminal oxidase	425
cytochrome <i>bd</i>-II terminal oxidase	504
cytochrome <i>bo</i> terminal oxidase	272
D-allose ABC transporter	206
degradosome	87, 2, 259
dihydroxyacetone kinase	111, 543
dimethyl sulfoxide reductase	426
DinJ-YafQ antitoxin/toxin complex and DNA-binding transcriptional repressor	347
dipeptide ABC transporter	294
DNA gyrase	195
DNA polymerase III, ψ-χ subunit	62
DNA polymerase III, core enzyme	36, 15
DNA polymerase III, holoenzyme	36, 15, 178, 62
DNA polymerase III, preinitiation complex	36, 178
DNA polymerase V	86
DnaJ/DnaK/GrpE	3, 4, 418
DosC-DosP complex	548
dTDP-L-rhamnose synthetase	154
EfeU/EfeO/EfeB ferrous iron transporter; cryptic	390
EmrAB-TolC multidrug efflux transport system	19
EmrKY-TolC multidrug efflux transport system	19
entericidin toxin-antitoxin complex	552
Enterobacterial Common Antigen Biosynthesis Protein Complex	319
enterobactin synthase multienzyme complex	359
EntS-TolC Enterobactin Efflux Transport System	19
ethanolamine ammonia-lyase	441
evolved β-D-galactosidase	551
exonuclease VII	189
FecAR Signal Transduction System	123
ferric citrate outer membrane transport complex	123, 273
ferric coprogen outer membrane transport complex	117, 273
ferric coprogen transport system	117
ferric dicitrate ABC transporter	123
ferric dicitrate transport system	123
ferric enterobactin ABC transporter	218
ferric enterobactin outer membrane transport complex	273, 218
ferric enterobactin transport system	218

Continued on next page

Table S2: **Protein complexes contituting from one kind of protein, and the detected PPI module(s) entailing their proteins (Continued from previous page)**

Protein Complex	Detected PPI Module
ferrichrome outer membrane transport complex	273, 117
ferrichrome transport system	117
fimbrial complex	411, 324
Flagellar Export Apparatus	6
Flagellar Motor Complex	6
Flagellar Motor Switch Complex	6
Flagellum	6
FlhDC DNA-binding transcriptional dual regulator	230
formate dehydrogenase-N	252
formate dehydrogenase-O	305
formate dependent nitrite reductase	171
formate hydrogenlyase complex	150
fructose PTS permease	563
FtsE/FtsX ABC transporter	159, 253
FtsLBQ cell division complex	70
fumarate reductase	23
GadE-RcsB DNA binding transcriptional activator	12
galactitol PTS permease	193
galactofuranose / galactopyranose ABC transporter	127
galactosamine PTS permease - cryptic	146
galactose ABC transporter	456
GatR DNA-binding transcriptional repressor	567
glucitol/sorbitol PTS permease	468
glucose PTS permease	403, 51
glutamate / aspartate ABC transporter	303
glutamate synthase	396
glutamine ABC transporter	270
glutathione / L-cysteine efflux transporter CydDC	236
glutathione ABC transporter	332
glycerol-3-phosphate / glycerol-2-phosphate ABC transporter	338, 624
glycerol-3-phosphate-dehydrogenase, anaerobic	288
glycine betaine / proline ABC transporter	312
glycine cleavage system	78, 24
glycolate oxidase	394
glycyl-tRNA synthetase	195, 29
GroEL-GroES chaperonin complex	1, 10
Grx4-BolA complex	404
GspC-O secreton complex	460
HflB, integral membrane ATP-dependent zinc metallopeptidase	361
HipB-HipA antitoxin/toxin complex and DNA-binding transcriptional repressor	81
histidine ABC transporter	89
HslVU protease	65, 129
HU DNA-binding transcriptional dual regulator	362, 274
hydrogenase 1	383
hydrogenase 2	243, 623
hydrogenase 3	150

Continued on next page

Table S2: **Protein complexes contituting from one kind of protein, and the detected PPI module(s) entailing their proteins (Continued from previous page)**

Protein Complex	Detected PPI Module
hydrogenase 4	39
hydroxylated aromatic carboxylic acid efflux transporter	124
HypA-HypB heterodimer	530
IHF DNA-binding transcriptional dual regulator	362, 461
imidazole glycerol phosphate synthase	570
iron (III) hydroxamate ABC transporter	117
isopropylmalate isomerase	55
K⁺ transporting ATPase	174
L-ascorbate PTS permease	271
L-methionine / D-methionine ABC transporter	18, 152
L-tartrate dehydratase	330
L-valine efflux transporter	600
leucine ABC transporter	110
LolCDE ABC lipoprotein transporter	406
longitudinal peptidoglycan synthesis/chromosome segregation-directing complex	3
LptABCFG ABC transporter	172
lysine / arginine / ornithine ABC transporter	89
MacAB-TolC macrolide efflux transport system	19, 400
malate dehydrogenase	298
MalT-MalK	493, 300
maltose / glucose PTS permease	51
maltose ABC transporter	300
mannitol PTS permease - cryptic	419
mannose PTS permease	429
MazE-MazF antitoxin/toxin complex and DNA-binding transcriptional repressor	91
McrBC restriction endonuclease	532
MdtABC-TolC multidrug efflux transport system	19
MdtEF-TolC multidrug efflux transport system	19
methylphosphonate degradation complex	52
molybdate ABC transporter	372
molybdopterin synthase	196
MqsA-MqsR antitoxin/toxin complex and DNA-binding transcriptional repressor	161
MukBEF complex	251
MukEF complex	251
murein tripeptide ABC transporter OppBCDFMppA	445
MutHLS complex, methyl-directed mismatch repair	528, 216
N-acetylmuramic acid PTS permease	51
NADH:ubiquinone oxidoreductase I	35
nickel ABC transporter	3
nitrate reductase A	92
nitrate reductase Z	163
nitrite reductase	590
NusB-NusE complex	488, 36
outer membrane lipopolysaccharide transport and assembly complex	208

Continued on next page

Table S2: **Protein complexes contituting from one kind of protein, and the detected PPI module(s) entailing their proteins (Continued from previous page)**

Protein Complex	Detected PPI Module
Outer Membrane Protein Assembly Complex	142
peptide ABC transporter OppABCDF	445, 434
peptide ABC transporter SapABCDF	299
peptide ABC transporter YejABEF	434
periplasmic nitrate reductase	34
phenylalanyl-tRNA synthetase	405, 291
phosphate ABC transporter	307
phospholipid ABC transporter	239
phosphonate ABC transporter	321
predicted carbon-phosphorous lyase complex	52
predicted xanthine dehydrogenase	596, 339
primosome	16, 215
PrlF-YhaV antitoxin-toxin complex	320
protoheme IX ABC transporter	60
PTS permease - unknown specificity	64
putative selenate reductase	237
putrescine / spermidine ABC transporter	457
putrescine ABC transporter	84
pyridine nucleotide transhydrogenase	281
pyruvate dehydrogenase	78, 56
RcsAB DNA-binding transcriptional dual regulator	12, 2
RcsB-BglJ DNA-binding transcriptional activator	12
RecBCD	56
RecFOR complex	162
RelB-RelE antitoxin/toxin complex and DNA-binding transcriptional repressor	384
resolvasome	10
ribonucleoside diphosphate reductase 1	108
ribonucleoside-diphosphate reductase 2	108
ribose ABC transporter	335
ring 1,2-phenylacetyl-CoA epoxidase	199
RNA polymerase sigma 19	98, 50, 14, 123
RNA polymerase sigma 24	98, 50, 14
RNA polymerase sigma 28	14, 98, 50
RNA polymerase sigma 32	98, 50, 14, 4
RNA polymerase sigma 38	98, 50, 14
RNA polymerase sigma 54	98, 50, 14, 511
RNA polymerase sigma 70	98, 50, 14, 105
RNA polymerase, core enzyme	98, 50, 14
RnlA-RnlB toxin-antitoxin complex	356
SdsRQP multidrug efflux system	341
Sec Translocation Complex	7
SecD-SecF-YajC-YidC Secretion Complex	7
SecYEG translocase	7
soluble NADH dehydrogenase fragment	35
spermidine efflux transporter	581
succinate dehydrogenase	46
succinyl-CoA synthetase	44

Continued on next page

Table S2: **Protein complexes contituting from one kind of protein, and the detected PPI module(s) entailing their proteins (Continued from previous page)**

Protein Complex	Detected PPI Module
SufBC₂D Fe-S cluster scaffold complex	379
sulfate / thiosulfate / selenate / selenite ABC transporter CysAUWSbp	290
sulfate / thiosulfate / selenite / selenate ABC transporter CysAUWCysP	290
sulfate adenylyltransferase	427
sulfite reductase	51
sulfur transfer protein complex	412
tagatose-1,6-bisphosphate aldolase 1	283
tagatose-1,6-bisphosphate aldolase 2	3
TatABCE protein export complex	57
taurine ABC transporter	459
The Colicin A Import System	27
The Tol-Pal Cell Envelope Complex	27
thiamin ABC transporter	114
thiazole synthase	1
TonB energy transducing system	273
topoisomerase IV	41, 355
translocation and assembly module	322
trehalose PTS permease	51
trimer complex of formate dehydrogenase-N α, β and γ subunits	252
trimethylamine N-oxide reductase I	463, 311
trimethylamine N-oxide reductase III	527, 588
tryptophan synthase	118
undecaprenyl-phosphate-α-L-Ara4N flippase	293
UvrABC Nucleotide Excision Repair Complex	344, 17, 480, 244
vitamin B12 outer membrane transport complex	27, 273
vitamin B12 transport system	27
vitamin B₁₂ ABC transporter	27
xanthine dehydrogenase	213
Xer site-specific recombination system	494
xylose ABC transporter	220
YadG/YadH ABC transporter	11
YbbA/YbbP ABC transporter	573
YbhF/YbhR/YbhS ABC transporter	350
YcjN/YcjO/YcjP ABC transporter	470
YdcS/YdcT/YdcV/YdcU ABC transporter	317
YddO/YddP/YddQ/YddR/YddS ABC transporter	207
YefM-YoeB antitoxin/toxin complex and DNA-binding transcriptional repressor	241
YehW/YehX/YehY/YehZ ABC transporter	302
YhdW/YhdX/YhdY/YhdZ ABC transporter	250
YnjC/YnjD ABC transporter	587
YphD/YphE/YphF ABC transporter	367
Zn²⁺ ABC transporter	257

Table S3: **Locus tag and gene id of detected protein interaction in Y2H data.** Locus tag and gene name of interacting proteins are listed in the first four columns. The existence of the interaction in other PPI networks is shown in the fifth column. The sixth and seventh column highlights whether the interacting proteins has been detected in a PPI module and whether one of the proteins is a central protein in a PPI module.

Locus tag1	Locus tag2	Gene1	Gene2	Existing in other data sources	Id of a detected module	Centeral protein
b0004	b0044	thrC	fixX	No	-	
b0008	b0071	talB	leuD	Yes	55	
b0029	b3262	ispH	yhdJ	No	1	
b0030	b3021	rihC	mqsA	No	-	
b0032	b2181	carA	yejG	No	-	
b0033	b0032	carB	carA	Yes	-	
b0037	b0037	caiC	caiC	No	158	
b0042	b0041	fixB	fixA	No	-	
b0042	b2988	fixB	gss	No	102	
b0042	b3910	fixB	yiiM	No	102	
b0048	b0044	folA	fixX	No	-	FolA
b0048	b1566	folA	flxA	No	-	FolA
b0048	b1575	folA	dicB	No	-	FolA
b0048	b0424	folA	yajL	No	-	FolA
b0048	b1831	folA	proQ	No	-	FolA
b0048	b3825	folA	pldB	No	-	FolA
b0049	b2022	apaH	hisB	Yes	25	
b0052	b0044	pdxA	fixX	Yes	331	
b0052	b1593	pdxA	ynfK	No	-	
b4659	b3560	yabP	glyQ	No	-	
b4659	b4181	yabP	yjfI	No	-	
b4659	b4379	yabP	yjjW	No	-	
b4659	b2714	yabP	ascG	No	-	
b0064	b3128	araC	garD	No	-	
b0071	b0071	leuD	leuD	Yes	55	
b0071	b0072	leuD	leuC	Yes	55	
b0077	b0077	ilvI	ilvI	Yes	326	
b0077	b3021	ilvI	mqsA	No	-	
b0078	b0077	ilvH	ilvI	Yes	-	
b0080	b4171	cra	miaA	No	-	
b0085	b4505	murE	insX	No	-	
b0085	b0328	murE	yahN	No	-	
b0085	b1366	murE	ydaY	Yes	-	
b0085	b2004	murE	cbeA	No	-	
b0085	b2980	murE	glcC	No	-	
b0085	b3488	murE	yhiJ	No	-	
b0086	b0086	murF	murF	Yes	334	
b0088	b0088	murD	murD	Yes	185	
b0088	b0095	murD	ftsZ	No	-	
b0088	b0096	murD	lpxC	No	-	
b0090	b2581	murG	yfiF	Yes	-	
b0091	b0084	murC	ftsI	No	-	

Continued on next page

Table S3: **Locus tag and gene id of detected protein interaction in Y2H data.** Locus tag and gene name of interacting proteins are listed in the first four columns. The existence of the interaction in other PPI networks is shown in the fifth column. The sixth and seventh column highlights whether the interacting proteins has been detected in a PPI module and whether one of the proteins is a central protein in a PPI module. (Continued from previous page)

Locus tag1	Locus tag2	Gene1	Gene2	Existing in other data sources	Id of a detected module	Centeral protein
b0091	b1351	murC	racC	No	-	
b0091	b1566	murC	flxA	No	-	
b0092	b2012	ddlB	yeeD	No	-	
b0093	b0095	ftsQ	ftsZ	No	-	
b0093	b1960	ftsQ	vsr	No	-	
b0093	b3933	ftsQ	ftsN	Yes	70	
b0093	b1391	ftsQ	paaD	No	-	
b0094	b3448	ftsA	yhhA	Yes	253	
b0099	b0099	mutT	mutT	Yes	473	
b0104	b0237	guaC	pepD	Yes	77	PepD
b0111	b3021	ampE	mqsA	No	-	
b0113	b4367	pdhR	fhuF	No	-	
b0115	b0116	aceF	lpd	Yes	78	
b0121	b3262	speE	yhdJ	No	-	
b0130	b3773	yadE	ilvY	No	-	
b0133	b2299	panC	yfcD	Yes	-	
b0145	b1831	dksA	proQ	No	-	
b0154	b2980	hemL	glcC	No	-	
b0154	b3021	hemL	mqsA	No	-	
b0159	b1916	mtn	sdiA	No	-	
b0166	b1566	dapD	flxA	No	-	DapD
b0171	b1566	pyrH	flxA	No	-	
b0172	b0172	frr	frr	Yes	11	
b0172	b1324	frr	tpx	No	11	
b0172	b2597	frr	raiA	No	11	
b0172	b2957	frr	ansB	No	11	
b0172	b4045	frr	yjbJ	No	11	
b0172	b1108	frr	ycfP	No	11	
b0182	b1566	lpxB	flxA	No	-	
b0182	b3021	lpxB	mqsA	No	-	
b0182	b3946	lpxB	fsaB	No	-	
b0182	b1105	lpxB	lpoB	No	-	
b0182	b3554	lpxB	yiaF	No	-	
b0182	b3010	lpxB	yqhC	No	-	
b0184	b0215	dnaE	dnaQ	Yes	36	DnaE
b0185	b0185	accA	accA	Yes	2	
b0185	b1126	accA	potA	No	-	
b0185	b2316	accA	accD	Yes	-	
b0188	b0188	tilS	tilS	No	12	
b0188	b1831	tilS	proQ	No	-	
b0200	b1462	gmhB	yddH	No	-	
b0200	b1717	gmhB	rpmI	No	-	

Continued on next page

Table S3: **Locus tag and gene id of detected protein interaction in Y2H data.** Locus tag and gene name of interacting proteins are listed in the first four columns. The existence of the interaction in other PPI networks is shown in the fifth column. The sixth and seventh column highlights whether the interacting proteins has been detected in a PPI module and whether one of the proteins is a central protein in a PPI module. (Continued from previous page)

Locus tag1	Locus tag2	Gene1	Gene2	Existing in other data sources	Id of a detected module	Centeral protein
b0200	b2696	gmhB	csrA	No	-	
b0215	b0215	dnaQ	dnaQ	Yes	36	
b0226	b0226	dinJ	dinJ	Yes	347	
b0237	b0237	pepD	pepD	Yes	77	PepD
b0240	b2741	crl	rpoS	No	-	
b0243	b0146	proA	sfsA	Yes	31	ProA, SfsA
b0243	b0243	proA	proA	Yes	31	ProA
b0243	b2422	proA	cysA	Yes	-	ProA
b0243	b2125	proA	yehT	Yes	31	ProA, YehT
b0261	b0044	mmuM	fixX	No	-	
b0261	b0199	mmuM	metN	No	-	MetN
b0261	b3043	mmuM	ygiL	No	-	
b0291	b2584	ecpC	pka	No	-	
b0325	b2012	yahK	yeeD	No	-	
b0337	b0337	codA	codA	Yes	229	
b0348	b0044	mhpB	fixX	No	-	
b0348	b0095	mhpB	ftsZ	No	-	
b0348	b0172	mhpB	frr	No	-	
b0348	b1156	mhpB	tfaE	No	-	
b0348	b2713	mhpB	hydN	No	-	
b0348	b4502	mhpB	yeiW	No	-	
b0348	b4342	mhpB	yjiT	No	-	
b0351	b0044	mhpF	fixX	No	-	
b0351	b0199	mhpF	metN	No	-	MetN
b0351	b4505	mhpF	insX	No	-	
b0351	b1176	mhpF	minC	No	-	
b0351	b2147	mhpF	preA	No	-	
b0351	b2907	mhpF	ubiH	No	-	
b0351	b2980	mhpF	glcC	No	-	
b0351	b2997	mhpF	hybO	No	-	
b0351	b0946	mhpF	zapC	No	-	
b0351	b4502	mhpF	yeiW	No	-	
b0351	b3262	mhpF	yhdJ	No	-	
b0351	[]	mhpF	[]	No	-	
b0351	b4342	mhpF	yjiT	No	-	
b0352	b1264	mhpE	trpE	No	-	TrpE
b0356	b0356	frmA	frmA	Yes	37	
b0381	b0095	ddlA	ftsZ	No	-	
b0386	b1831	proC	proQ	No	-	
b0387	b0044	yaiI	fixX	No	-	
b0387	b0618	yaiI	citC	No	-	
b0387	b1176	yaiI	minC	No	-	

Continued on next page

Table S3: **Locus tag and gene id of detected protein interaction in Y2H data.** Locus tag and gene name of interacting proteins are listed in the first four columns. The existence of the interaction in other PPI networks is shown in the fifth column. The sixth and seventh column highlights whether the interacting proteins has been detected in a PPI module and whether one of the proteins is a central protein in a PPI module. (Continued from previous page)

Locus tag1	Locus tag2	Gene1	Gene2	Existing in other data sources	Id of a detected module	Centeral protein
b0387	b1689	yaiI	ydiL	No	-	
b0387	b4379	yaiI	yjjW	No	-	
b0389	b0199	yaiA	metN	No	-	MetN, YaiA
b0389	b4505	yaiA	insX	No	-	YaiA
b0389	b1156	yaiA	tfaE	No	-	YaiA
b0389	b1176	yaiA	minC	No	-	YaiA
b0389	b2980	yaiA	glcC	No	-	YaiA
b0391	b0044	yaiE	fixX	No	-	
b0391	b2440	yaiE	eutC	No	-	
b0391	b3021	yaiE	mqsA	No	-	
b0391	b3043	yaiE	ygiL	No	-	
b0391	b4379	yaiE	yjjW	No	-	
b0391	b4587	yaiE	insN	No	-	
b0391	b1831	yaiE	proQ	No	-	
b0391	b4502	yaiE	yeiW	No	-	
b0391	b3871	yaiE	typA	No	-	TypA
b0399	b0321	phoB	yahG	No	-	
b0408	b1713	secD	pheT	Yes	-	
b0413	b0413	nrdR	nrdR	Yes	114	
b0414	b0044	ribD	fixX	No	-	RibD
b0414	b0414	ribD	ribD	Yes	67	RibD
b0414	b1566	ribD	flxA	No	-	RibD
b0414	b1575	ribD	dicB	No	-	RibD
b0417	b0223	thiL	yafJ	No	-	
b0417	b0398	thiL	sbcD	No	-	
b0417	b1566	thiL	flxA	No	-	
b0417	b1575	thiL	dicB	No	-	
b0417	b2012	thiL	yeeD	No	-	
b0417	b3043	thiL	ygiL	No	-	
b0417	b1361	thiL	ydaW	No	-	
b0421	b0421	ispA	ispA	Yes	49	IspA
b0421	b1849	ispA	purT	Yes	49	IspA
b0431	b0600	cyoB	ybdL	No	-	
b0437	b0437	clpP	clpP	Yes	61	ClpP
b0438	b0438	clpX	clpX	Yes	25	
b0440	b4367	hupB	fhuF	No	-	
b0456	b3825	ybaA	pldB	No	-	
b0463	b0463	acrA	acrA	Yes	19	
b0464	b0464	acrR	acrR	No	136	
b0467	b0560	priC	nohD	No	-	
b0467	b0775	priC	bioB	No	-	
b0467	b1394	priC	paaG	No	-	

Continued on next page

Table S3: **Locus tag and gene id of detected protein interaction in Y2H data.** Locus tag and gene name of interacting proteins are listed in the first four columns. The existence of the interaction in other PPI networks is shown in the fifth column. The sixth and seventh column highlights whether the interacting proteins has been detected in a PPI module and whether one of the proteins is a central protein in a PPI module. (Continued from previous page)

Locus tag1	Locus tag2	Gene1	Gene2	Existing in other data sources	Id of a detected module	Centeral protein
b0467	b2980	priC	glcC	No	-	
b0467	b3773	priC	ilvY	No	-	
b0467	b4052	priC	dnaB	Yes	16	DnaB
b0467	b0644	priC	ybeQ	No	-	
b0467	b1117	priC	lolD	No	-	
b0467	b2714	priC	ascG	No	-	
b0467	b3357	priC	crp	No	-	
b0470	b0184	dnaX	dnaE	Yes	36	DnaE
b0470	b0470	dnaX	dnaX	Yes	36	
b0470	b4259	dnaX	holC	Yes	-	HolC
b0470	b4372	dnaX	holD	Yes	-	
b0473	b0473	htpG	htpG	Yes	96	HtpG
b0473	b0946	htpG	zapC	Yes	96	HtpG
b0474	b0600	adk	ybdL	Yes	-	
b0475	b2440	hemH	eutC	No	-	
b0477	b0477	gsk	gsk	Yes	40	
b0490	b0029	ybbL	ispH	No	1	
b0490	b0490	ybbL	ybbL	Yes	1	
b0504	b0516	allS	allC	No	-	
b0506	b0037	allR	caiC	No	-	
b0506	b0506	allR	allR	Yes	284	
b0508	b0044	hyi	fixX	No	-	
b0508	b0199	hyi	metN	No	-	MetN
b0508	b0719	hyi	ybgD	No	-	
b0508	b2012	hyi	yeeD	No	-	
b0508	b3043	hyi	ygiL	No	-	
b0509	b0172	glxR	frr	No	-	
b0509	b0199	glxR	metN	No	-	MetN
b0509	b2012	glxR	yeeD	No	-	
b0509	b3043	glxR	ygiL	No	-	
b0525	b0700	ppiB	rhsC	Yes	201	
b0551	b4367	quuD	fhuF	No	-	
b0568	b0568	nfrA	nfrA	No	59	
b0586	b0044	entF	fixX	No	-	
b0586	b3936	entF	rpmE	No	-	
b0586	b4342	entF	yjiT	No	-	
b0588	b0311	fepC	betA	No	-	
b0588	b0588	fepC	fepC	Yes	218	
b0594	b0199	entE	metN	No	-	MetN
b0594	b0871	entE	poxB	No	-	
b0594	b1462	entE	yddH	No	-	
b0594	b1698	entE	ydiR	No	-	

Continued on next page

Table S3: **Locus tag and gene id of detected protein interaction in Y2H data.** Locus tag and gene name of interacting proteins are listed in the first four columns. The existence of the interaction in other PPI networks is shown in the fifth column. The sixth and seventh column highlights whether the interacting proteins has been detected in a PPI module and whether one of the proteins is a central protein in a PPI module. (Continued from previous page)

Locus tag1	Locus tag2	Gene1	Gene2	Existing in other data sources	Id of a detected module	Centeral protein
b0595	b2173	entB	yeiR	No	-	
b0595	b2980	entB	glcC	No	-	
b0595	b0946	entB	zapC	No	-	
b0596	b0172	entA	frr	No	-	
b0596	b0199	entA	metN	No	-	MetN
b0596	b2897	entA	cptB	No	-	
b0599	b0044	ybdH	fixX	No	-	
b0599	b0095	ybdH	ftsZ	No	-	
b0599	b0172	ybdH	frr	No	-	
b0599	b0599	ybdH	ybdH	No	439	
b0599	b1156	ybdH	tfaE	No	-	
b0599	b2713	ybdH	hydN	No	-	
b0599	b4502	ybdH	yeiW	No	-	
b0600	b0044	ybdL	fixX	No	-	
b0600	b0199	ybdL	metN	No	-	MetN
b0600	b0600	ybdL	ybdL	No	36	
b0600	b2242	ybdL	glpB	No	-	
b0600	b4502	ybdL	yeiW	No	-	
b0601	b0464	ybdM	acrR	No	-	
b0601	b0601	ybdM	ybdM	No	14	
b0601	b2713	ybdM	hydN	No	-	
b0601	b2869	ybdM	ygeV	No	-	
b0601	b0644	ybdM	ybeQ	No	14	
b0601	b0872	ybdM	hcr	No	-	
b0601	b2714	ybdM	ascG	No	-	
b0602	b0601	ybdN	ybdM	No	-	
b0603	b3262	ybdO	yhdJ	No	-	
b0605	b0172	ahpC	frr	No	-	
b0605	b0605	ahpC	ahpC	Yes	259	
b0605	b1096	ahpC	pabC	No	-	
b0610	b0199	rnk	metN	No	-	MetN
b0610	b0601	rnk	ybdM	No	-	
b0610	b0889	rnk	lrp	No	-	
b0610	b2980	rnk	glcC	No	-	
b0610	b3262	rnk	yhdJ	No	-	
b0614	b4505	citX	insX	No	-	
b0614	b2980	citX	glcC	No	1	
b0614	b3992	citX	thiF	No	1	
b0616	b4505	citE	insX	No	-	
b0616	b0889	citE	lrp	No	-	
b0616	b1176	citE	minC	No	-	
b0616	b1394	citE	paaG	No	-	

Continued on next page

Table S3: **Locus tag and gene id of detected protein interaction in Y2H data.** Locus tag and gene name of interacting proteins are listed in the first four columns. The existence of the interaction in other PPI networks is shown in the fifth column. The sixth and seventh column highlights whether the interacting proteins has been detected in a PPI module and whether one of the proteins is a central protein in a PPI module. (Continued from previous page)

Locus tag1	Locus tag2	Gene1	Gene2	Existing in other data sources	Id of a detected module	Centeral protein
b0616	b3021	citE	mqsA	No	-	
b0616	b3773	citE	ilvY	No	-	
b0616	b4367	citE	fhuF	No	-	
b0618	b4505	citC	insX	No	-	
b0618	b2907	citC	ubiH	No	-	
b0618	b2997	citC	hybO	No	-	
b0620	b4505	dpiA	insX	No	-	
b0620	b1176	dpiA	minC	No	-	
b0620	b1390	dpiA	paaC	No	-	
b0620	b2440	dpiA	eutC	No	-	
b0620	b2980	dpiA	glcC	No	-	
b0620	b2997	dpiA	hybO	No	-	
b0620	b4379	dpiA	yjjW	No	-	
b0620	b1391	dpiA	paaD	No	-	
b0620	b2769	dpiA	ygcQ	No	-	
b4581	b1156	ybeM	tfaE	No	-	
b0628	b0628	lipA	lipA	Yes	10	
b0640	b0044	holA	fixX	No	-	
b0640	b0226	holA	dinJ	No	-	
b0640	b4505	holA	insX	No	-	
b0640	b0851	holA	nfsA	No	-	
b0640	b0985	holA	gfcC	No	-	
b0640	b1099	holA	holB	Yes	-	
b0640	b1166	holA	ariR	No	-	
b0640	b1176	holA	minC	No	-	
b0640	b2006	holA	yeeW	No	-	
b0640	b2285	holA	nuoE	No	-	
b0640	b2562	holA	yfhL	No	-	
b0640	b2980	holA	glcC	No	-	
b0640	b3573	holA	ysaA	No	-	
b0640	b3651	holA	trmH	No	-	
b0640	b3890	holA	yiiF	No	-	
b0640	b4084	holA	alsK	No	-	
b0657	b1362	lnt	rzpR	No	-	
b0662	b0662	ubiF	ubiF	Yes	219	
b0674	b0674	asnB	asnB	Yes	36	
b0684	b1499	fldA	ydeO	No	-	
b0684	b2698	fldA	recX	No	-	
b0684	b2830	fldA	rppH	No	-	
b0684	b2980	fldA	glcC	No	-	
b0684	b3185	fldA	rpmA	No	-	
b0684	b3296	fldA	rpsD	No	-	

Continued on next page

Table S3: **Locus tag and gene id of detected protein interaction in Y2H data.** Locus tag and gene name of interacting proteins are listed in the first four columns. The existence of the interaction in other PPI networks is shown in the fifth column. The sixth and seventh column highlights whether the interacting proteins has been detected in a PPI module and whether one of the proteins is a central protein in a PPI module. (Continued from previous page)

**Locus tag1**	**Locus tag2**	**Gene1**	**Gene2**	**Existing in other data sources**	**Id of a detected module**	**Centeral protein**
b0684	b3303	fldA	rpsE	Yes	-	RpsE
b0684	b4118	fldA	melR	No	-	
b0684	b1361	fldA	ydaW	No	-	
b0684	b1831	fldA	proQ	No	-	
b0684	b3825	fldA	pldB	No	-	
b0687	b0687	seqA	seqA	Yes	355	
b0687	b0779	seqA	uvrB	No	-	
b0688	b0688	pgm	pgm	No	55	
b0700	b0525	rhsC	ppiB	Yes	201	
b0708	b0708	phr	phr	Yes	248	
b0709	b2584	dtpD	pka	No	-	
b0719	b4145	ybgD	yjeJ	No	-	
b0720	b0720	gltA	gltA	Yes	104	GltA
b0726	b0726	sucA	sucA	Yes	269	
b0727	b0726	sucB	sucA	Yes	269	
b0727	b0727	sucB	sucB	Yes	269	
b0738	b1399	tolR	paaX	No	-	
b0741	b1566	pal	flxA	No	-	
b0742	b2980	ybgF	glcC	No	-	
b0742	b3262	ybgF	yhdJ	No	-	
b0757	b0044	galK	fixX	No	-	
b0757	b0172	galK	frr	No	-	
b0757	b1156	galK	tfaE	No	-	
b0757	b3043	galK	ygiL	No	-	
b0759	b0044	galE	fixX	No	-	
b0759	b0172	galE	frr	No	-	
b0759	b0530	galE	sfmA	No	-	
b0759	b1156	galE	tfaE	No	-	
b0759	b1674	galE	ydhY	No	-	
b0759	b2713	galE	hydN	No	-	
b0759	b2792	galE	yqcC	No	-	
b0759	b3043	galE	ygiL	No	-	
b0759	b3481	galE	nikR	No	-	
b0759	b4379	galE	yjjW	No	-	
b0759	b4502	galE	yeiW	No	-	
b0759	b4342	galE	yjiT	No	-	
b0761	b4379	modE	yjjW	No	-	
b0765	b4471	modC	tdcG	No	-	
b0775	b0223	bioB	yafJ	No	-	
b0775	b1264	bioB	trpE	No	-	TrpE
b0775	b2440	bioB	eutC	No	-	
b0775	b2755	bioB	ygbT	No	-	

Continued on next page

Table S3: **Locus tag and gene id of detected protein interaction in Y2H data.** Locus tag and gene name of interacting proteins are listed in the first four columns. The existence of the interaction in other PPI networks is shown in the fifth column. The sixth and seventh column highlights whether the interacting proteins has been detected in a PPI module and whether one of the proteins is a central protein in a PPI module. (Continued from previous page)

Locus tag1	Locus tag2	Gene1	Gene2	Existing in other data sources	Id of a detected module	Centeral protein
b0775	b4016	bioB	aceK	No	-	
b0775	b1831	bioB	proQ	No	-	
b0777	b1585	bioC	ynfC	No	-	
b0779	b4505	uvrB	insX	No	-	
b0779	b0601	uvrB	ybdM	No	-	
b0779	b0640	uvrB	holA	No	-	
b0779	b0779	uvrB	uvrB	Yes	244	
b0779	b1705	uvrB	ydiE	No	-	
b0779	b2243	uvrB	glpC	No	-	
b0779	b2440	uvrB	eutC	No	-	
b0779	b2980	uvrB	glcC	No	-	
b0779	b4118	uvrB	melR	No	-	
b0779	b0535	uvrB	fimZ	No	-	
b0780	b0477	ybhK	gsk	Yes	40	YbhK
b0784	b0785	moaD	moaE	Yes	196	
b0784	b2149	moaD	mglA	No	-	
b0784	b3021	moaD	mqsA	No	-	
b0784	b4587	moaD	insN	No	-	
b0785	b4505	moaE	insX	No	-	
b0785	b0785	moaE	moaE	Yes	196	
b0785	b1183	moaE	umuD	No	-	
b0785	b1705	moaE	ydiE	No	-	
b0785	b1895	moaE	uspC	No	-	
b0785	b2440	moaE	eutC	No	-	
b0785	b2980	moaE	glcC	No	-	
b0785	b3481	moaE	nikR	No	-	
b0785	b0872	moaE	hcr	No	-	
b0796	b0796	ybiH	ybiH	No	31	
b0796	b2907	ybiH	ubiH	No	-	
b0801	b0801	ybiC	ybiC	No	141	
b0809	b0809	glnQ	glnQ	Yes	270	
b0819	b2107	ybiS	rcnB	Yes	169	
b0827	b0827	moeA	moeA	Yes	92	
b0838	b0838	gstB	gstB	No	25	
b0838	b2022	gstB	hisB	Yes	25	
b0848	b2907	ybjM	ubiH	No	-	
b0848	b2980	ybjM	glcC	No	-	
b0848	b1831	ybjM	proQ	No	-	
b0849	b0199	grxA	metN	No	-	MetN
b0849	b0601	grxA	ybdM	No	-	
b0849	b1156	grxA	tfaE	No	-	
b0849	b3481	grxA	nikR	No	-	

Continued on next page

Table S3: **Locus tag and gene id of detected protein interaction in Y2H data.** Locus tag and gene name of interacting proteins are listed in the first four columns. The existence of the interaction in other PPI networks is shown in the fifth column. The sixth and seventh column highlights whether the interacting proteins has been detected in a PPI module and whether one of the proteins is a central protein in a PPI module. (Continued from previous page)

**Locus tag1**	**Locus tag2**	**Gene1**	**Gene2**	**Existing in other data sources**	**Id of a detected module**	**Centeral protein**
b0853	b0601	ybjN	ybdM	No	-	
b0853	b1577	ybjN	ydfE	No	-	
b0853	b1705	ybjN	ydiE	No	-	
b0853	b2440	ybjN	eutC	No	-	
b0853	b2646	ybjN	ypjF	No	-	
b0853	b3651	ybjN	trmH	No	-	
b0853	b4587	ybjN	insN	No	-	
b0856	b0855	potH	potG	Yes	84	
b0861	b0861	artM	artM	Yes	87	
b0864	b0199	artP	metN	No	-	MetN
b0864	b2273	artP	yfbN	No	-	YfbN
b0864	b4153	artP	frdB	No	-	
b0864	b0946	artP	zapC	No	-	
b0864	b3357	artP	crp	No	-	
b0873	b0601	hcp	ybdM	No	-	
b0873	b0873	hcp	hcp	No	66	
b0873	b2440	hcp	eutC	No	-	
b0880	b3541	cspD	dppD	Yes	-	
b0882	b0044	clpA	fixX	No	-	
b0882	b3021	clpA	mqsA	No	-	
b0882	b4587	clpA	insN	No	-	
b0882	b4502	clpA	yeiW	No	-	
b0882	[]	clpA	[]	No	-	
b0884	b1566	infA	flxA	No	-	
b0884	b3021	infA	mqsA	No	-	
b0884	b3010	infA	yqhC	No	-	
b0885	b0058	aat	rluA	No	-	
b0885	b2962	aat	yggX	No	-	
b0888	b0195	trxB	tsaA	No	-	
b0888	b0470	trxB	dnaX	No	-	
b0888	b1174	trxB	minE	No	-	
b0888	b1797	trxB	yeaR	No	-	
b0888	b2009	trxB	sbmC	No	-	
b0888	b2232	trxB	ubiG	No	-	
b0888	b4127	trxB	yjdJ	No	-	
b0888	b1831	trxB	proQ	No	-	
b0889	b0048	lrp	folA	Yes	-	FolA
b0893	b0893	serS	serS	Yes	132	SerS
b0902	b1446	pflA	ydcY	No	-	
b0910	b1176	cmk	minC	No	-	
b0910	b1831	cmk	proQ	No	-	
b0910	b3825	cmk	pldB	No	-	

Continued on next page

Table S3: **Locus tag and gene id of detected protein interaction in Y2H data.** Locus tag and gene name of interacting proteins are listed in the first four columns. The existence of the interaction in other PPI networks is shown in the fifth column. The sixth and seventh column highlights whether the interacting proteins has been detected in a PPI module and whether one of the proteins is a central protein in a PPI module. (Continued from previous page)

Locus tag1	Locus tag2	Gene1	Gene2	Existing in other data sources	Id of a detected module	Centeral protein
b0918	b4505	kdsB	insX	No	-	KdsB
b0918	b1013	kdsB	rutR	No	-	KdsB
b0920	b4505	ycbC	insX	No	-	
b0920	b1156	ycbC	tfaE	No	-	
b0920	b3732	ycbC	atpD	No	-	
b0923	b2980	mukE	glcC	No	-	
b0923	b3262	mukE	yhdJ	No	-	
b0927	b3472	ycbL	dcrB	No	-	
b0928	b0928	aspC	aspC	Yes	32	
b0929	b1489	ompF	dosP	No	-	
b0931	b3430	pncB	glgC	No	-	
b0937	b1372	ssuE	stfR	No	111	
b0939	b1787	elfD	yeaK	No	-	
b0954	b1831	fabA	proQ	No	-	
b0956	b0779	matP	uvrB	No	-	
b0956	b2980	matP	glcC	No	-	
b0956	b0644	matP	ybeQ	No	-	
b0956	b1117	matP	lolD	No	-	
b0956	b2714	matP	ascG	No	-	
b0956	b3357	matP	crp	No	-	
b0959	b1120	sxy	cobB	Yes	126	
b0969	b0189	tusE	rof	No	-	
b0969	b0779	tusE	uvrB	No	-	
b0972	b0977	hyaA	hyaF	No	-	
b0973	b0975	hyaB	hyaD	No	-	
b0975	b0972	hyaD	hyaA	No	-	
b0975	b0973	hyaD	hyaB	No	-	
b0975	b0975	hyaD	hyaD	No	205	
b0975	b2970	hyaD	yghF	No	205	
b0985	b2701	gfcC	mltB	No	43	
b4517	b0199	gnsA	metN	No	-	MetN
b4517	b4517	gnsA	gnsA	No	190	
b4517	b4379	gnsA	yjjW	No	-	
b1013	b2980	rutR	glcC	No	-	
b1015	b1517	putP	lsrF	No	-	
b1015	b2232	putP	ubiG	No	-	
b1015	b3021	putP	mqsA	No	-	
b1021	b0439	pgaD	lon	No	-	
b1021	b1021	pgaD	pgaD	No	316	
b1021	b1290	pgaD	sapF	No	316	
b1021	b1961	pgaD	dcm	No	-	
b1021	b3236	pgaD	mdh	No	-	

Continued on next page

Table S3: **Locus tag and gene id of detected protein interaction in Y2H data.** Locus tag and gene name of interacting proteins are listed in the first four columns. The existence of the interaction in other PPI networks is shown in the fifth column. The sixth and seventh column highlights whether the interacting proteins has been detected in a PPI module and whether one of the proteins is a central protein in a PPI module. (Continued from previous page)

Locus tag1	Locus tag2	Gene1	Gene2	Existing in other data sources	Id of a detected module	Centeral protein
b1021	b3697	pgaD	yidA	No	-	
b1021	b3706	pgaD	mnmE	Yes	316	
b1040	b1040	csgD	csgD	No	41	
b1062	b1088	pyrC	yceD	No	-	
b1062	b2273	pyrC	yfbN	No	-	YfbN
b1062	b3418	pyrC	malT	No	-	
b1062	b3651	pyrC	trmH	No	-	
b1077	b1952	flgF	dsrB	No	-	
b1082	b1033	flgK	ghrA	No	-	
b1096	b1096	pabC	pabC	Yes	143	
b1103	b1103	hinT	hinT	Yes	576	
b1118	b1104	lolE	ycfL	No	-	
b1118	b1816	lolE	yoaE	No	-	
b1118	b2167	lolE	fruA	No	-	
b1118	b4461	lolE	yfjD	No	-	
b1118	b3010	lolE	yqhC	No	-	
b1120	b1120	cobB	cobB	Yes	126	
b1127	b1127	pepT	pepT	Yes	506	
b1127	b3021	pepT	mqsA	No	-	
b1134	b0199	nudJ	metN	No	-	MetN
b1135	b1593	rluE	ynfK	No	-	
b1158	b0044	pinE	fixX	No	-	
b1175	b1176	minD	minC	Yes	-	
b1175	b1547	minD	stfQ	No	-	
b1175	b2562	minD	yfhL	No	-	
b1175	b0691	minD	ybfG	No	-	
b1176	b4379	minC	yjjW	No	-	
b1179	b4379	ycgL	yjjW	No	-	
b1192	b4379	ldcA	yjjW	No	-	
b1194	b3262	ycgR	yhdJ	No	1	
b1205	b3734	ychH	atpA	No	-	
b1224	b1226	narG	narJ	Yes	-	
b1233	b0413	ychJ	nrdR	No	-	
b1243	b3813	oppA	uvrD	No	-	
b1247	b1394	oppF	paaG	No	-	
b1248	b1248	yciU	yciU	Yes	80	
b1248	b0296	yciU	ykgM	No	-	
b1258	b1258	yciF	yciF	Yes	120	
b1262	b0044	trpC	fixX	No	-	
b1262	b2232	trpC	ubiG	No	-	
b1262	b3043	trpC	ygiL	No	-	
b1262	b2846	trpC	yqeH	No	-	

Continued on next page

Table S3: **Locus tag and gene id of detected protein interaction in Y2H data.** Locus tag and gene name of interacting proteins are listed in the first four columns. The existence of the interaction in other PPI networks is shown in the fifth column. The sixth and seventh column highlights whether the interacting proteins has been detected in a PPI module and whether one of the proteins is a central protein in a PPI module. (Continued from previous page)

**Locus tag1**	**Locus tag2**	**Gene1**	**Gene2**	**Existing in other data sources**	**Id of a detected module**	**Centeral protein**
b1262	b4342	trpC	yjiT	No	-	
b1275	b1275	cysB	cysB	Yes	219	
b1275	b4109	cysB	yjdA	No	-	
b1276	b0036	acnA	caiD	No	-	
b1284	b1953	yciT	yodD	No	26	
b1302	b1846	puuE	yebE	No	-	
b1320	b1320	ycjW	ycjW	No	71	
b1324	b1108	tpx	ycfP	No	11	
b1330	b0044	ynaI	fixX	No	-	
b1330	b0530	ynaI	sfmA	No	-	
b1330	b1105	ynaI	lpoB	No	-	
b1342	b1342	ydaN	ydaN	No	75	
b1360	b1360	ydaV	ydaV	No	592	
b1414	b4502	ydcF	yeiW	No	-	
b1423	b4505	ydcJ	insX	No	-	
b1423	b3021	ydcJ	mqsA	No	-	
b1423	b3479	ydcJ	nikD	No	-	
b1423	b3806	ydcJ	cyaA	No	-	
b1423	b4587	ydcJ	insN	No	-	
b1446	b0044	ydcY	fixX	No	-	
b1446	b0172	ydcY	frr	No	-	
b1446	b0199	ydcY	metN	No	-	MetN
b1446	b4505	ydcY	insX	No	-	
b1446	b0902	ydcY	pflA	No	-	
b1446	b3231	ydcY	rplM	Yes	2	RplM
b1446	b0872	ydcY	hcr	No	-	
b1446	b1831	ydcY	proQ	No	-	
b1474	b3891	fdnG	fdhE	Yes	-	
b1482	b0172	osmC	frr	No	-	OsmC
b1482	b1482	osmC	osmC	No	25	OsmC
b1482	b2418	osmC	pdxK	No	-	OsmC
b1486	b3541	ddpB	dppD	No	-	
b1493	b0199	gadB	metN	No	-	MetN
b1493	b2107	gadB	rcnB	No	-	
b1494	b0399	pqqL	phoB	No	-	
b1518	b0172	lsrG	frr	No	-	
b1539	b1539	ydfG	ydfG	Yes	59	YdfG
b1566	b0421	flxA	ispA	Yes	49	IspA
b1566	b1566	flxA	flxA	No	49	
b1566	b2460	flxA	eutQ	No	49	EutQ
b1603	b1603	pntA	pntA	Yes	281	
b1607	b2676	ydgC	nrdF	No	-	

Continued on next page

Table S3: **Locus tag and gene id of detected protein interaction in Y2H data.** Locus tag and gene name of interacting proteins are listed in the first four columns. The existence of the interaction in other PPI networks is shown in the fifth column. The sixth and seventh column highlights whether the interacting proteins has been detected in a PPI module and whether one of the proteins is a central protein in a PPI module. (Continued from previous page)

Locus tag1	Locus tag2	Gene1	Gene2	Existing in other data sources	Id of a detected module	Centeral protein
b1612	b1612	fumA	fumA	Yes	16	
b1618	b0044	uidR	fixX	No	-	
b1618	b1618	uidR	uidR	No	232	
b1618	b1794	uidR	yeaP	No	232	
b1626	b0903	ydgK	pflB	No	-	
b1631	b1631	rsxG	rsxG	No	483	
b1639	b3400	mliC	hslR	Yes	450	
b1641	b0964	slyB	yccT	No	-	
b1645	b4505	ydhK	insX	No	-	
b1651	b0172	gloA	frr	No	-	
b1680	b0199	sufS	metN	No	-	MetN
b1680	b2755	sufS	ygbT	No	-	
b1680	b3357	sufS	crp	No	-	
b1681	b0199	sufD	metN	No	-	MetN
b1681	b1682	sufD	sufC	Yes	379	
b1682	b1683	sufC	sufB	Yes	379	
b1694	b0199	ydiF	metN	No	-	MetN
b1703	b1703	ppsR	ppsR	Yes	234	
b1706	b0044	ydiU	fixX	No	-	
b1706	b2232	ydiU	ubiG	No	-	
b1706	b3043	ydiU	ygiL	No	-	
b1706	b2846	ydiU	yqeH	No	-	
b1706	b4342	ydiU	yjiT	No	-	
b1712	b0779	ihfA	uvrB	No	-	
b1713	b1713	pheT	pheT	Yes	291	
b1713	b4081	pheT	mdtO	No	-	
b1719	b1719	thrS	thrS	Yes	103	ThrS
b1732	b1732	katE	katE	Yes	43	
b1732	b1922	katE	fliA	No	-	
b1740	b1566	nadE	flxA	Yes	-	NadE
b1740	b1575	nadE	dicB	No	-	NadE
b1740	b1740	nadE	nadE	Yes	5	NadE
b1741	b2980	cho	glcC	No	1	
b1744	b0464	astE	acrR	No	-	
b1744	b2755	astE	ygbT	No	-	
b1744	b3262	astE	yhdJ	No	1	
b1744	b4480	astE	hdfR	No	-	
b1748	b2086	astC	yegS	No	104	
b1776	b1776	ydjL	ydjL	No	395	
b1785	b0946	yeaI	zapC	No	96	
b1807	b0464	tsaB	acrR	No	-	
b1807	b1807	tsaB	tsaB	Yes	133	

Continued on next page

Table S3: **Locus tag and gene id of detected protein interaction in Y2H data.** Locus tag and gene name of interacting proteins are listed in the first four columns. The existence of the interaction in other PPI networks is shown in the fifth column. The sixth and seventh column highlights whether the interacting proteins has been detected in a PPI module and whether one of the proteins is a central protein in a PPI module. (Continued from previous page)

Locus tag1	Locus tag2	Gene1	Gene2	Existing in other data sources	Id of a detected module	Centeral protein
b1817	b1817	manX	manX	Yes	429	
b1817	b3794	manX	wecG	No	-	
b1823	b1823	cspC	cspC	Yes	8	CspC
b1842	b0215	holE	dnaQ	Yes	-	
b1849	b0421	purT	ispA	Yes	49	IspA
b1849	b1849	purT	purT	No	49	
b1850	b1850	eda	eda	Yes	37	
b1854	b0657	pykA	lnt	Yes	436	
b1854	b1621	pykA	malX	Yes	-	
b1854	b1854	pykA	pykA	Yes	436	
b1861	b1861	ruvA	ruvA	Yes	10	
b1864	b0009	yebC	mog	No	-	YebC
b1864	b2127	yebC	mlrA	No	-	YebC
b1864	b3262	yebC	yhdJ	No	-	YebC
b1864	b3825	yebC	pldB	No	-	YebC
b1868	b0897	yecE	ycaC	Yes	245	
b1868	b1868	yecE	yecE	No	245	
b1895	b0640	uspC	holA	No	-	
b1900	b0046	araG	kefF	No	-	
b1900	b0197	araG	metQ	Yes	-	
b1900	b0325	araG	yahK	No	-	
b1900	b1900	araG	araG	Yes	385	
b1902	b0172	ftnB	frr	No	-	
b1902	b3021	ftnB	mqsA	No	-	
b1902	b3479	ftnB	nikD	No	-	
b1902	b4587	ftnB	insN	No	-	
b1913	b1913	uvrC	uvrC	Yes	480	
b1926	b0601	fliT	ybdM	No	-	
b1928	b4505	yedD	insX	No	-	
b1928	b0775	yedD	bioB	No	-	
b1928	b4367	yedD	fhuF	No	-	
b1942	b2980	fliJ	glcC	No	-	
b1943	b0356	fliK	frmA	No	-	
b1943	b2006	fliK	yeeW	No	-	
b1943	b3408	fliK	feoA	No	-	
b1943	b4137	fliK	cutA	No	-	
b1945	b0044	fliM	fixX	No	-	
b1945	b4505	fliM	insX	No	-	
b1960	b2980	vsr	glcC	No	-	
b1962	b0044	yedJ	fixX	No	-	
b1962	b4505	yedJ	insX	No	-	
b1962	b1831	yedJ	proQ	No	-	

Continued on next page

Table S3: **Locus tag and gene id of detected protein interaction in Y2H data.** Locus tag and gene name of interacting proteins are listed in the first four columns. The existence of the interaction in other PPI networks is shown in the fifth column. The sixth and seventh column highlights whether the interacting proteins has been detected in a PPI module and whether one of the proteins is a central protein in a PPI module. (Continued from previous page)

Locus tag1	Locus tag2	Gene1	Gene2	Existing in other data sources	Id of a detected module	Centeral protein
b1963	b2462	yedR	eutS	No	-	
b1968	b2755	yedV	ygbT	No	-	
b1970	b2012	hiuH	yeeD	No	-	
b1973	b2830	zinT	rppH	No	-	
b1973	b3745	zinT	viaA	No	-	
b1976	b0034	mtfA	caiF	No	-	
b1976	b0245	mtfA	ykfI	No	-	
b1976	b4023	mtfA	yjbD	No	-	
b1976	b3084	mtfA	rlmG	No	-	
b1976	b3745	mtfA	viaA	No	-	
b2004	b0172	cbeA	frr	No	-	
b2004	b2418	cbeA	pdxK	No	-	
b2006	b0172	yeeW	frr	No	-	
b2006	b1831	yeeW	proQ	No	-	
b2020	b0127	hisD	yadG	No	-	
b2022	b2022	hisB	hisB	Yes	25	
b2025	b0199	hisF	metN	No	-	MetN
b2025	b0662	hisF	ubiF	No	-	
b2025	b2562	hisF	yfhL	No	-	
b2025	b4367	hisF	fhuF	No	-	
b2028	b0779	ugd	uvrB	No	-	
b2029	b2029	gnd	gnd	Yes	24	
b2032	b1108	wbbK	ycfP	Yes	11	WbbK
b2041	b0199	rfbB	metN	No	-	MetN
b2041	b1014	rfbB	putA	No	25	
b2041	b2041	rfbB	rfbB	Yes	25	
b2041	b1252	rfbB	tonB	No	-	
b2057	b1390	wcaC	paaC	No	-	
b2066	b2066	udk	udk	No	13	
b2066	b2755	udk	ygbT	No	-	
b2073	b0199	yegL	metN	No	-	MetN
b2073	b0464	yegL	acrR	No	-	
b2073	b3021	yegL	mqsA	No	-	
b2086	b0720	yegS	gltA	Yes	104	GltA
b2093	b0172	gatB	frr	No	-	
b2095	b0199	gatZ	metN	No	-	MetN
b2118	b4505	yehI	insX	No	-	
b2118	b3021	yehI	mqsA	No	-	
b2118	b3170	yehI	rimP	No	-	
b2129	b4016	yehX	aceK	No	-	
b2130	b2130	yehY	yehY	Yes	302	
b2143	b2143	cdd	cdd	Yes	159	

Continued on next page

Table S3: **Locus tag and gene id of detected protein interaction in Y2H data.** Locus tag and gene name of interacting proteins are listed in the first four columns. The existence of the interaction in other PPI networks is shown in the fifth column. The sixth and seventh column highlights whether the interacting proteins has been detected in a PPI module and whether one of the proteins is a central protein in a PPI module. (Continued from previous page)

Locus tag1	Locus tag2	Gene1	Gene2	Existing in other data sources	Id of a detected module	Centeral protein
b2150	b3749	mglB	rbsA	No	-	
b2155	b3734	cirA	atpA	Yes	38	
b2166	b4505	psuK	insX	No	-	
b2166	b2980	psuK	glcC	No	-	
b2169	b0559	fruB	ybcW	No	-	
b2169	b2169	fruB	fruB	Yes	563	
b2173	b1472	yeiR	yddL	No	-	
b2173	b1652	yeiR	rnt	No	-	
b2181	b4145	yejG	yjeJ	No	-	
b2193	b0223	narP	yafJ	No	-	
b2193	b1117	narP	lolD	No	-	
b2212	b4505	alkB	insX	No	-	
b2212	b3021	alkB	mqsA	No	-	
b2213	b2456	ada	eutN	No	-	
b2213	b4016	ada	aceK	No	-	
b2213	b2496	ada	hda	No	-	
b2217	b2216	rcsB	rcsD	Yes	12	RcsB
b2217	b2217	rcsB	rcsB	Yes	12	RcsB
b2217	b3021	rcsB	mqsA	No	-	RcsB
b2232	b4505	ubiG	insX	No	-	
b2232	b2907	ubiG	ubiH	No	-	
b2232	b2980	ubiG	glcC	No	-	
b2248	b0467	rhmR	priC	No	-	
b2248	b2248	rhmR	rhmR	No	-	
b2255	b0044	arnA	fixX	No	-	
b2259	b1952	pmrD	dsrB	No	-	
b2262	b2262	menB	menB	Yes	9	
b2268	b2554	rbn	glrR	No	-	
b2272	b0855	yfbM	potG	No	-	
b2273	b3001	yfbN	gpr	No	-	YfbN
b2273	b3017	yfbN	ftsP	Yes	-	YfbN
b2275	b2980	yfbP	glcC	No	-	
b2283	b2283	nuoG	nuoG	Yes	35	
b2283	b2285	nuoG	nuoE	Yes	35	
b2283	b3006	nuoG	exbB	No	-	
b2285	b2283	nuoE	nuoG	Yes	35	
b2285	b2285	nuoE	nuoE	Yes	35	
b2289	b2878	lrhA	ygfK	No	-	YgfK
b2297	b2293	pta	yfbT	No	-	
b2299	b0133	yfcD	panC	Yes	-	
b2306	b0413	hisP	nrdR	No	-	
b2306	b0946	hisP	zapC	No	-	

Continued on next page

Table S3: **Locus tag and gene id of detected protein interaction in Y2H data.** Locus tag and gene name of interacting proteins are listed in the first four columns. The existence of the interaction in other PPI networks is shown in the fifth column. The sixth and seventh column highlights whether the interacting proteins has been detected in a PPI module and whether one of the proteins is a central protein in a PPI module. (Continued from previous page)

**Locus tag1**	**Locus tag2**	**Gene1**	**Gene2**	**Existing in other data sources**	**Id of a detected module**	**Centeral protein**
b2316	b0185	accD	accA	Yes	-	
b2316	b0956	accD	matP	No	-	
b2316	b1176	accD	minC	No	-	
b2316	b1183	accD	umuD	No	-	
b2316	b1390	accD	paaC	No	-	
b2316	b1895	accD	uspC	No	-	
b2316	b2595	accD	bamD	No	-	
b2316	b4367	accD	fhuF	No	-	
b2316	b3262	accD	yhdJ	No	-	
b2318	b0044	truA	fixX	No	-	
b2318	b0172	truA	frr	No	-	
b2318	b2012	truA	yeeD	No	-	
b2318	b1831	truA	proQ	No	-	
b2318	b4342	truA	yjiT	No	-	
b2322	b0570	yfcJ	cusS	No	-	CusS
b2325	b1703	yfcL	ppsR	No	-	
b2331	b1176	smrB	minC	No	-	
b2331	b1513	smrB	lsrA	No	-	
b2331	b1895	smrB	uspC	No	-	
b2331	b2980	smrB	glcC	No	-	
b2331	b3560	smrB	glyQ	No	-	
b2331	b3954	smrB	yijO	No	-	
b2340	b0453	sixA	ybaY	No	-	
b2340	b1973	sixA	zinT	No	-	
b2340	b2173	sixA	yeiR	No	-	
b2340	b2957	sixA	ansB	No	-	
b2340	b3021	sixA	mqsA	No	-	
b2340	b2107	sixA	rcnB	No	-	
b2340	b4501	sixA	torI	No	-	
b2346	b0185	mlaA	accA	No	-	
b2346	b1261	mlaA	trpB	No	-	
b2346	b4471	mlaA	tdcG	No	-	
b2360	b2532	yfdQ	trmJ	No	-	
b2366	b0199	dsdA	metN	No	-	MetN
b2369	b0464	evgA	acrR	No	-	
b2369	b2012	evgA	yeeD	No	-	
b2369	b2263	evgA	menH	No	-	
b2369	b2731	evgA	fhlA	No	-	
b2369	b3868	evgA	glnG	No	-	
b2369	b4118	evgA	melR	No	-	
b2369	b0644	evgA	ybeQ	No	-	
b2369	b1831	evgA	proQ	No	-	

Continued on next page

Table S3: **Locus tag and gene id of detected protein interaction in Y2H data.** Locus tag and gene name of interacting proteins are listed in the first four columns. The existence of the interaction in other PPI networks is shown in the fifth column. The sixth and seventh column highlights whether the interacting proteins has been detected in a PPI module and whether one of the proteins is a central protein in a PPI module. (Continued from previous page)

**Locus tag1**	**Locus tag2**	**Gene1**	**Gene2**	**Existing in other data sources**	**Id of a detected module**	**Centeral protein**
b2369	b4342	evgA	yjiT	No	-	
b2373	b2373	oxc	oxc	Yes	289	
b2374	b0199	frc	metN	No	-	MetN
b2374	b0413	frc	nrdR	No	-	
b2379	b0199	alaC	metN	No	-	MetN
b2379	b2379	alaC	alaC	No	499	
b2384	b0199	ypdE	metN	No	-	MetN
b2384	b1156	ypdE	tfaE	No	-	
b2384	b2379	ypdE	alaC	Yes	-	
b2384	b3481	ypdE	nikR	No	-	
b2409	b0199	yfeR	metN	No	-	MetN
b2412	b0095	zipA	ftsZ	Yes	-	
b2414	b0199	cysK	metN	No	-	MetN
b2418	b2123	pdxK	yehR	No	-	
b2418	b2714	pdxK	ascG	No	-	
b2420	b3021	yfeS	mqsA	No	-	
b2420	b2107	yfeS	rcnB	No	-	
b2421	b0530	cysM	sfmA	No	-	
b2421	b0712	cysM	ybgK	No	-	
b2421	b0719	cysM	ybgD	No	-	
b2421	b0933	cysM	ssuB	No	-	
b2421	b1247	cysM	oppF	No	-	
b2421	b1973	cysM	zinT	No	-	
b2421	b2012	cysM	yeeD	No	-	
b2421	b2149	cysM	mglA	No	-	
b2421	b2335	cysM	yfcR	No	-	
b2421	b2713	cysM	hydN	No	-	
b2421	b3215	cysM	yhcA	No	-	
b2421	b1831	cysM	proQ	No	-	
b2438	b4505	eutK	insX	No	-	
b2438	b2149	eutK	mglA	No	-	
b2438	b2273	eutK	yfbN	No	-	YfbN
b2440	b2451	eutC	eutA	No	-	
b2451	b0601	eutA	ybdM	No	-	
b2451	b2440	eutA	eutC	No	-	
b2456	b1176	eutN	minC	No	-	
b2456	b1705	eutN	ydiE	No	-	
b2456	b2143	eutN	cdd	No	-	
b2458	b0601	eutD	ybdM	No	-	EutD
b2460	b2458	eutQ	eutD	No	-	EutQ, EutD
b2460	b2460	eutQ	eutQ	No	49	EutQ
b2460	b2868	eutQ	xdhC	No	-	EutQ

Continued on next page

Table S3: **Locus tag and gene id of detected protein interaction in Y2H data.** Locus tag and gene name of interacting proteins are listed in the first four columns. The existence of the interaction in other PPI networks is shown in the fifth column. The sixth and seventh column highlights whether the interacting proteins has been detected in a PPI module and whether one of the proteins is a central protein in a PPI module. (Continued from previous page)

Locus tag1	Locus tag2	Gene1	Gene2	Existing in other data sources	Id of a detected module	Centeral protein
b2471	b0172	yffB	frr	No	-	
b2471	b0199	yffB	metN	No	-	MetN
b2471	b0902	yffB	pflA	No	-	
b2471	b2440	yffB	eutC	No	-	
b2471	b2471	yffB	yffB	No	168	
b2471	b0872	yffB	hcr	No	-	
b2471	b1831	yffB	proQ	No	-	
b2472	b2012	dapE	yeeD	No	-	
b2472	b2055	dapE	wcaE	No	-	
b2472	b4237	dapE	nrdG	No	-	
b2479	b0172	gcvR	frr	No	-	
b2479	b2701	gcvR	mltB	No	-	
b2480	b2456	bcp	eutN	No	-	
b2491	b2149	hyfR	mglA	No	-	
b2491	b3357	hyfR	crp	No	-	
b2495	b1334	yfgD	fnr	No	-	
b2495	b1973	yfgD	zinT	No	-	
b2495	b3021	yfgD	mqsA	No	-	
b2495	b3262	yfgD	yhdJ	No	-	
b2495	b3357	yfgD	crp	No	-	
b2498	b2498	upp	upp	Yes	44	
b2499	b0719	purM	ybgD	No	-	
b2501	b0200	ppk	gmhB	No	-	
b2501	b0223	ppk	yafJ	No	-	
b2501	b1717	ppk	rpmI	No	-	
b2501	b3239	ppk	yhcO	No	-	
b2501	b3953	ppk	frwD	No	-	
b2501	b0946	ppk	zapC	No	-	
b2502	b0199	ppx	metN	No	-	MetN
b2502	b2502	ppx	ppx	Yes	178	
b2502	b4299	ppx	yjhI	No	-	
b2509	b1308	xseA	pspE	No	-	
b2513	b0998	yfgM	torD	No	-	
b2517	b0095	rlmN	ftsZ	No	-	RlmN
b2517	b2517	rlmN	rlmN	Yes	99	RlmN
b2518	b0172	ndk	frr	No	-	
b2525	b0172	fdx	frr	No	-	
b2525	b1266	fdx	yciV	No	-	
b2525	b1831	fdx	proQ	No	-	
b2529	b0470	iscU	dnaX	No	-	IscU
b2529	b2527	iscU	hscB	Yes	-	IscU
b2531	b2531	iscR	iscR	No	287	

Continued on next page

Table S3: **Locus tag and gene id of detected protein interaction in Y2H data.** Locus tag and gene name of interacting proteins are listed in the first four columns. The existence of the interaction in other PPI networks is shown in the fifth column. The sixth and seventh column highlights whether the interacting proteins has been detected in a PPI module and whether one of the proteins is a central protein in a PPI module. (Continued from previous page)

Locus tag1	Locus tag2	Gene1	Gene2	Existing in other data sources	Id of a detected module	Centeral protein
b2533	b4379	suhB	yjjW	No	-	
b2542	b0044	hcaD	fixX	No	-	
b2542	b2713	hcaD	hydN	No	-	
b2542	b3043	hcaD	ygiL	No	-	
b2542	b3481	hcaD	nikR	No	-	
b2542	b4379	hcaD	yjjW	No	-	
b2554	b4379	glrR	yjjW	No	-	
b2566	b4505	era	insX	No	-	
b2566	b0956	era	matP	No	-	
b2566	b1013	era	rutR	No	-	
b2566	b2345	era	yfdF	No	-	
b2566	b3021	era	mqsA	No	-	
b2566	b2714	era	ascG	No	-	
b2566	b3262	era	yhdJ	No	1	
b2569	b0313	lepA	betI	No	-	LepA
b2569	b1342	lepA	ydaN	Yes	75	LepA
b2569	b4306	lepA	yjhP	No	-	LepA
b2572	b2573	rseA	rpoE	No	-	
b2573	b4505	rpoE	insX	No	-	
b2573	b0956	rpoE	matP	No	-	
b2573	b1620	rpoE	malI	No	-	
b2573	b2572	rpoE	rseA	No	-	
b2573	b2980	rpoE	glcC	No	-	
b2573	b3021	rpoE	mqsA	No	-	
b2573	b3262	rpoE	yhdJ	No	-	
b2580	b0044	ung	fixX	No	-	
b2580	b1117	ung	lolD	No	-	
b2592	b2012	clpB	yeeD	No	-	ClpB
b2592	b1585	clpB	ynfC	No	-	ClpB
b2597	b0172	raiA	frr	No	11	
b2597	b2597	raiA	raiA	Yes	11	
b2601	b0044	aroF	fixX	No	-	
b2601	b3043	aroF	ygiL	No	-	
b2607	b2607	trmD	trmD	Yes	36	
b2617	b0414	bamE	ribD	No	-	RibD
b2620	b2620	smpB	smpB	Yes	498	
b2627	b1717	yfjK	rpmI	No	-	
b2627	b3239	yfjK	yhcO	No	-	
b2627	b3742	yfjK	mioC	No	-	
b2646	b4181	ypjF	yjfI	No	-	
b2660	b3775	lhgO	ppiC	Yes	478	
b2662	b2012	gabT	yeeD	No	-	GabT

Continued on next page

Table S3: **Locus tag and gene id of detected protein interaction in Y2H data.** Locus tag and gene name of interacting proteins are listed in the first four columns. The existence of the interaction in other PPI networks is shown in the fifth column. The sixth and seventh column highlights whether the interacting proteins has been detected in a PPI module and whether one of the proteins is a central protein in a PPI module. (Continued from previous page)

Locus tag1	Locus tag2	Gene1	Gene2	Existing in other data sources	Id of a detected module	Centeral protein
b2662	b4306	gabT	yjhP	No	-	GabT
b2669	b0964	stpA	yccT	No	-	
b2673	b2676	nrdH	nrdF	No	-	
b2675	b0779	nrdE	uvrB	No	-	
b2677	b0874	proV	ybjE	No	-	
b2677	b2677	proV	proV	Yes	312	
b2685	b2685	emrA	emrA	Yes	19	
b2688	b0199	gshA	metN	No	-	MetN
b2688	b0262	gshA	afuC	No	-	
b2696	b0466	csrA	ybaM	No	-	
b2701	b2980	mltB	glcC	No	-	
b2701	b3021	mltB	mqsA	No	-	
b2701	b3357	mltB	crp	No	-	
b2713	b0872	hydN	hcr	No	-	
b2728	b2990	hypC	hybG	Yes	155	
b2729	b2990	hypD	hybG	Yes	155	
b2730	b4505	hypE	insX	No	-	
b2730	b0909	hypE	ycaL	No	-	
b2730	b1921	hypE	fliZ	No	-	
b2730	b2721	hypE	hycE	No	-	
b2730	b2730	hypE	hypE	Yes	261	
b2730	b4398	hypE	creB	No	-	
b2730	b2712	hypE	hypF	No	-	
b2743	b4181	pcm	yjfI	No	-	
b2745	b1831	truD	proQ	No	-	
b2748	b0083	ftsB	ftsL	Yes	70	
b2748	b0096	ftsB	lpxC	No	-	
b2755	b1834	ygbT	yebT	No	-	
b2755	b1968	ygbT	yedV	No	-	
b2755	b2755	ygbT	ygbT	No	-	
b2764	b2764	cysJ	cysJ	Yes	51	
b2764	b0947	cysJ	ycbX	Yes	51	
b2786	b0882	barA	clpA	Yes	-	
b2791	b0835	truC	rimO	No	-	
b2795	b4051	ygdH	qorA	No	-	
b2799	b2799	fucO	fucO	Yes	41	
b2799	b3287	fucO	def	No	-	
b2803	b2803	fucK	fucK	No	201	
b2808	b3357	gcvA	crp	No	-	
b2810	b2810	csdA	csdA	Yes	277	
b2819	b4505	recD	insX	No	-	
b2819	b4587	recD	insN	No	-	

Continued on next page

Table S3: **Locus tag and gene id of detected protein interaction in Y2H data.** Locus tag and gene name of interacting proteins are listed in the first four columns. The existence of the interaction in other PPI networks is shown in the fifth column. The sixth and seventh column highlights whether the interacting proteins has been detected in a PPI module and whether one of the proteins is a central protein in a PPI module. (Continued from previous page)

Locus tag1	Locus tag2	Gene1	Gene2	Existing in other data sources	Id of a detected module	Centeral protein
b2829	b2582	ptsP	trxC	No	-	
b2829	b2980	ptsP	glcC	No	1	
b2829	b3351	ptsP	kefG	No	-	
b2829	b3781	ptsP	trxA	No	-	
b2833	b3204	ygdR	ptsN	No	-	
b2838	b4505	lysA	insX	No	-	
b2838	b4124	lysA	dcuR	No	-	
b2839	b4505	lysR	insX	No	-	
b2839	b4367	lysR	fhuF	No	-	
b2839	b3357	lysR	crp	No	-	
b2847	b2980	yqeI	glcC	No	-	
b2852	b2745	ygeH	truD	No	-	
b2869	b2869	ygeV	ygeV	Yes	33	
b2892	b4505	recJ	insX	No	-	
b2892	b2980	recJ	glcC	No	-	
b2894	b2894	xerD	xerD	Yes	494	
b2894	b2980	xerD	glcC	No	-	
b2903	b2904	gcvP	gcvH	Yes	24	GcvP
b2904	b1176	gcvH	minC	No	-	
b2906	b2957	visC	ansB	Yes	11	
b2918	b0464	argK	acrR	No	-	
b2918	b0601	argK	ybdM	No	-	
b2918	b1176	argK	minC	No	-	
b2918	b1394	argK	paaG	No	-	
b2918	b4367	argK	fhuF	No	-	
b2918	b0424	argK	yajL	No	-	
b2918	b1593	argK	ynfK	No	-	
b2918	b3262	argK	yhdJ	No	1	
b2925	b2925	fbaA	fbaA	Yes	518	
b2926	b1096	pgk	pabC	No	-	
b2938	b2527	speA	hscB	No	-	
b2938	b2713	speA	hydN	No	-	
b2938	b2938	speA	speA	Yes	246	
b2938	b1560	speA	ydfU	No	-	
b2947	b0946	gshB	zapC	No	-	
b2950	b2950	yggR	yggR	No	436	
b2951	b0044	yggS	fixX	No	-	
b2952	b2326	yggT	epmC	No	-	
b2952	b3967	yggT	murI	No	3	
b2955	b0044	yggW	fixX	No	-	
b2955	b2149	yggW	mglA	No	-	
b2955	b2713	yggW	hydN	No	-	

Continued on next page

Table S3: **Locus tag and gene id of detected protein interaction in Y2H data.** Locus tag and gene name of interacting proteins are listed in the first four columns. The existence of the interaction in other PPI networks is shown in the fifth column. The sixth and seventh column highlights whether the interacting proteins has been detected in a PPI module and whether one of the proteins is a central protein in a PPI module. (Continued from previous page)

Locus tag1	Locus tag2	Gene1	Gene2	Existing in other data sources	Id of a detected module	Centeral protein
b2955	b2997	yggW	hybO	No	-	
b2955	b3357	yggW	crp	No	-	
b2959	b1446	yggL	ydcY	No	-	
b2959	b2760	yggL	casA	No	-	
b2979	b0601	glcD	ybdM	No	-	
b2979	b2440	glcD	eutC	No	-	
b2990	b4505	hybG	insX	No	-	
b2990	b0909	hybG	ycaL	Yes	155	
b2990	b2721	hybG	hycE	Yes	-	
b2990	b2729	hybG	hypD	Yes	155	
b2993	b4375	hybD	prfC	Yes	227	
b3005	b3005	exbD	exbD	Yes	273	
b3006	b3006	exbB	exbB	Yes	273	
b3017	b3001	ftsP	gpr	No	-	
b3017	b3017	ftsP	ftsP	No	57	
b3021	b0464	mqsA	acrR	No	-	
b3021	b0956	mqsA	matP	No	-	
b3021	b1334	mqsA	fnr	No	-	
b3021	b1698	mqsA	ydiR	No	-	
b3021	b1705	mqsA	ydiE	No	-	
b3021	b1973	mqsA	zinT	No	-	
b3021	b2755	mqsA	ygbT	No	-	
b3021	b2980	mqsA	glcC	No	-	
b3021	b3021	mqsA	mqsA	Yes	161	
b3021	b3185	mqsA	rpmA	No	-	
b3021	b3262	mqsA	yhdJ	No	-	
b3021	b3825	mqsA	pldB	No	-	
b3021	b4342	mqsA	yjiT	No	-	
b3030	b3030	parE	parE	Yes	41	ParE
b3031	b3608	yqiA	gpsA	No	-	
b3032	b0044	cpdA	fixX	No	-	
b3041	b3041	ribB	ribB	Yes	289	
b3064	b4505	tsaD	insX	No	-	
b3064	b1807	tsaD	tsaB	Yes	133	
b3064	b3064	tsaD	tsaD	Yes	133	
b3066	b4505	dnaG	insX	No	-	
b3066	b2440	dnaG	eutC	No	-	
b3067	b0059	rpoD	rapA	No	-	
b3067	b3067	rpoD	rpoD	Yes	105	
b3067	b3995	rpoD	rsd	Yes	-	
b3070	b0640	yqjH	holA	No	-	
b3082	b2646	higA	ypjF	No	-	

Continued on next page

Table S3: **Locus tag and gene id of detected protein interaction in Y2H data.** Locus tag and gene name of interacting proteins are listed in the first four columns. The existence of the interaction in other PPI networks is shown in the fifth column. The sixth and seventh column highlights whether the interacting proteins has been detected in a PPI module and whether one of the proteins is a central protein in a PPI module. (Continued from previous page)

**Locus tag1**	**Locus tag2**	**Gene1**	**Gene2**	**Existing in other data sources**	**Id of a detected module**	**Centeral protein**
b3082	b3083	higA	higB	No	-	
b3087	b0239	ygjR	frsA	No	-	
b3087	b1183	ygjR	umuD	No	-	
b3087	b2755	ygjR	ygbT	No	-	
b3087	b3021	ygjR	mqsA	No	-	
b3087	b4016	ygjR	aceK	No	-	
b3087	b3848	ygjR	yigZ	No	-	
b3102	b0044	yqjG	fixX	No	-	YqjG
b3102	b0662	yqjG	ubiF	No	-	YqjG
b3102	b0775	yqjG	bioB	No	-	YqjG
b3105	b3106	yhaJ	yhaK	Yes	-	
b3105	b3734	yhaJ	atpA	No	-	
b3105	b4502	yhaJ	yeiW	No	-	
b3126	b2012	garL	yeeD	No	-	
b3137	b1922	kbaY	fliA	No	-	
b3157	b0044	yhbT	fixX	No	-	
b3160	b1831	yhbW	proQ	No	-	
b3183	b3021	obgE	mqsA	Yes	-	
b3187	b3021	ispB	mqsA	No	-	
b3189	b2012	murA	yeeD	No	-	
b3189	b3043	murA	ygiL	No	-	
b3204	b2533	ptsN	suhB	No	-	
b3204	b2646	ptsN	ypjF	No	-	
b3204	b2980	ptsN	glcC	No	-	
b3204	b3262	ptsN	yhdJ	No	-	
b3207	b3488	yrbL	yhiJ	No	-	
b3209	b0044	elbB	fixX	No	-	
b3209	b2440	elbB	eutC	No	-	
b3209	b0296	elbB	ykgM	No	-	
b3209	b3262	elbB	yhdJ	No	-	
b3211	b2319	yhcC	usg	No	-	YhcC
b3214	b2533	gltF	suhB	No	-	
b3215	b3357	yhcA	crp	No	-	
b3219	b2533	yhcF	suhB	No	-	
b3219	b2646	yhcF	ypjF	No	-	
b3228	b2646	sspB	ypjF	No	-	
b3229	b0731	sspA	mngA	Yes	340	
b3229	b3229	sspA	sspA	Yes	340	
b3236	b2460	mdh	eutQ	No	-	EutQ
b3248	b3129	yhdE	prlF	No	-	
b3256	b2316	accC	accD	Yes	-	
b3256	b2421	accC	cysM	Yes	-	

Continued on next page

Table S3: **Locus tag and gene id of detected protein interaction in Y2H data.** Locus tag and gene name of interacting proteins are listed in the first four columns. The existence of the interaction in other PPI networks is shown in the fifth column. The sixth and seventh column highlights whether the interacting proteins has been detected in a PPI module and whether one of the proteins is a central protein in a PPI module. (Continued from previous page)

Locus tag1	Locus tag2	Gene1	Gene2	Existing in other data sources	Id of a detected module	Centeral protein
b3256	b3256	accC	accC	Yes	65	
b3259	b3264	prmA	envR	No	-	PrmA
b3264	b1399	envR	paaX	No	-	
b3264	b1541	envR	ydfZ	No	-	YdfZ
b3264	b2533	envR	suhB	No	-	
b3264	b3262	envR	yhdJ	No	-	
b3281	b0564	aroE	appY	No	-	
b3281	b3651	aroE	trmH	No	-	
b3281	b3262	aroE	yhdJ	No	-	
b3284	b1108	smg	ycfP	No	11	
b3287	b0662	def	ubiF	No	-	
b3287	b2646	def	ypjF	No	-	
b3287	b3651	def	trmH	No	-	
b3288	b1831	fmt	proQ	No	-	
b3302	b0775	rpmD	bioB	No	-	
b3302	b2273	rpmD	yfbN	No	-	YfbN
b3304	b0574	rplR	cusB	No	-	
b3304	b3866	rplR	yihI	No	-	
b3306	b3065	rpsH	rpsU	Yes	17	
b3306	b3186	rpsH	rplU	No	-	RplU
b3306	b3253	rpsH	acuI	No	-	
b3306	b3296	rpsH	rpsD	Yes	-	
b3306	b3301	rpsH	rplO	No	-	RplO
b3306	b3306	rpsH	rpsH	Yes	17	
b3306	b3316	rpsH	rpsS	Yes	17	
b3306	b3317	rpsH	rplB	No	-	RplB
b3306	b3480	rpsH	nikE	No	-	
b3306	b4202	rpsH	rpsR	Yes	17	
b3306	b1432	rpsH	insQ	No	-	
b3316	b2608	rpsS	rimM	No	-	
b3329	b1399	gspH	paaX	No	-	
b3329	b2374	gspH	frc	No	-	
b3329	b3005	gspH	exbD	No	-	
b3340	b2473	fusA	ypfH	Yes	5	FusA
b3352	b0628	yheS	lipA	No	-	
b3352	b3408	yheS	feoA	No	-	
b3361	b0470	fic	dnaX	No	-	
b3361	b2001	fic	yeeR	Yes	329	
b3361	b3361	fic	fic	No	329	
b3361	b3362	fic	yhfG	Yes	329	
b3361	b1046	fic	clsC	No	-	
b3365	b3414	nirB	nfuA	No	-	

Continued on next page

Table S3: **Locus tag and gene id of detected protein interaction in Y2H data.** Locus tag and gene name of interacting proteins are listed in the first four columns. The existence of the interaction in other PPI networks is shown in the fifth column. The sixth and seventh column highlights whether the interacting proteins has been detected in a PPI module and whether one of the proteins is a central protein in a PPI module. (Continued from previous page)

Locus tag1	Locus tag2	Gene1	Gene2	Existing in other data sources	Id of a detected module	Centeral protein
b3374	b1421	frlD	trg	No	-	
b3374	b2975	frlD	glcA	No	53	
b3374	b3247	frlD	rng	No	-	
b3374	b3374	frlD	frlD	No	53	
b3374	b3386	frlD	rpe	Yes	53	Rpe
b3389	b0044	aroB	fixX	No	-	
b3389	b0719	aroB	ybgD	No	-	
b3389	b2012	aroB	yeeD	No	-	
b3389	b3043	aroB	ygiL	No	-	
b3389	b3389	aroB	aroB	No	5	
b3400	b4505	hslR	insX	No	-	
b3400	b0470	hslR	dnaX	No	-	
b3400	b0601	hslR	ybdM	No	-	
b3400	b0724	hslR	sdhB	No	-	
b3400	b1176	hslR	minC	No	-	
b3400	b2105	hslR	rcnR	No	-	
b3400	b2173	hslR	yeiR	No	-	
b3400	b2440	hslR	eutC	No	-	
b3400	b2980	hslR	glcC	No	-	
b3400	b3400	hslR	hslR	Yes	450	
b3400	b3418	hslR	malT	No	-	
b3400	b1831	hslR	proQ	No	-	
b3400	b3357	hslR	crp	No	-	
b3407	b3407	yhgF	yhgF	No	417	
b3409	b3409	feoB	feoB	No	165	
b3410	b0975	feoC	hyaD	No	-	
b3410	b4379	feoC	yjjW	No	-	
b3418	b0601	malT	ybdM	No	-	
b3418	b1394	malT	paaG	No	-	
b3418	b2440	malT	eutC	No	-	
b3418	b2699	malT	recA	No	-	RecA
b3418	b3380	malT	yhfW	No	-	
b3418	b3418	malT	malT	Yes	493	
b3418	b3357	malT	crp	No	-	
b3439	b4342	yhhW	yjiT	No	-	
b3448	b3448	yhhA	yhhA	No	253	
b3449	b1183	ugpQ	umuD	No	-	
b3449	b2567	ugpQ	rnc	No	-	
b3449	b3385	ugpQ	gph	No	-	
b3454	b2608	livF	rimM	No	-	
b3454	b3042	livF	yqiC	No	-	
b3480	b3480	nikE	nikE	Yes	3	

Continued on next page

Table S3: **Locus tag and gene id of detected protein interaction in Y2H data.** Locus tag and gene name of interacting proteins are listed in the first four columns. The existence of the interaction in other PPI networks is shown in the fifth column. The sixth and seventh column highlights whether the interacting proteins has been detected in a PPI module and whether one of the proteins is a central protein in a PPI module. (Continued from previous page)

Locus tag1	Locus tag2	Gene1	Gene2	Existing in other data sources	Id of a detected module	Centeral protein
b3481	b0034	nikR	caiF	No	-	
b3481	b0044	nikR	fixX	No	-	
b3481	b2440	nikR	eutC	No	-	
b3481	b2667	nikR	ygaV	No	-	
b3481	b4367	nikR	fhuF	No	-	
b3481	b1831	nikR	proQ	No	-	
b3481	b4502	nikR	yeiW	No	-	
b3495	b1868	uspA	yecE	No	245	
b3495	b3495	uspA	uspA	Yes	245	
b3498	b1133	prlC	mnmA	No	-	
b3501	b0977	arsR	hyaF	No	-	
b3507	b0188	dctR	tilS	No	12	
b3540	b2326	dppF	epmC	No	-	
b3543	b2130	dppB	yehY	No	-	
b3549	b0199	tag	metN	No	-	MetN
b3556	b0797	cspA	rhlE	No	-	RhlE
b3556	b1831	cspA	proQ	No	-	
b3559	b3560	glyS	glyQ	Yes	-	
b3560	b0060	glyQ	polB	No	-	
b3560	b0601	glyQ	ybdM	No	-	
b3560	b2997	glyQ	hybO	No	-	
b3560	b3559	glyQ	glyS	Yes	-	
b3560	b3262	glyQ	yhdJ	No	-	
b3561	b3561	wecH	wecH	No	370	
b3566	b3986	xylF	rplL	No	-	RplL
b3567	b4222	xylG	ytfP	No	-	
b3568	b0044	xylH	fixX	No	-	
b3568	b0530	xylH	sfmA	No	-	
b3568	b4398	xylH	creB	No	-	
b3574	b2980	yiaJ	glcC	No	1	
b3574	b3262	yiaJ	yhdJ	No	1	
b3583	b0034	yiaS	caiF	No	-	
b3583	b0044	yiaS	fixX	No	-	
b3583	b2149	yiaS	mglA	No	-	
b3583	b2713	yiaS	hydN	No	-	
b3583	b3185	yiaS	rpmA	No	-	
b3583	b3488	yiaS	yhiJ	No	-	
b3583	b3262	yiaS	yhdJ	No	-	
b3583	b4480	yiaS	hdfR	No	-	
b3592	b0044	yibF	fixX	No	-	
b3594	b2319	yibA	usg	Yes	-	YibA
b3594	b3594	yibA	yibA	Yes	128	YibA

Continued on next page

Table S3: **Locus tag and gene id of detected protein interaction in Y2H data.** Locus tag and gene name of interacting proteins are listed in the first four columns. The existence of the interaction in other PPI networks is shown in the fifth column. The sixth and seventh column highlights whether the interacting proteins has been detected in a PPI module and whether one of the proteins is a central protein in a PPI module. (Continued from previous page)

Locus tag1	Locus tag2	Gene1	Gene2	Existing in other data sources	Id of a detected module	Centeral protein
b3594	b3357	yibA	crp	No	-	YibA
b3594	b1597	yibA	asr	Yes	128	YibA
b3601	b2415	mtlR	ptsH	No	-	
b3601	b2646	mtlR	ypjF	No	-	
b3601	b3357	mtlR	crp	No	-	
b3608	b3608	gpsA	gpsA	Yes	176	
b3617	b3617	kbl	kbl	Yes	32	
b3633	b0719	waaA	ybgD	No	-	
b3635	b1881	mutM	cheZ	No	-	
b3635	b2285	mutM	nuoE	No	-	
b3635	b3129	mutM	prlF	No	-	
b3635	b4379	mutM	yjjW	No	-	
b3635	b2714	mutM	ascG	No	-	
b3645	b0245	dinD	ykfI	No	-	
b3645	b2522	dinD	sseB	No	-	
b3651	b3651	trmH	trmH	Yes	128	
b3661	b3651	nlpA	trmH	No	-	
b3669	b3043	uhpA	ygiL	No	-	
b3669	b0946	uhpA	zapC	No	-	
b3669	b1831	uhpA	proQ	No	-	
b3670	b4379	ilvN	yjjW	No	-	
b3670	b2326	ilvN	epmC	No	-	
b3671	b3671	ilvB	ilvB	Yes	170	
b3674	b3032	yidF	cpdA	No	-	
b3680	b0998	yidL	torD	No	-	
b3686	b3686	ibpB	ibpB	Yes	29	
b3700	b1831	recF	proQ	No	-	
b3701	b0172	dnaN	frr	No	-	DnaN
b3701	b0640	dnaN	holA	Yes	-	DnaN
b3701	b2181	dnaN	yejG	No	-	DnaN
b3702	b2361	dnaA	yfdR	No	-	
b3704	b0119	rnpA	yacL	No	-	
b3706	b2907	mnmE	ubiH	No	-	
b3708	b0477	tnaA	gsk	No	-	
b3708	b3708	tnaA	tnaA	Yes	364	
b3723	b0464	bglG	acrR	No	-	BglG
b3723	b0470	bglG	dnaX	No	-	BglG
b3723	b2440	bglG	eutC	No	-	BglG
b3723	b3723	bglG	bglG	Yes	157	BglG
b3723	b0946	bglG	zapC	No	-	BglG
b3730	b3730	glmU	glmU	Yes	20	
b3731	b3731	atpC	atpC	Yes	38	

Continued on next page

Table S3: **Locus tag and gene id of detected protein interaction in Y2H data.** Locus tag and gene name of interacting proteins are listed in the first four columns. The existence of the interaction in other PPI networks is shown in the fifth column. The sixth and seventh column highlights whether the interacting proteins has been detected in a PPI module and whether one of the proteins is a central protein in a PPI module. (Continued from previous page)

Locus tag1	Locus tag2	Gene1	Gene2	Existing in other data sources	Id of a detected module	Centeral protein
b3732	b3731	atpD	atpC	Yes	38	
b3732	b3734	atpD	atpA	Yes	38	
b3733	b1399	atpG	paaX	No	-	
b3733	b2646	atpG	ypjF	No	-	
b3733	b2980	atpG	glcC	No	-	
b3733	b3520	atpG	yhjB	No	-	YhjB
b3733	b3731	atpG	atpC	Yes	38	
b3733	b4367	atpG	fhuF	No	-	
b3734	b0172	atpA	frr	No	-	
b3734	b1156	atpA	tfaE	No	-	
b3734	b3484	atpA	yhhI	No	-	
b3734	b3731	atpA	atpC	Yes	38	
b3734	b3732	atpA	atpD	Yes	38	
b3734	b0644	atpA	ybeQ	No	-	
b3734	b4342	atpA	yjiT	No	-	
b3735	b0286	atpH	paoA	No	-	
b3735	b0470	atpH	dnaX	No	-	
b3735	b1070	atpH	flgN	No	-	
b3735	b1881	atpH	cheZ	No	-	
b3735	b2105	atpH	rcnR	No	-	
b3735	b2440	atpH	eutC	No	-	
b3740	b2646	rsmG	ypjF	No	-	
b3742	b2980	mioC	glcC	No	-	
b3744	b3744	asnA	asnA	Yes	44	
b3749	b0172	rbsA	frr	No	-	
b3749	b0809	rbsA	glnQ	No	-	
b3749	b2755	rbsA	ygbT	No	-	
b3749	b3021	rbsA	mqsA	No	-	
b3749	b3749	rbsA	rbsA	Yes	335	
b3749	b3262	rbsA	yhdJ	No	-	
b3753	b3021	rbsR	mqsA	No	-	
b3764	b0215	yifE	dnaQ	No	-	
b3764	b0464	yifE	acrR	No	-	
b3764	b3043	yifE	ygiL	No	-	
b3764	b3825	yifE	pldB	No	-	
b3774	b0760	ilvC	modF	No	-	
b3775	b3775	ppiC	ppiC	Yes	478	
b3783	b2988	rho	gss	Yes	102	
b3783	b3783	rho	rho	Yes	102	
b3783	b3910	rho	yiiM	Yes	102	
b3806	b0223	cyaA	yafJ	No	-	
b3806	b0962	cyaA	helD	No	-	

Continued on next page

Table S3: **Locus tag and gene id of detected protein interaction in Y2H data.** Locus tag and gene name of interacting proteins are listed in the first four columns. The existence of the interaction in other PPI networks is shown in the fifth column. The sixth and seventh column highlights whether the interacting proteins has been detected in a PPI module and whether one of the proteins is a central protein in a PPI module. (Continued from previous page)

Locus tag1	Locus tag2	Gene1	Gene2	Existing in other data sources	Id of a detected module	Centeral protein
b3806	b1264	cyaA	trpE	No	-	TrpE
b3806	b3021	cyaA	mqsA	No	-	
b3806	b3129	cyaA	prlF	No	-	
b3806	b3773	cyaA	ilvY	No	-	
b3806	b1198	cyaA	dhaM	No	-	DhaM
b3806	b3262	cyaA	yhdJ	No	-	
b3806	b3357	cyaA	crp	No	-	
b3810	b3967	yigA	murI	No	-	
b3811	b0064	xerC	araC	No	-	
b3811	b0851	xerC	nfsA	No	-	
b3811	b2980	xerC	glcC	No	-	
b3811	b4379	xerC	yjjW	No	-	
b3811	b1362	xerC	rzpR	No	-	
b3843	b3492	ubiD	yhiN	No	72	
b3867	b4099	hemN	phnI	Yes	-	
b3867	b0376	hemN	ampH	No	-	
b3869	b1156	glnL	tfaE	No	-	
b3869	b2980	glnL	glcC	No	-	
b3869	b0535	glnL	fimZ	No	-	
b3870	b2755	glnA	ygbT	No	-	
b3872	b0724	yihL	sdhB	No	-	
b3872	b1176	yihL	minC	No	-	
b3872	b3021	yihL	mqsA	No	-	
b3872	b4367	yihL	fhuF	No	-	
b3872	b0855	yihL	potG	No	-	
b3877	b2755	yihP	ygbT	No	-	
b3911	b1399	cpxA	paaX	No	-	
b3912	b3911	cpxR	cpxA	Yes	315	
b3919	b0903	tpiA	pflB	Yes	140	
b3919	b1127	tpiA	pepT	Yes	-	
b3921	b3357	yiiR	crp	No	-	
b3923	b1264	uspD	trpE	No	-	TrpE
b3923	b3495	uspD	uspA	Yes	245	
b3929	b0470	rraA	dnaX	No	-	
b3929	b0601	rraA	ybdM	No	-	
b3929	b3357	rraA	crp	No	-	
b3933	b1399	ftsN	paaX	No	-	
b3933	b4379	ftsN	yjjW	No	-	
b3934	b2456	cytR	eutN	No	-	
b3934	b2667	cytR	ygaV	No	-	
b3934	b2980	cytR	glcC	No	-	
b3934	b3021	cytR	mqsA	No	-	

Continued on next page

Table S3: **Locus tag and gene id of detected protein interaction in Y2H data.** Locus tag and gene name of interacting proteins are listed in the first four columns. The existence of the interaction in other PPI networks is shown in the fifth column. The sixth and seventh column highlights whether the interacting proteins has been detected in a PPI module and whether one of the proteins is a central protein in a PPI module. (Continued from previous page)

Locus tag1	Locus tag2	Gene1	Gene2	Existing in other data sources	Id of a detected module	Centeral protein
b3936	b0399	rpmE	phoB	No	-	
b3936	b0775	rpmE	bioB	No	-	
b3936	b1156	rpmE	tfaE	No	-	
b3936	b2456	rpmE	eutN	No	-	
b3946	b2184	fsaB	yejH	No	-	
b3946	b3513	fsaB	mdtE	No	-	
b3950	b2012	frwB	yeeD	No	-	
b3950	b2334	frwB	yfcQ	No	-	
b3950	b2980	frwB	glcC	No	-	
b3954	b0650	yijO	hscC	No	-	
b3954	b0779	yijO	uvrB	No	-	
b3954	b3953	yijO	frwD	No	-	
b3958	b0719	argC	ybgD	No	-	
b3972	b0044	murB	fixX	No	-	
b3972	b1831	murB	proQ	No	-	
b3981	b3067	secE	rpoD	No	-	
b3983	b1362	rplK	rzpR	No	-	
b3987	b0059	rpoB	rapA	No	-	RpoB
b4025	b4025	pgi	pgi	Yes	52	
b4041	b1620	plsB	malI	No	-	
b4048	b1105	yjbM	lpoB	No	-	
b4063	b4505	soxR	insX	No	-	
b4063	b0895	soxR	dmsB	No	-	
b4076	b2957	nrfG	ansB	No	-	
b4079	b0873	fdhF	hcp	No	-	
b4081	b1713	mdtO	pheT	No	-	
b4081	b4081	mdtO	mdtO	No	-	
b4088	b2440	alsB	eutC	No	-	
b4088	b1560	alsB	ydfU	No	-	
b4101	b3749	phnG	rbsA	No	-	
b4141	b1372	yjeH	stfR	No	111	
b4158	b0199	yjeO	metN	No	-	MetN
b4158	b3304	yjeO	rplR	No	-	
b4158	b0946	yjeO	zapC	No	-	
b4167	b4180	nnr	rlmB	No	-	
b4170	b4170	mutL	mutL	Yes	528	
b4173	b2283	hflX	nuoG	No	-	
b4181	b4181	yjfI	yjfI	No	83	
b4191	b2584	ulaR	pka	Yes	-	UlaR
b4202	b4200	rpsR	rpsF	Yes	-	
b4227	b0463	ytfQ	acrA	No	-	
b4227	b0775	ytfQ	bioB	No	-	

Continued on next page

Table S3: **Locus tag and gene id of detected protein interaction in Y2H data.** Locus tag and gene name of interacting proteins are listed in the first four columns. The existence of the interaction in other PPI networks is shown in the fifth column. The sixth and seventh column highlights whether the interacting proteins has been detected in a PPI module and whether one of the proteins is a central protein in a PPI module. (Continued from previous page)

Locus tag1	Locus tag2	Gene1	Gene2	Existing in other data sources	Id of a detected module	Centeral protein
b4227	b1156	ytfQ	tfaE	No	-	
b4227	b2456	ytfQ	eutN	No	-	
b4227	b0644	ytfQ	ybeQ	No	-	
b4227	b0872	ytfQ	hcr	No	-	
b4233	b0095	mpl	ftsZ	No	-	
b4233	b3106	mpl	yhaK	No	-	
b4233	b4233	mpl	mpl	No	134	
b4234	b3825	yjgA	pldB	No	-	
b4237	b4237	nrdG	nrdG	Yes	169	
b4237	b4238	nrdG	nrdD	Yes	169	NrdD
b4238	b0864	nrdD	artP	No	-	NrdD
b4238	b3992	nrdD	thiF	No	-	NrdD
b4238	b4238	nrdD	nrdD	Yes	169	NrdD
b4239	b0537	treC	intD	No	-	
b4241	b2012	treR	yeeD	No	-	
b4241	b2980	treR	glcC	No	-	
b4258	b4258	valS	valS	Yes	40	
b4259	b4259	holC	holC	Yes	62	HolC
b4266	b1831	idnO	proQ	No	-	
b4266	b2123	idnO	yehR	No	-	
b4268	b0245	idnK	ykfI	No	-	
b4268	b0601	idnK	ybdM	No	-	
b4268	b2415	idnK	ptsH	No	-	
b4268	b2440	idnK	eutC	No	-	
b4268	b2646	idnK	ypjF	No	-	
b4268	b3067	idnK	rpoD	No	-	
b4268	b2714	idnK	ascG	No	-	
b4271	b1127	intB	pepT	No	-	
b4290	b0956	fecB	matP	No	-	
b4290	b1501	fecB	ydeP	No	-	
b4290	b1705	fecB	ydiE	No	-	
b4290	b2805	fecB	fucR	No	-	
b4290	b3721	fecB	bglB	No	-	
b4290	b1550	fecB	gnsB	No	-	
b4290	b2123	fecB	yehR	No	-	
b4301	b0088	sgcE	murD	Yes	185	
b4361	b4052	dnaC	dnaB	Yes	16	DnaB
b4376	b3472	osmY	dcrB	No	104	
b4377	b4377	yjjU	yjjU	No	641	
b4383	b1831	deoB	proQ	No	-	
b4389	b0610	radA	rnk	No	-	
b4391	b2980	yjjK	glcC	No	-	

Continued on next page

Table S3: **Locus tag and gene id of detected protein interaction in Y2H data.** Locus tag and gene name of interacting proteins are listed in the first four columns. The existence of the interaction in other PPI networks is shown in the fifth column. The sixth and seventh column highlights whether the interacting proteins has been detected in a PPI module and whether one of the proteins is a central protein in a PPI module. (Continued from previous page)

Locus tag1	Locus tag2	Gene1	Gene2	Existing in other data sources	Id of a detected module	Centeral protein
b0097	b1620	secM	malI	No	-	
b0125	b4505	hpt	insX	No	-	
b0125	b3513	hpt	mdtE	No	-	
b0125	b4126	hpt	yjdI	No	-	
b0125	b4502	hpt	yeiW	No	-	
b0125	[]	hpt	[]	No	-	
b0435	b0888	bolA	trxB	No	-	
b0435	b2130	bolA	yehY	Yes	-	
b0435	b2997	bolA	hybO	No	-	
b0435	b0435	bolA	bolA	Yes	404	
b0583	b3357	entD	crp	No	-	
b0655	b0652	gltI	gltL	Yes	303	
b0655	b0655	gltI	gltI	Yes	303	
b0740	b0425	tolB	panE	No	-	
b0740	b0464	tolB	acrR	No	-	
b0740	b0633	tolB	rlpA	No	-	
b0740	b0947	tolB	ycbX	No	-	
b0740	b1105	tolB	lpoB	No	-	
b0740	b2714	tolB	ascG	No	-	
b0794	b0041	ybhF	fixA	No	-	
b0872	b4505	hcr	insX	No	-	
b0872	b0601	hcr	ybdM	No	-	
b0872	b2285	hcr	nuoE	No	-	
b0872	b2868	hcr	xdhC	No	-	
b0872	b2997	hcr	hybO	No	-	
b0872	b3513	hcr	mdtE	No	-	
b0872	b4126	hcr	yjdI	No	-	
b0872	b4502	hcr	yeiW	No	-	
b0894	b2980	dmsA	glcC	No	-	
b0894	b3087	dmsA	ygjR	No	-	
b0894	b3825	dmsA	pldB	No	-	
b0946	b0464	zapC	acrR	No	-	
b0946	b0601	zapC	ybdM	No	-	
b0946	b1394	zapC	paaG	No	-	
b0946	b2980	zapC	glcC	No	-	
b0946	b4587	zapC	insN	No	-	
b0946	b0946	zapC	zapC	No	96	
b0946	b2714	zapC	ascG	No	-	
b0946	b3357	zapC	crp	No	-	
b0947	b2764	ycbX	cysJ	Yes	51	
b0947	b0947	ycbX	ycbX	Yes	51	
b0952	b0464	ymbA	acrR	No	-	

Continued on next page

Table S3: **Locus tag and gene id of detected protein interaction in Y2H data.** Locus tag and gene name of interacting proteins are listed in the first four columns. The existence of the interaction in other PPI networks is shown in the fifth column. The sixth and seventh column highlights whether the interacting proteins has been detected in a PPI module and whether one of the proteins is a central protein in a PPI module. (Continued from previous page)

Locus tag1	Locus tag2	Gene1	Gene2	Existing in other data sources	Id of a detected module	Centeral protein
b0952	b2173	ymbA	yeiR	No	-	
b0952	b4379	ymbA	yjjW	No	-	
b0952	b1391	ymbA	paaD	No	-	
b0968	b4502	yccX	yeiW	No	-	
b1060	b2440	bssS	eutC	No	-	
b1060	b3262	bssS	yhdJ	No	-	
b1105	b3986	lpoB	rplL	No	-	RplL
b1117	b0151	lolD	fhuC	No	-	
b1191	b4016	cvrA	aceK	No	-	
b1287	b0889	yciW	lrp	No	-	
b1287	b1275	yciW	cysB	No	-	
b1338	b3042	abgA	yqiC	No	-	
b1361	b4043	ydaW	lexA	No	-	
b1407	b0975	ydbD	hyaD	No	-	
b1407	b2325	ydbD	yfcL	No	-	
b1479	b1973	maeA	zinT	No	-	
b1479	b2335	maeA	yfcR	No	-	
b1479	b3215	maeA	yhcA	No	-	
b1624	b1624	ydgJ	ydgJ	Yes	73	
b1697	b1698	ydiQ	ydiR	No	-	
b1714	b0223	pheS	yafJ	No	-	
b1714	b0401	pheS	brnQ	Yes	405	
b1714	b0851	pheS	nfsA	No	-	
b1714	b1713	pheS	pheT	Yes	-	
b1714	b1990	pheS	erfK	Yes	405	
b1714	b3021	pheS	mqsA	No	-	
b1714	b4587	pheS	insN	No	-	
b1714	b1714	pheS	pheS	Yes	405	
b1772	b0199	ydjH	metN	No	-	MetN
b1772	b0964	ydjH	yccT	No	-	
b1786	b4081	yeaJ	mdtO	No	-	
b1836	b0422	yebV	xseB	No	-	
b1836	b1284	yebV	yciT	No	26	
b1836	b1658	yebV	purR	No	26	PurR
b1836	b1827	yebV	kdgR	Yes	26	KdgR
b1836	b3261	yebV	fis	No	-	
b1836	b1836	yebV	yebV	No	26	
b1836	b1953	yebV	yodD	Yes	26	
b1837	b2440	yebW	eutC	No	-	
b2107	b0819	rcnB	ybiS	Yes	169	
b2125	b0146	yehT	sfsA	No	31	SfsA, YehT
b2125	b0199	yehT	metN	No	-	MetN, YehT

Continued on next page

Table S3: **Locus tag and gene id of detected protein interaction in Y2H data.** Locus tag and gene name of interacting proteins are listed in the first four columns. The existence of the interaction in other PPI networks is shown in the fifth column. The sixth and seventh column highlights whether the interacting proteins has been detected in a PPI module and whether one of the proteins is a central protein in a PPI module. (Continued from previous page)

**Locus tag1**	**Locus tag2**	**Gene1**	**Gene2**	**Existing in other data sources**	**Id of a detected module**	**Centeral protein**
b2125	b2125	yehT	yehT	No	31	YehT
b2134	b2285	pbpG	nuoE	No	-	
b2134	b3021	pbpG	mqsA	No	-	
b2134	b3082	pbpG	higA	No	-	
b2134	b4016	pbpG	aceK	No	-	
b2134	b1105	pbpG	lpoB	No	-	
b2134	b2123	pbpG	yehR	No	-	
b2134	b2496	pbpG	hda	No	-	
b2134	b2511	pbpG	der	No	-	
b4502	b2980	yeiW	glcC	No	-	
b4502	b3239	yeiW	yhcO	No	-	
b2201	b0464	ccmA	acrR	No	-	
b2201	b3239	ccmA	yhcO	No	-	
b2264	b2264	menD	menD	Yes	188	
b2264	b3526	menD	kdgK	Yes	188	
b2496	b3021	hda	mqsA	No	-	
b2496	b2496	hda	hda	Yes	203	
b2608	b0077	rimM	ilvI	No	-	
b2608	b0601	rimM	ybdM	No	-	
b2608	b2907	rimM	ubiH	No	-	
b2608	b1831	rimM	proQ	No	-	
b2708	b4505	gutQ	insX	No	-	
b2712	b0199	hypF	metN	No	-	MetN
b2712	b0530	hypF	sfmA	No	-	
b2712	b0796	hypF	ybiH	No	-	
b2712	b1176	hypF	minC	No	-	
b2712	b1895	hypF	uspC	No	-	
b2712	b3304	hypF	rplR	No	-	
b2712	b0946	hypF	zapC	No	-	
b2714	b2714	ascG	ascG	No	31	
b2769	b2770	ygcQ	ygcR	No	-	
b2798	b1916	ygdG	sdiA	No	-	
b2844	b2844	yqeF	yqeF	No	20	
b2909	b2646	ygfB	ypjF	No	-	
b2909	b2980	ygfB	glcC	No	-	
b2909	b3180	ygfB	yhbY	No	-	
b2909	b1831	ygfB	proQ	No	-	
b2909	b3262	ygfB	yhdJ	No	-	
b2909	b3825	ygfB	pldB	No	-	
b2953	b1156	yggU	tfaE	No	-	
b2953	b2456	yggU	eutN	No	-	
b3076	b0356	ebgA	frmA	No	-	

Continued on next page

Table S3: **Locus tag and gene id of detected protein interaction in Y2H data.** Locus tag and gene name of interacting proteins are listed in the first four columns. The existence of the interaction in other PPI networks is shown in the fifth column. The sixth and seventh column highlights whether the interacting proteins has been detected in a PPI module and whether one of the proteins is a central protein in a PPI module. (Continued from previous page)

Locus tag1	Locus tag2	Gene1	Gene2	Existing in other data sources	Id of a detected module	Centeral protein
b3076	b1080	ebgA	flgI	No	-	
b3076	b1670	ebgA	ydhU	No	-	
b3076	b1850	ebgA	eda	No	-	
b3076	b2622	ebgA	intA	Yes	-	IntA
b3162	b3162	deaD	deaD	Yes	66	DeaD
b3191	b0463	mlaB	acrA	No	-	
b3191	b1703	mlaB	ppsR	No	-	
b3210	b2181	arcB	yejG	No	-	
b3210	b1117	arcB	lolD	No	-	
b3270	b0185	yhdY	accA	No	-	
b3270	b2316	yhdY	accD	No	-	
b3998	b3998	nfi	nfi	Yes	274	
b3967	b0223	murI	yafJ	No	-	
b3967	b0610	murI	rnk	No	-	
b3967	b1703	murI	ppsR	No	-	
b3967	b2980	murI	glcC	No	-	
b3967	b3540	murI	dppF	No	-	
b3967	b4379	murI	yjjW	No	-	
b3967	b3967	murI	murI	Yes	3	
b3910	b3910	yiiM	yiiM	No	102	
b3871	b0948	typA	rlmL	No	-	TypA
b3871	b1831	typA	proQ	No	-	TypA
b3871	b3871	typA	typA	Yes	90	TypA
b3856	b3856	mobB	mobB	Yes	92	
b3833	b1183	ubiE	umuD	No	-	
b3833	b3385	ubiE	gph	No	-	
b4481	b2105	wecF	rcnR	No	-	
b4481	b2676	wecF	nrdF	No	-	
b4481	b3357	wecF	crp	No	-	
b3778	b1831	rep	proQ	No	-	
b4480	b0223	hdfR	yafJ	No	-	
b4480	b0464	hdfR	acrR	No	-	
b4480	b1183	hdfR	umuD	No	-	
b4479	b4479	dgoR	dgoR	Yes	90	
b3554	b3550	yiaF	yiaC	No	-	
b3546	b4505	eptB	insX	No	-	
b3546	b2980	eptB	glcC	No	-	
b3546	b3021	eptB	mqsA	No	-	
b3546	b3357	eptB	crp	No	-	
b3533	b1631	bcsA	rsxG	No	-	
b3526	b3526	kdgK	kdgK	No	188	
b3497	b0044	rsmJ	fixX	No	-	

Continued on next page

Table S3: **Locus tag and gene id of detected protein interaction in Y2H data.** Locus tag and gene name of interacting proteins are listed in the first four columns. The existence of the interaction in other PPI networks is shown in the fifth column. The sixth and seventh column highlights whether the interacting proteins has been detected in a PPI module and whether one of the proteins is a central protein in a PPI module. (Continued from previous page)

Locus tag1	Locus tag2	Gene1	Gene2	Existing in other data sources	Id of a detected module	Centeral protein
b3497	b1156	rsmJ	tfaE	No	-	
b3497	b2012	rsmJ	yeeD	No	-	
b3497	b4379	rsmJ	yjjW	No	-	
b3497	b1831	rsmJ	proQ	No	-	
b3497	b2522	rsmJ	sseB	No	-	
b3497	b4342	rsmJ	yjiT	No	-	
b3472	b2086	dcrB	yegS	No	104	
b3472	b3472	dcrB	dcrB	No	104	
b3424	b0889	glpG	lrp	No	-	
b3375	b0464	frlR	acrR	No	-	
b3375	b2980	frlR	glcC	No	-	
b3375	b2714	frlR	ascG	No	-	
b3375	b3357	frlR	crp	No	-	
b4474	b2996	frlC	hybA	No	-	
b3357	b0119	crp	yacL	Yes	-	
b3357	b2255	crp	arnA	No	-	
b3357	b3982	crp	nusG	No	-	
b4039	b4039	ubiC	ubiC	Yes	56	
b4060	b0172	yjcB	frr	No	-	
b4060	b1156	yjcB	tfaE	No	-	
b4060	b2456	yjcB	eutN	No	-	
b4060	b1831	yjcB	proQ	No	-	
b4084	b4084	alsK	alsK	No	382	
b4097	b3492	phnK	yhiN	No	-	
b4097	b4096	phnK	phnL	No	52	
b4188	b0044	yjfN	fixX	No	-	
b4188	b1973	yjfN	zinT	No	-	
b4188	b2334	yjfN	yfcQ	No	-	
b4188	b2997	yjfN	hybO	No	-	
b4207	b2713	fklB	hydN	No	-	FklB
b4207	b2996	fklB	hybA	No	-	FklB
b4207	b4286	fklB	yjhV	No	-	FklB
b4207	b4207	fklB	fklB	Yes	233	FklB
b4352	b3495	yjiA	uspA	No	245	
b0073	b0077	leuB	ilvI	No	-	
b0073	b0223	leuB	yafJ	No	-	
b0685	b3021	ybfE	mqsA	No	-	
b0685	b1831	ybfE	proQ	No	-	
b0685	b3825	ybfE	pldB	No	-	
b1597	b0956	asr	matP	No	-	
b1597	b1580	asr	rspB	No	-	
b2891	b1916	prfB	sdiA	No	-	

Continued on next page

Table S3: **Locus tag and gene id of detected protein interaction in Y2H data.** Locus tag and gene name of interacting proteins are listed in the first four columns. The existence of the interaction in other PPI networks is shown in the fifth column. The sixth and seventh column highlights whether the interacting proteins has been detected in a PPI module and whether one of the proteins is a central protein in a PPI module. (Continued from previous page)

Locus tag1	Locus tag2	Gene1	Gene2	Existing in other data sources	Id of a detected module	Centeral protein
b3826	b1973	yigL	zinT	No	-	
b3826	b1831	yigL	proQ	No	-	
b4346	b4180	mcrB	rlmB	No	-	
b4346	b4346	mcrB	mcrB	Yes	532	
b4555	b0601	yicS	ybdM	No	-	
b4555	b0719	yicS	ybgD	No	-	
b4555	b2012	yicS	yeeD	No	-	
b4555	b1831	yicS	proQ	No	-	
b4555	b4502	yicS	yeiW	No	-	
b0467	b3042	priC	yqiC	No	-	
b1355	b2714	ydaG	ascG	No	-	
b0516	b0504	allC	allS	No	-	
b2725	b3749	hycA	rbsA	No	-	
b2154	b0551	yeiG	quuD	No	-	
b0817	b0935	mntR	ssuD	No	-	
b0662	b2326	ubiF	epmC	No	-	
b0662	b4505	ubiF	insX	No	-	
b0695	b3204	kdpD	ptsN	No	-	
b0727	b0116	sucB	lpd	Yes	-	
b0750	b0601	nadA	ybdM	No	-	
b1812	b3360	pabB	pabA	Yes	143	
b1939	b3360	fliG	pabA	No	-	
b1941	b3306	fliI	rpsH	No	-	
b1603	b3306	pntA	rpsH	No	-	
b1089	b3306	rpmF	rpsH	No	-	
b1559	b3262	quuQ	yhdJ	No	-	
b1559	b2582	quuQ	trxC	No	-	
b1559	b4043	quuQ	lexA	No	-	
b1258	b0779	yciF	uvrB	No	-	
b1450	b3077	mcbR	ebgC	No	-	
b1450	b3492	mcbR	yhiN	No	-	
b1638	b3479	pdxH	nikD	No	-	
b1523	b1099	yneG	holB	No	-	
b1523	b4181	yneG	yjfI	No	-	
b2066	b0607	udk	uspG	No	-	
b2079	b1926	baeR	fliT	No	-	
b2249	b3306	yfaY	rpsH	No	-	
b2869	b3306	ygeV	rpsH	No	-	
b2869	b3170	ygeV	rimP	No	-	
b1939	b2440	fliG	eutC	No	-	
b1939	b2714	fliG	ascG	No	-	
b1939	b0948	fliG	rlmL	No	-	

Continued on next page

Table S3: **Locus tag and gene id of detected protein interaction in Y2H data.** Locus tag and gene name of interacting proteins are listed in the first four columns. The existence of the interaction in other PPI networks is shown in the fifth column. The sixth and seventh column highlights whether the interacting proteins has been detected in a PPI module and whether one of the proteins is a central protein in a PPI module. (Continued from previous page)

Locus tag1	Locus tag2	Gene1	Gene2	Existing in other data sources	Id of a detected module	Centeral protein
b1945	b1939	fliM	fliG	Yes	6	
b2416	b2980	ptsI	glcC	No	-	
b2416	b0535	ptsI	fimZ	No	-	
b1945	b2325	fliM	yfcL	No	-	
b4109	b0700	yjdA	rhsC	No	-	
b4109	b1275	yjdA	cysB	No	-	
b2478	b2478	dapA	dapA	Yes	10	
b3723	b2701	bglG	mltB	No	-	BglG
b3470	b2530	tusA	iscS	Yes	-	IscS
b3448	b0881	yhhA	clpS	No	-	
b3698	b1390	yidB	paaC	No	-	
b3704	b3195	rnpA	mlaF	No	-	
b4558	b3314	yifL	rpsC	No	-	
b3742	b2382	mioC	ypdC	No	-	
b3742	b4118	mioC	melR	No	-	
b3764	b2334	yifE	yfcQ	No	-	
b3807	b2334	cyaY	yfcQ	No	-	
b3807	b1174	cyaY	minE	No	-	
b3858	b1973	yihD	zinT	No	-	
b3923	b0286	uspD	paoA	No	-	
b3923	b0530	uspD	sfmA	No	-	
b3923	b1973	uspD	zinT	No	-	
b3936	b0835	rpmE	rimO	No	-	
b3964	b0835	yijD	rimO	No	-	
b3964	b2456	yijD	eutN	No	-	
b3922	b1585	yiiS	ynfC	No	-	
b3922	b2456	yiiS	eutN	No	-	
b3922	b1174	yiiS	minE	No	-	
b3995	b2415	rsd	ptsH	No	-	
b3781	b0041	trxA	fixA	No	-	
b3781	b2562	trxA	yfhL	No	-	
b3781	b2997	trxA	hybO	No	-	
b3781	b4367	trxA	fhuF	No	-	
b3781	b2720	trxA	hycF	No	-	
b3378	b1585	yhfU	ynfC	No	-	
b0058	b0885	rluA	aat	No	-	
b0058	b3043	rluA	ygiL	No	-	
b0058	b4342	rluA	yjiT	No	-	
b0058	b0398	rluA	sbcD	No	-	
b0038	b4011	caiB	yjaA	No	-	
b0049	b0719	apaH	ybgD	No	-	
b0049	b0530	apaH	sfmA	No	-	

Continued on next page

Table S3: **Locus tag and gene id of detected protein interaction in Y2H data.** Locus tag and gene name of interacting proteins are listed in the first four columns. The existence of the interaction in other PPI networks is shown in the fifth column. The sixth and seventh column highlights whether the interacting proteins has been detected in a PPI module and whether one of the proteins is a central protein in a PPI module. (Continued from previous page)

Locus tag1	Locus tag2	Gene1	Gene2	Existing in other data sources	Id of a detected module	Centeral protein
b0052	b0052	pdxA	pdxA	Yes	331	
b0052	b3043	pdxA	ygiL	No	-	
b0052	b0719	pdxA	ybgD	No	-	
b0059	b0948	rapA	rlmL	No	-	
b0059	b3262	rapA	yhdJ	No	-	
b0059	b2980	rapA	glcC	No	-	
b0064	b3043	araC	ygiL	No	-	
b0064	b0719	araC	ybgD	No	-	
b0064	b1837	araC	yebW	No	-	
b0067	b0719	thiP	ybgD	No	-	
b0076	b3256	leuO	accC	No	-	
b0076	b2079	leuO	baeR	No	-	
b0076	b1105	leuO	lpoB	No	-	
b0080	b0080	cra	cra	Yes	262	
b0088	b0450	murD	glnK	No	-	
b0098	b0719	secA	ybgD	No	-	SecA
b0092	b0530	ddlB	sfmA	No	-	
b0092	b0719	ddlB	ybgD	No	-	
b0099	b3043	mutT	ygiL	No	-	
b0099	b0719	mutT	ybgD	No	-	
b3352	b1778	yheS	msrB	No	-	
b3352	b2334	yheS	yfcQ	No	-	
b3352	b2335	yheS	yfcR	No	-	
b3352	b0530	yheS	sfmA	No	-	
b3431	b0719	glgX	ybgD	No	-	
b3431	b2334	glgX	yfcQ	No	-	
b3431	b1585	glgX	ynfC	No	-	
b3431	b3043	glgX	ygiL	No	-	
b3431	b4219	glgX	msrA	No	-	
b3431	b0530	glgX	sfmA	No	-	
b3671	b3670	ilvB	ilvN	Yes	170	
b3722	b3170	bglF	rimP	No	-	
b4297	b4297	yjhG	yjhG	Yes	3	
b4006	b4006	purH	purH	No	206	
b3189	b0530	murA	sfmA	No	-	
b3197	b3043	kdsD	ygiL	No	-	
b3197	b0719	kdsD	ybgD	No	-	
b0578	b0578	nfsB	nfsB	Yes	214	
b0587	b1200	fepE	dhaK	No	-	
b0587	b0043	fepE	fixC	No	-	
b0587	b0006	fepE	yaaA	No	-	
b0587	b1604	fepE	ydgH	No	-	

Continued on next page

Table S3: **Locus tag and gene id of detected protein interaction in Y2H data.** Locus tag and gene name of interacting proteins are listed in the first four columns. The existence of the interaction in other PPI networks is shown in the fifth column. The sixth and seventh column highlights whether the interacting proteins has been detected in a PPI module and whether one of the proteins is a central protein in a PPI module. (Continued from previous page)

Locus tag1	Locus tag2	Gene1	Gene2	Existing in other data sources	Id of a detected module	Centeral protein
b3749	b3418	rbsA	malT	No	-	
b4093	b0413	phnO	nrdR	No	-	
b4244	b4245	pyrI	pyrB	Yes	578	
b4302	b2701	sgcA	mltB	No	-	
b4302	b1013	sgcA	rutR	No	-	
b4302	b4505	sgcA	insX	No	-	
b4302	b1176	sgcA	minC	No	-	
b4302	b3982	sgcA	nusG	No	-	
b4302	b2714	sgcA	ascG	No	-	
b4393	b2334	trpR	yfcQ	No	-	
b1355	b2701	ydaG	mltB	No	-	
b1355	b4299	ydaG	yjhI	No	-	
b1355	b4379	ydaG	yjjW	No	-	
b0151	b4011	fhuC	yjaA	No	-	
b0320	b0319	yahF	yahE	No	-	
b0347	b0321	mhpA	yahG	No	-	
b0717	b2696	ybgP	csrA	No	-	
b0719	b2123	ybgD	yehR	No	-	
b0721	b1698	sdhC	ydiR	No	-	
b2809	b3314	ygdI	rpsC	No	-	
b4217	b4062	ytfK	soxS	No	-	
b4217	b1604	ytfK	ydgH	No	-	
b3466	b1973	yhhL	zinT	No	-	
b0968	b1174	yccX	minE	No	-	
b0968	b0530	yccX	sfmA	No	-	
b2754	b2334	ygbF	yfcQ	No	-	
b2754	b1973	ygbF	zinT	No	-	
b2953	b1585	yggU	ynfC	No	-	
b1362	b0998	rzpR	torD	No	-	
b0435	b4379	bolA	yjjW	No	-	
b0435	b2415	bolA	ptsH	No	-	
b0435	b1390	bolA	paaC	No	-	
b0435	b4299	bolA	yjhI	No	-	
b0435	b2562	bolA	yfhL	No	-	
b1117	b3925	lolD	glpX	No	-	
b0938	b1585	elfA	ynfC	No	-	
b2702	b1391	srlA	paaD	No	-	
b2135	b3540	yohC	dppF	No	-	
b2135	b3043	yohC	ygiL	No	-	
b0294	b2770	ecpR	ygcR	No	-	
b0795	b0719	ybhG	ybgD	No	-	
b4331	b0719	kptA	ybgD	No	-	

Continued on next page

Table S3: **Locus tag and gene id of detected protein interaction in Y2H data.** Locus tag and gene name of interacting proteins are listed in the first four columns. The existence of the interaction in other PPI networks is shown in the fifth column. The sixth and seventh column highlights whether the interacting proteins has been detected in a PPI module and whether one of the proteins is a central protein in a PPI module. (Continued from previous page)

Locus tag1	Locus tag2	Gene1	Gene2	Existing in other data sources	Id of a detected module	Centeral protein
b4331	b3043	kptA	ygiL	No	-	
b4331	b0530	kptA	sfmA	No	-	
b4342	b4161	yjiT	rsgA	No	-	RsgA
b4206	b3043	ytfB	ygiL	No	-	
b4206	b0719	ytfB	ybgD	No	-	
b3067	b0064	rpoD	araC	No	-	
b4043	b0064	lexA	araC	No	-	
b3067	b3905	rpoD	rhaS	Yes	-	RhaS
b3067	b3906	rpoD	rhaR	Yes	105	
b3067	b4118	rpoD	melR	Yes	-	
b3067	b1735	rpoD	chbR	No	-	
b0064	b0064	araC	araC	Yes	74	
b0064	b1735	araC	chbR	No	-	
b3906	b4043	rhaR	lexA	No	-	
b3906	b1735	rhaR	chbR	No	-	
b3067	b4043	rpoD	lexA	No	-	
b3067	b2382	rpoD	ypdC	No	-	
b4043	b4043	lexA	lexA	Yes	86	
b4043	b1735	lexA	chbR	No	-	
b1735	b3067	chbR	rpoD	No	-	
b1735	b4043	chbR	lexA	No	-	
b1735	b1735	chbR	chbR	No	1	
b2382	b3067	ypdC	rpoD	No	-	
b2382	b4043	ypdC	lexA	No	-	
b2382	b0064	ypdC	araC	No	-	
b2382	b2382	ypdC	ypdC	No	225	
b3905	b4043	rhaS	lexA	No	-	RhaS
b3905	b0064	rhaS	araC	No	-	RhaS
b3905	b1735	rhaS	chbR	No	-	RhaS
b3905	b2382	rhaS	ypdC	No	-	RhaS
b0026	b0975	ileS	hyaD	Yes	-	
b0026	b4253	ileS	yjgL	Yes	-	
b0642	b0139	leuS	htrE	Yes	-	
b2411	b0642	ligA	leuS	Yes	-	
b1274	b0642	topA	leuS	Yes	-	
b2411	b2411	ligA	ligA	Yes	181	
b2411	b0975	ligA	hyaD	No	-	
b2411	b3751	ligA	rbsB	No	-	
b1274	b2411	topA	ligA	No	-	
b1274	b0975	topA	hyaD	Yes	-	
b1274	b3751	topA	rbsB	No	-	
b3751	b2411	rbsB	ligA	No	-	

Continued on next page

Table S3: **Locus tag and gene id of detected protein interaction in Y2H data.** Locus tag and gene name of interacting proteins are listed in the first four columns. The existence of the interaction in other PPI networks is shown in the fifth column. The sixth and seventh column highlights whether the interacting proteins has been detected in a PPI module and whether one of the proteins is a central protein in a PPI module. (Continued from previous page)

**Locus tag1**	**Locus tag2**	**Gene1**	**Gene2**	**Existing in other data sources**	**Id of a detected module**	**Centeral protein**
b3751	b1274	rbsB	topA	No	-	
b3751	b0975	rbsB	hyaD	No	-	
b3751	b3751	rbsB	rbsB	Yes	335	
b3751	b4253	rbsB	yjgL	No	-	
b4253	b2411	yjgL	ligA	No	-	
b4253	b0975	yjgL	hyaD	No	-	
b4253	b3751	yjgL	rbsB	No	-	
b0139	b2411	htrE	ligA	No	-	
b0139	b0975	htrE	hyaD	No	-	
b0139	b3751	htrE	rbsB	No	-	
b0780	b2411	ybhK	ligA	No	-	YbhK
b0780	b1274	ybhK	topA	No	-	YbhK
b0780	b0975	ybhK	hyaD	No	-	YbhK
b0780	b3751	ybhK	rbsB	No	-	YbhK
b0780	b4253	ybhK	yjgL	No	-	YbhK
b0780	b0139	ybhK	htrE	No	-	YbhK
b0780	b0780	ybhK	ybhK	Yes	40	YbhK
b0026	b2411	ileS	ligA	No	-	
b0026	b0780	ileS	ybhK	No	-	YbhK
b0642	b0975	leuS	hyaD	No	-	
b0642	b3751	leuS	rbsB	No	-	
b0642	b0780	leuS	ybhK	No	-	YbhK
b0450	b0450	glnK	glnK	Yes	115	
b0450	b3869	glnK	glnL	Yes	115	
b0450	b0451	glnK	amtB	No	-	
b0167	b0450	glnD	glnK	Yes	-	
b2553	b2553	glnB	glnB	Yes	115	
b2553	b3869	glnB	glnL	Yes	115	
b2553	b0167	glnB	glnD	Yes	-	
b2553	b0451	glnB	amtB	No	-	
b2553	b4255	glnB	rraB	Yes	-	
b3869	b0167	glnL	glnD	No	-	
b3869	b3869	glnL	glnL	Yes	115	
b0451	b3869	amtB	glnL	No	-	
b4255	b3869	rraB	glnL	No	-	
b1225	b1224	narH	narG	Yes	92	
b1468	b1225	narZ	narH	Yes	-	
b1225	b3081	narH	fadH	Yes	-	
b1225	b1466	narH	narW	Yes	-	
b1224	b1467	narG	narY	Yes	-	
b3081	b1467	fadH	narY	Yes	163	
b1467	b1466	narY	narW	Yes	163	

Continued on next page

Table S3: **Locus tag and gene id of detected protein interaction in Y2H data.** Locus tag and gene name of interacting proteins are listed in the first four columns. The existence of the interaction in other PPI networks is shown in the fifth column. The sixth and seventh column highlights whether the interacting proteins has been detected in a PPI module and whether one of the proteins is a central protein in a PPI module. (Continued from previous page)

Locus tag1	Locus tag2	Gene1	Gene2	Existing in other data sources	Id of a detected module	Centeral protein
b0932	b1467	pepN	narY	Yes	-	
b1468	b0932	narZ	pepN	No	-	
b1468	b1466	narZ	narW	Yes	163	
b1224	b0932	narG	pepN	No	-	
b1224	b1466	narG	narW	Yes	-	
b3081	b0932	fadH	pepN	No	-	
b0932	b0932	pepN	pepN	Yes	413	
b1466	b0932	narW	pepN	No	-	
b1466	b1466	narW	narW	Yes	163	
b1466	b1468	narW	narZ	Yes	163	
b1466	b1224	narW	narG	Yes	-	
b1466	b3081	narW	fadH	No	163	
b1225	b0932	narH	pepN	No	-	
b0026	b3751	ileS	rbsB	Yes	-	
b0072	b0071	leuC	leuD	Yes	55	
b0082	b4171	rsmH	miaA	No	-	
b0091	b0091	murC	murC	Yes	370	
b0099	b4153	mutT	frdB	No	-	
b0467	b0779	priC	uvrB	No	-	
b0581	b2726	ybdK	hypA	No	-	
b0723	b2897	sdhA	cptB	No	-	YdhA
b1176	b1176	minC	minC	Yes	448	
b1225	b1467	narH	narY	Yes	-	
b1807	b3064	tsaB	tsaD	Yes	133	
b1887	b3262	cheW	yhdJ	No	-	
b1939	b1105	fliG	lpoB	No	-	
b1945	b1946	fliM	fliN	Yes	6	
b2382	b1735	ypdC	chbR	No	-	
b2553	b0450	glnB	glnK	Yes	115	
b3671	b3769	ilvB	ilvM	Yes	170	
b3936	b3021	rpmE	mqsA	No	-	
b3995	b3067	rsd	rpoD	Yes	-	
b4259	b4372	holC	holD	Yes	62	HolC
b4372	b4259	holD	holC	Yes	62	HolC
b0435	b2667	bolA	ygaV	No	-	
b1117	b2193	lolD	narP	No	-	
b2583	b3169	yfiP	nusA	No	-	
b0399	b0399	phoB	phoB	Yes	487	
b4399	b4398	creC	creB	Yes	313	
b3405	b0399	ompR	phoB	No	-	OmpR
b0571	b0571	cusR	cusR	No	82	CusR
b1969	b0571	yedW	cusR	No	-	CusR

Continued on next page

Table S3: **Locus tag and gene id of detected protein interaction in Y2H data.** Locus tag and gene name of interacting proteins are listed in the first four columns. The existence of the interaction in other PPI networks is shown in the fifth column. The sixth and seventh column highlights whether the interacting proteins has been detected in a PPI module and whether one of the proteins is a central protein in a PPI module. (Continued from previous page)

Locus tag1	Locus tag2	Gene1	Gene2	Existing in other data sources	Id of a detected module	Centeral protein
b4398	b4398	creB	creB	No	313	
b0695	b0694	kdpD	kdpE	Yes	462	
b2079	b2079	baeR	baeR	No	95	
b3303	b3318	rpsE	rplW	Yes	-	RpsE, RplW
b0014	b0473	dnaK	htpG	Yes	-	DnaK, HtpG
b1269	b0948	rluB	rlmL	Yes	2	RluB
b2610	b3464	ffh	ftsY	Yes	360	
b3464	b2610	ftsY	ffh	Yes	360	
b4372	b0470	holD	dnaX	Yes	-	
b1237	b2669	hns	stpA	Yes	97	
b2369	b2370	evgA	evgS	Yes	525	
b2576	b1269	srmB	rluB	Yes	2	RluB
b3256	b3255	accC	accB	Yes	65	
b1713	b1714	pheT	pheS	Yes	-	
b3783	b3982	rho	nusG	Yes	-	
b0033	b0033	carB	carB	Yes	51	
b0470	b0215	dnaX	dnaQ	Yes	36	
b2469	b2193	narQ	narP	Yes	435	
b4058	b0779	uvrA	uvrB	Yes	-	
b4259	b1099	holC	holB	Yes	-	HolC
b0640	b0184	holA	dnaE	Yes	-	DnaE
b3934	b3357	cytR	crp	Yes	421	
b3357	b3934	crp	cytR	Yes	421	
b4355	b1887	tsr	cheW	Yes	30	
b1221	b2469	narL	narQ	No	-	
b0215	b1842	dnaQ	holE	Yes	-	
b0014	b1000	dnaK	cbpA	Yes	-	DnaK
b0640	b0215	holA	dnaQ	Yes	-	
b3164	b2576	pnp	srmB	Yes	2	
b2180	b1246	yejF	oppD	Yes	-	
b1084	b2576	rne	srmB	Yes	2	
b1941	b1941	fliI	fliI	Yes	6	
b0723	b0724	sdhA	sdhB	Yes	46	SdhA
b2837	b2837	galR	galR	Yes	362	
b1763	b2892	topB	recJ	Yes	-	TopB
b2576	b2576	srmB	srmB	Yes	2	
b1946	b1946	fliN	fliN	Yes	6	
b3778	b3778	rep	rep	Yes	384	
b3418	b0015	malT	dnaJ	Yes	-	DnaJ
b1071	b1922	flgM	fliA	Yes	-	
b1938	b1939	fliF	fliG	Yes	6	
b2837	b2151	galR	galS	No	-	

Continued on next page

Table S3: **Locus tag and gene id of detected protein interaction in Y2H data.** Locus tag and gene name of interacting proteins are listed in the first four columns. The existence of the interaction in other PPI networks is shown in the fifth column. The sixth and seventh column highlights whether the interacting proteins has been detected in a PPI module and whether one of the proteins is a central protein in a PPI module. (Continued from previous page)

Locus tag1	Locus tag2	Gene1	Gene2	Existing in other data sources	Id of a detected module	Centeral protein
b3781	b0888	trxA	trxB	Yes	454	
b0098	b0736	secA	ybgC	Yes	-	SecA
b1089	b3637	rpmF	rpmB	Yes	2	RpmB
b4143	b3616	groL	tdh	Yes	1	GroL
b4143	b2614	groL	grpE	Yes	-	GroL
b3813	b3778	uvrD	rep	No	-	
b0098	b3175	secA	secG	Yes	7	SecA
b3701	b3701	dnaN	dnaN	Yes	15	DnaN
b0595	b0595	entB	entB	Yes	233	
b3741	b3706	mnmG	mnmE	Yes	-	
b1200	b1201	dhaK	dhaR	Yes	-	
b2213	b3067	ada	rpoD	Yes	105	
b3357	b3067	crp	rpoD	Yes	-	
b0199	b2719	metN	hycG	Yes	-	MetN
b1740	b3702	nadE	dnaA	Yes	-	NadE
b4372	b1763	holD	topB	Yes	-	TopB
b2207	b2206	napD	napA	Yes	-	
b2528	b2525	iscA	fdx	Yes	-	
b2529	b2529	iscU	iscU	Yes	58	IscU
b3701	b2496	dnaN	hda	Yes	-	DnaN
b4143	b3520	groL	yhjB	Yes	-	GroL
b2286	b2283	nuoC	nuoG	Yes	35	
b4143	b1704	groL	aroH	Yes	1	GroL
b0888	b2673	trxB	nrdH	No	-	
b0470	b4059	dnaX	ssb	No	-	
b3723	b0316	bglG	yahB	Yes	157	
b0600	b0184	ybdL	dnaE	Yes	36	DnaE
b0421	b1566	ispA	flxA	Yes	49	IspA
b3418	b0042	malT	fixB	Yes	-	
b3418	b1942	malT	fliJ	Yes	-	
b2950	b1854	yggR	pykA	Yes	436	
b1410	b2707	ynbC	srlR	Yes	-	
b0903	b2707	pflB	srlR	Yes	-	
b3418	b2517	malT	rlmN	Yes	-	RlmN
b1945	b1945	fliM	fliM	Yes	6	
b1884	b2573	cheR	rpoE	Yes	-	
b1946	b1941	fliN	fliI	Yes	6	
b0225	b0226	yafQ	dinJ	Yes	347	
b1286	b3303	rnb	rpsE	Yes	-	RpsE
b2838	b3912	lysA	cpxR	Yes	315	
b0918	b1649	kdsB	nemR	Yes	192	KdsB
b3813	b3829	uvrD	metE	Yes	-	

Continued on next page

Table S3: **Locus tag and gene id of detected protein interaction in Y2H data.** Locus tag and gene name of interacting proteins are listed in the first four columns. The existence of the interaction in other PPI networks is shown in the fifth column. The sixth and seventh column highlights whether the interacting proteins has been detected in a PPI module and whether one of the proteins is a central protein in a PPI module. (Continued from previous page)

Locus tag1	Locus tag2	Gene1	Gene2	Existing in other data sources	Id of a detected module	Centeral protein
b2222	b2595	atoA	bamD	Yes	-	
b2515	b0292	ispG	ecpB	Yes	-	
b3182	b3172	dacB	argG	Yes	135	
b3317	b3326	rplB	gspE	Yes	2	RplB
b3061	b3423	ttdA	glpR	Yes	330	
b3506	b3506	slp	slp	Yes	23	
b2574	b0750	nadB	nadA	Yes	331	
b3357	b0034	crp	caiF	No	-	
b3035	b0740	tolC	tolB	No	-	TolC
b2781	b2566	mazG	era	Yes	1	
b3983	b0172	rplK	frr	No	-	
b3357	b3418	crp	malT	No	-	
b3701	b0184	dnaN	dnaE	Yes	-	DnaN, DnaE
b1924	b1926	fliD	fliT	Yes	-	
b1807	b4168	tsaB	tsaE	No	-	
b2207	b2997	napD	hybO	No	-	
b3869	b3868	glnL	glnG	Yes	115	
b0695	b0695	kdpD	kdpD	Yes	462	
b0995	b0993	torR	torS	Yes	463	
b1130	b1130	phoP	phoP	No	452	
b3341	b1269	rpsG	rluB	Yes	-	RluB, RpsG
b0820	b1290	ybiT	sapF	Yes	-	
b2802	b2802	fucI	fucI	Yes	13	
b2751	b2752	cysN	cysD	Yes	427	
b3780	b3780	rhlB	rhlB	Yes	2	
b4143	b2731	groL	fhlA	Yes	-	GroL
b0078	b0078	ilvH	ilvH	Yes	492	
b2810	b2811	csdA	csdE	Yes	277	
b4051	b4051	qorA	qorA	No	-	
b0759	b0759	galE	galE	Yes	497	
b3256	b0414	accC	ribD	Yes	-	RibD
b2114	b3317	metG	rplB	Yes	-	RplB
b2500	b2500	purN	purN	No	44	
b3301	b2517	rplO	rlmN	Yes	-	RplO, RlmN
b2573	b2573	rpoE	rpoE	Yes	14	
b0084	b3208	ftsI	mtgA	No	-	
b3701	b3386	dnaN	rpe	Yes	-	DnaN, Rpe
b3729	b1411	glmS	ynbD	Yes	202	GlmS
b0397	b1410	sbcC	ynbC	Yes	-	
b2509	b1579	xseA	intQ	Yes	189	
b2616	b1435	recN	ydcP	Yes	8	
b4016	b1136	aceK	icd	Yes	357	

Continued on next page

Table S3: **Locus tag and gene id of detected protein interaction in Y2H data.** Locus tag and gene name of interacting proteins are listed in the first four columns. The existence of the interaction in other PPI networks is shown in the fifth column. The sixth and seventh column highlights whether the interacting proteins has been detected in a PPI module and whether one of the proteins is a central protein in a PPI module. (Continued from previous page)

**Locus tag1**	**Locus tag2**	**Gene1**	**Gene2**	**Existing in other data sources**	**Id of a detected module**	**Centeral protein**
b2017	b4539	yefM	yoeB	Yes	241	
b0595	b0594	entB	entE	No	-	
b0849	b3610	grxA	grxC	No	-	
b1120	b0959	cobB	sxy	Yes	126	
b4372	b1410	holD	ynbC	Yes	-	
b1176	b1613	minC	manA	Yes	-	
b1200	b3030	dhaK	parE	Yes	-	ParE
b0091	b4230	murC	ytfT	Yes	-	
b2319	b1854	usg	pykA	Yes	-	
b1082	b1070	flgK	flgN	Yes	-	
b1884	b4146	cheR	epmB	Yes	-	
b3315	b2232	rplV	ubiG	Yes	2	RplV
b3743	b1967	asnC	hchA	Yes	-	
b4143	b2385	groL	ypdF	Yes	-	GroL
b2679	b0286	proX	paoA	Yes	-	
b1235	b0382	rssB	iraP	No	-	
b1884	b1941	cheR	fliI	Yes	-	
b1926	b1926	fliT	fliT	Yes	309	
b2573	b1941	rpoE	fliI	Yes	-	
b1807	b4373	tsaB	rimI	No	-	
b3995	b3995	rsd	rsd	No	14	
b2719	b2719	hycG	hycG	Yes	150	
b2719	b0199	hycG	metN	Yes	-	MetN
b2719	b0817	hycG	mntR	Yes	-	
b4280	b4280	yjhC	yjhC	Yes	18	
b0817	b0199	mntR	metN	Yes	18	MetN
b0817	b0817	mntR	mntR	Yes	18	
b0817	b2719	mntR	hycG	Yes	-	
b0817	b0254	mntR	perR	Yes	18	
b0817	b3520	mntR	yhjB	Yes	18	YhjB
b0254	b0254	perR	perR	Yes	18	
b4280	b0817	yjhC	mntR	Yes	18	

Table S4 Co-expressed genes/proteins which are detected in the same PPI module and their biological functions.

PPI Module Number	Co-expressed Genes	Biological Function
Module 5	rzpR, ypfH, yecF, yidL, ydhZ, yobD, ypdI, ydjH, ydiV, ydfX, ydaS, frC, insN-2, yedF, yeaD, yhcA, yfjZ	Pathogenicity
Module 11	ycfP, gadC, wbbK, raiA, yqjD, smg, yjbJ, sgcX	
Module 20	acpP, acpS, fabH, ybgJ, fabG, glmU	Fatty acid biosynthesis
Module 24	dapB, gnd, gcvP, gcvH, gcvT, ysgA, ppc	
Module 24	acnB, ybhP, dadX, glcB, aldB, deoB	
Module 82	CusS, CusR	CusSR Two-Component Signal Transduction System, dicarboxylate-dependent
Module 92	mog,mobA, mobB, moeA	Mo-molybdopterin cofactor metabolic process
Module 93	yihL, yihU, yihV	O-antigen capsule regulation
Module 104	yegS, astC, osmY, gltA	Response to stress
Module 112	mtlD, ydeT, ydfI, yncM, yncI	Mannitol metabolic process
Module 119	envz, ompR	EnvZ Two-Component Signal Transduction System, osmotic responsive
Module 131	mtgA, recJ, recQ, sbcB, ssb, rnhA	DNA repair, SOS response
Module 187	narX, narL	NarX Two-Component Signal Transduction System, nitrate dependent
Module 228	rstA, rstB	RstBA Two-Component Signal Transduction System
Module 301	purA, purL, yigB, nadC	Nucleotide biosynthetic process
Module 315	cpxA, cpxR	CpxAR Two-Component Signal Transduction System
Module 329	fic, ymdC, yhfG	Stationary phase protiens
Module 435	narP, narQ	NarQ Two-Component Signal Transduction System, nitrate dependent
Module 452	phoP, phoQ	PhoQP Two-Component Signal Transduction System, magnesium-dependent
Module 454	trxA, trxB, ycdX	Thioredoxin-disulfide reductase activity
